# *SPOP* and *OTUD7A* Control *EWS–FLI1* Protein Stability to Govern Ewing Sarcoma Growth

**DOI:** 10.1002/advs.202004846

**Published:** 2021-06-01

**Authors:** Siyuan Su, Jianfeng Chen, Yao Jiang, Ying Wang, Tamara Vital, Jiaming Zhang, Christian Laggner, Kong T. Nguyen, Zhichuan Zhu, Alex W. Prevatte, Natalie K. Barker, Laura E. Herring, Ian J. Davis, Pengda Liu

**Affiliations:** ^1^ Lineberger Comprehensive Cancer Center The University of North Carolina at Chapel Hill Chapel Hill NC 27599 USA; ^2^ Department of Biochemistry and Biophysics School of Medicine The University of North Carolina at Chapel Hill Chapel Hill NC 27599 USA; ^3^ Department of Genetics The University of North Carolina at Chapel Hill Chapel Hill NC 27599 USA; ^4^ Department of Pediatrics The University of North Carolina at Chapel Hill Chapel Hill NC 27599 USA; ^5^ Atomwise Inc. San Francisco CA 94103 USA; ^6^ UNC Proteomics Core Facility Department of Pharmacology The University of North Carolina at Chapel Hill Chapel Hill NC 27599 USA; ^7^ Present address: Cancer Center Union Hospital Tongji Medical College Huazhong University of Science and Technology Wuhan 430022 China; ^8^ Present address: Department of Oral Medicine, Infection, and Immunity Harvard School of Dental Medicine Boston MA 02215 USA

**Keywords:** 7Ai, *CK1*, Ewing sarcoma, *EWS–FLI1*, *OTUD7A*, *SPOP*

## Abstract

Chromosomal translocation results in development of an Ewing sarcoma breakpoint region 1‐Friend leukemia integration 1 (*EWS–FLI1*) fusion oncogene in the majority of Ewing sarcoma. The persistent dependence of the tumor for this oncoprotein points to *EWS–FLI1* as an ideal drug target. Although *EWS–FLI1* transcriptional targets and binding partners are evaluated, the mechanisms regulating *EWS–FLI1* protein stability remain elusive. Speckle‐type POZ protein (*SPOP*) and OTU domain‐containing protein 7A (*OTUD7A*) are identified as the *bona fide* E3 ligase and deubiquitinase, respectively, that control *EWS–FLI1* protein turnover in Ewing sarcoma. *Casein kinase 1*‐mediated phosphorylation of the VTSSS degron in the *FLI1* domain enhances *SPOP* activity to degrade *EWS–FLI1*. Opposing this process, *OTUD7A* deubiquitinates and stabilizes *EWS–FLI1*. Depletion of *OTUD7A* in Ewing sarcoma cell lines reduces *EWS–FLI1* protein abundance and impedes Ewing sarcoma growth in vitro and in mice. Performing an artificial‐intelligence‐based virtual drug screen of a 4‐million small molecule library, 7Ai is identified as a potential *OTUD7A* catalytic inhibitor. 7Ai reduces *EWS–FLI1* protein levels and decreases Ewing sarcoma growth in vitro and in a xenograft mouse model. This study supports the therapeutic targeting of *OTUD7A* as a novel strategy for Ewing sarcoma bearing *EWS–FLI1* and related fusions, and may also be applicable to other cancers dependent on aberrant *FLI1* expression.

## Graphical abstract

The fusion oncogene Ewing sarcoma breakpoint region 1‐Friend leukemia integration 1 (*EWS–FLI1*) drives Ewing sarcoma tumorigenesis. This work identifies Speckle‐type POZ protein (*SPOP*) and OTU domain‐containing protein 7A (*OTUD7A*) as the E3 ligase and deubiquitinase, respectively, that regulate *EWS–FLI1* protein homeostasis. 7Ai, a putative *OTUD7A* catalytic inhibitor identified through virtual compound screening, reduces Ewing sarcoma cell growth and migration.

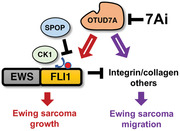

## 1 Introduction

Ewing sarcoma is an aggressive malignancy that develops in bones or soft tissues of children and young adults. A recurrent chromosomal translocation found in the majority of Ewing sarcoma fuses the Ewing sarcoma breakpoint region 1 or RNA‐binding protein EWS (*EWSR1*) and Friend leukemia integration 1 transcription factor (*FLI1*) genes generating an *EWS–FLI1* fusion protein. *EWS–FLI1* is the critical driver of Ewing sarcoma.^[^ ^1^ ^]^ Mechanistically, *EWS–FLI1* binds specific GGAA‐containing microsatellite regions to maintain nucleosome depletion.^[^ ^2^, ^3^, ^4^ ^]^ *EWS–FLI1* recruits a set of chromatin and transcriptional regulators, including *BRG1*,^[^ ^5^ ^]^ RNA polymerase II,^[^ ^6^ ^]^ *CREB*‐binding protein (*CBP*)/*p300*,^[^ ^7^ ^]^ RNA helicase A,^[^ ^8^ ^]^ and others, to modulate transcription of target genes, including *NR0B1*, *GLI1*, *FOXOs*, *LOX*, *IGF1*, and others that maintain properties of malignant transformation.^[^ ^9^ ^]^ However, recent studies indicate that *EWS–FLI1* does not act in a binary fashion; rather *EWS–FLI1* expression levels influence cellular states. High levels of *EWS–FLI1* are associated with an immature, proliferative phenotype, whereas reduced levels correlate with decreased proliferation and a more motile cellular phenotype.^[^ ^10^, ^11^ ^]^

As the *EWS–FLI1* fusion occurs exclusively in the tumor cells, it is considered as an ideal target to treat Ewing sarcoma. Prior efforts to identify and target major *EWS–FLI1* downstream genes have not been effective.^[^ ^12^ ^]^ Further, direct targeting *EWS–FLI1* has been hampered by the lack of enzymatic activity and suitable small molecule interaction domains. Notably, a small molecule enantiomer‐specific *EWS–FLI1* inhibitor TK‐216 was identified and being tested in early clinical development.^[^ ^13^ ^]^ Recent efforts aim to block *EWS–FLI1* interaction with DNA^[^ ^14^ ^]^ or modulate its ability to affect chromatin states.^[^ ^15^ ^]^ Targeting *EWS–FLI1* protein stability constitutes a potential therapeutic strategy. Although proteasome‐mediated^[^ ^16^ ^]^ and lysosome‐controlled^[^ ^17^ ^]^ *EWS–FLI1* degradation have been reported, the identities of E3 ligase(s) and deubiquitinase(s) responsible for *EWS–FLI1* protein stability control remain elusive. Ubiquitin carboxyl‐terminal hydrolase 7 (*USP7*) was identified from a CRISPR screen as a dependency for p53‐wild‐type (WT) Ewing sarcoma^[^ ^18^ ^]^ and the deubiquitinase Ubiquitin carboxyl‐terminal hydrolase 19 (*USP19*) was found to stabilize both *EWS–FLI1* and *EWSR1* proteins.^[^ ^19^ ^]^ However, the multiple roles of *USP7* on targeting both tumor suppressors and oncogenes,^[^ ^20^ ^]^ as well as the pleiotropy of *USP19* ^[^ ^21^, ^22^, ^23^, ^24^ ^]^ complicate their applications to treat Ewing sarcoma. Inhibitors of *USP7* ^[^ ^25^, ^26^ ^]^ and *USP19* ^[^ ^27^ ^]^ have been developed, and their effects on Ewing sarcoma remain to be determined.

## 2 Results

### 2.1 The E3 Ligase *SPOP* Targets *EWS–FLI1* for Ubiquitination and Degradation

**Figure 1:**
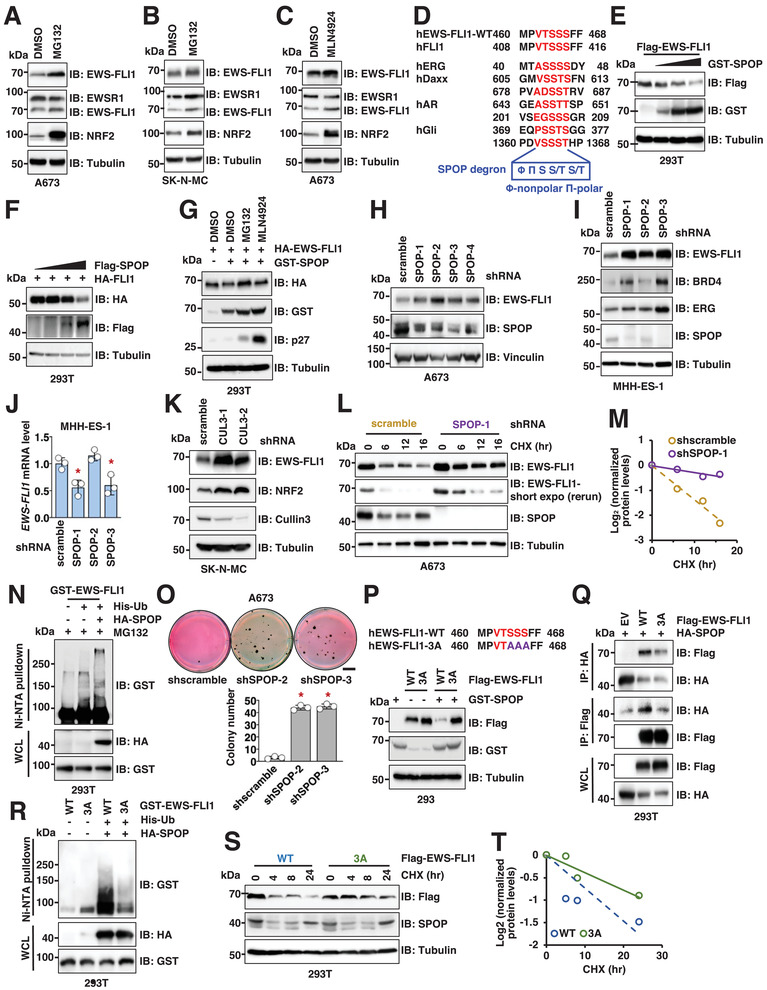
*SPOP* targets *EWS–FLI1* for ubiquitination and degradation depending on a “VTSSS” degron in *EWS–FLI1*. A,B) Immunoblot (IB) analysis of whole cell lysates (WCL) derived from A673 (A) or SK‐N‐MC (B) cells treated with 10 × 10^−6^ m MG132 for 4 h. Cells were lysed in EBC buffer unless specifically noted. Notably, the *EWS–FLI1* signal was detected by either an *EWSR1*‐N antibody (A300‐417) that can detect both *EWSR1* and *EWS–FLI1*, or a *FLI1*‐C antibody (ab180902) that can detect both *FLI1* and *EWS–FLI1*. C) IB analysis of WCL derived from A673 cells treated with 1 × 10^−6^ m MLN4924 overnight. D) Sequence alignment of indicated *EWS–FLI1* species with canonical *SPOP* substrates. E,F) IB analysis of WCL derived from HEK293T cells transfected with indicated DNA constructs. 100 ng Flag‐*EWS–FLI1* or Flag‐*FLI1* construct, together with increasing amounts of *GST*‐*SPOP* (0, 0.5, 1, 2 µg) or Flag‐*SPOP* (0, 1, 2, 4 µg) were transfected into cells. Notably, same amounts of DNA were transfected in each reaction and the differences in DNA amounts were supplemented with pCDNA3.0. Cells were collected 48 h post‐transfection unless specified. G) IB analysis of WCL derived from HEK293T cells transfected with indicated DNA constructs. Where indicated, cells were treated with 10 × 10^−6^ m MG132 or 1 × 10^−6^ m MLN4924 overnight before cell collection. H,I) IB analysis of WCL derived from A673 (H) or MHH‐ES‐1 (I) cells depleted of endogenous *SPOP*. Cells were infected with lentiviruses targeting indicated targets and selected with 1 µg mL^−1^ puromycin for 3 days to eliminate noninfected cells. J) RT‐PCR analyses of *EWS–FLI1* mRNA levels in indicated MHH‐ES‐1 cells. Error bars were calculated as mean +/− SD, *n* = 3. *p < 0.05 (one‐way ANOVA test). K) IB analysis of WCL derived from SK‐N‐MC cells depleted of endogenous *CUL3*. Cells were infected with lentiviruses targeting *cullin 3* and selected with 1 µg mL^−1^ puromycin for 3 days to eliminate non‐infected cells. L,M) IB analysis of WCL derived from control or *SPOP*‐depleted MHH‐ES‐1 cells. Where indicated, 200 µg mL^−1^ cycloheximide (CHX) was added to cell culture and cells were harvested at indicated time periods post CHX addition. (M) is a quantification of (L). N) IB analysis of nickel‐nitrilotriacetic acid (Ni–NTA) pulldowns and WCL derived from HEK293T cells transfected with indicated DNA constructs. Cells were treated with 10 × 10^−6^ m MG132 overnight before cell collection. O) Representative images and quantifications for 3D soft agar assays using indicated cells. Colonies were stained 40 days postinoculation. Error bars were calculated as mean +/− SD, *n* = 3. *p < 0.05 (one‐way ANOVA test). The scale bar represents 5 mm. P) IB analysis of WCL derived from HEK293 cells transfected with 100 ng Flag‐*EWS–FLI1*‐WT or ‐*3A* together with 2 µg *GST*‐*SPOP* constructs. Q) IB analysis of HA or Flag‐IPs and WCL derived from HEK293T cells transfected with indicated DNA constructs. R) IB analysis of Ni–NTA pulldowns and WCL derived from HEK293T cells transfected with indicated DNA constructs. Cells were treated with 10 × 10^−6^ m MG132 overnight before cell collection. S,T) IB analysis of WCL derived from HEK293T cells transfected with indicated Flag‐*EWS–FLI1* constructs. Where indicated, 200 µg mL^−1^ CHX was added to cell culture and cells were harvested at indicated time periods post CHX addition. (T) is a quantification of (S).

We found that blocking the 26S proteasome by MG132 significantly increased the protein abundance of *EWS–FLI1*, but not the wild‐type *EWSR1*, in two Ewing sarcoma cell lines (A673 and SK‐N‐MC) (**Figure** **1**A,B and Figure S1A,B (Supporting Information)), demonstrating that *EWS–FLI1* levels are regulated through protein stability. Inhibition of *cullin* (*CUL*) neddylation by MLN4924 also largely stabilized *EWS–FLI1* but not *EWSR1* in A673 cells (Figure 1C and Figure S1C (Supporting Information)). Antibodies used to detect endogenous *EWSR1*, *FLI1*, and *EWS–FLI1* fusion in Ewing sarcoma cells were validated by a short hairpin RNA (shRNA) against *FLI1*‐C‐terminus in A673 cells (Figure S1D, Supporting Information). These data suggest that in Ewing sarcoma *EWS–FLI1* protein stability is governed by *CUL*‐Ring E3 ligases and that the major degron resides in the *FLI1*‐domain retained in the fusion. By examining *EWS–FLI1* binding to a family of *CULs*, we found *EWS–FLI1* associated with *CUL3*, *CUL4A*, and *CUL5* (Figure S1E, Supporting Information). Examining the sequence of the retained *FLI1* segment, we identified a putative degron sequence (VTSSS) for *SPOP*, a *CUL3* family of E3 ligase. The sequence was located between the E26 transformation‐specific, E‐twenty‐six or Erythroblast transformation specific (ETS) DNA‐binding domain and the carboxyl terminus (Figure 1D). Ectopic expression of *SPOP* promoted *EWS–FLI1* protein degradation in cells in a *SPOP*‐dose‐dependent manner (Figure 1E and Figure S1F (Supporting Information)). Consistent with the presence of the *SPOP* degron in *FLI1*, *SPOP* also destabilized wild‐type *FLI1* but not wild‐type *EWSR1* (Figure 1F and Figure S1G (Supporting Information)). In support, *SPOP* bound the fusion protein but not *EWSR1* (Figure S1H, Supporting Information). Decreasing the possibility that indirect transcriptional control mediated differences in *EWS–FLI1* levels, ectopically expressed *EWS–FLI1* was also decreased by *SPOP*, and this effect was blocked by either MG132 or MLN4924 (Figure 1G).

To further confirm *SPOP* as a physiological E3 ligase for *EWS–FLI1*, we depleted endogenous *SPOP* in 4 Ewing sarcoma cell lines (A673: Figure 1H and Figure S1I (Supporting Information); MHH‐ES‐1: Figure 1I; SK‐N‐MC: Figure S1J (Supporting Information); and EWS894: Figure S1K (Supporting Information)). In each, we observed that *SPOP* depletion led to increased *EWS–FLI1* protein abundance. Notably, depletion of *SPOP* did not increase *EWS–FLI1* messenger RNA (mRNA) levels in MHH‐ES‐1 (Figure 1J) nor A673 cells (Figure S1L, Supporting Information), supporting that *SPOP* regulates *EWS–FLI1* largely through a post‐translational mechanism. Depletion of *CUL3*, the *cullin* partner of *SPOP*, also increased *EWS–FLI1* protein levels in SK‐N‐MC cells (Figure 1K). In further support of *SPOP^CUL3^* as a physiological E3 ligase for *EWS–FLI1*, we observed that *SPOP* depletion extended the half‐life of *EWS–FLI1* proteins (Figure 1L,M), and *SPOP* expression enhanced *EWS–FLI1* ubiquitination in cells (Figure 1N and Figure S1M (Supporting Information)). Demonstrating a functional effect in Ewing cells, we observed that, consistent with previous reports,^[^ ^28^ ^]^ depletion of endogenous *EWS–FLI1* retarded A673 cell growth in vitro (Figure S2A–E, Supporting Information), while *SPOP* depletion enhanced clonal proliferation of A673 cells in soft agar, an effect possibly related to increased *EWS–FLI1* expression (Figure 1O).

To test whether the VTSSS sequence in the *FLI1* segment could function as a degron, we mutated each serine to alanine (*S464A*/*S465A*/*S466A*, *3A*‐*EWS–FLI1*). Compared with WT‐*EWS–FLI1*, the *3A* mutant was resistant to *SPOP*‐mediated degradation (Figure 1P), largely due to deficiency of *3A*‐*EWS–FLI1* binding to both exogenous (Figure 1Q) and endogenous *SPOP* (Figure S3A, Supporting Information). *3A*‐*EWS–FLI1* also displayed a resistance to MG132 treatment (Figure S3B, Supporting Information), reduced ubiquitination levels (Figure 1R), and a longer protein half‐life (Figure 1S,T).

As *SPOP* has been characterized as a *bona fide* E3 ligase that governs *BRD4* protein stability in prostate cancer,^[^ ^29^, ^30^, ^31^ ^]^ and *BRD4* cooperates with *EWS–FLI1* to regulate the *EWS–FLI1*‐mediated transcriptional programs in Ewing sarcoma,^[^ ^32^ ^]^ we examined if *BRD4* is involved in *SPOP*‐depletion‐induced Ewing cell growth control. To this end, we found that *SPOP* depletion only moderately increased *BRD4* proteins in MHH‐ES‐1 cells (Figure 1I) but not in other Ewing sarcoma cells (A673: Figure S1I (Supporting Information) and SN‐N‐MC: Figure S1J (Supporting Information)). Moreover, given that *SPOP* depletion did not affect *EWS–FLI1* mRNA abundance (Figure S1L, Supporting Information), and treatment of MHH‐ES‐1 (Figure S3C, Supporting Information) or A673 (Figure S3D, Supporting Information) cells by a *BRD4* inhibitor JQ1 did not affect *EWS–FLI1* protein levels, it seems that the *SPOP*/*BRD4* signaling axis identified in prostate cancer may not regulate *EWS–FLI1* protein stability in Ewing sarcoma.

### 2.2 *Casein Kinase 1* (*CK1*) Phosphorylates and Primes *EWS–FLI1* for *SPOP* Recognition and Degradation

**Figure 2:**
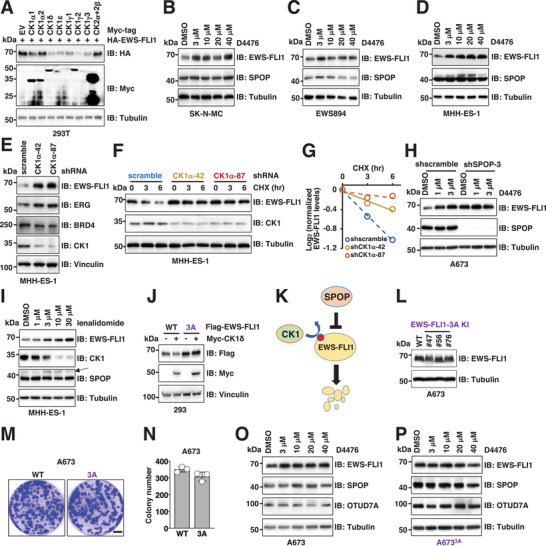
*CK1* phosphorylates and primes *EWS–FLI1* for *SPOP*‐mediated degradation. A) IB analysis of WCL derived from HEK293T cells transfected with indicated DNA constructs. Cells were collected 48 h post‐transfection. B–D) IB analysis of WCL derived from SK‐N‐MC (B), EWS894 (C), and MHH‐ES‐1 (D) cells treated with indicated concentrations of *CK1* inhibitor D4476 for 16 h. E) IB analysis of WCL derived from MHH‐ES‐1 cells depleted of endogenous *CK1α*. Cells were infected with lentiviruses targeting *CK1α* and selected with 1 µg mL^−1^ puromycin for 3 days to eliminate non‐infected cells. F,G) IB analysis of WCL derived from indicated MHH‐ES‐1 cells treated with 200 µg mL^−1^ CHX and harvested at indicated time periods. (G) is a quantification of (F). H) IB analysis of WCL derived from control or endogenous *SPOP*‐depleted A673 cells treated with indicated concentrations of *CK1* inhibitor D4476 for 16 h. I) IB analysis of WCL derived from MHH‐ES‐1 cells treated with indicated concentrations of lenalidomide for 16 h. J) IB analysis of WCL derived from HEK293 cells transfected with 100 ng Flag‐*EWS–FLI1*‐WT or ‐*3A* together with 2 µg *Myc–CK1δ* constructs. K) A cartoon illustration of the proposed model: *CK1*‐mediated *EWS–FLI1* phosphorylation primes *EWS–FLI1* for *SPOP* recognition and degradation. L) IB analysis of WCL derived from parental and three isogenic *EWS–FLI1*‐*S464A*/*S465A*/*S466A* knock‐in A673 cells. M,N) Representative images for 2D colony formation using cells from (L, #76) and quantified in (N). Error bars were calculated as mean +/− SD, *n* = 3. *p < 0.05 (one‐way ANOVA test). Scale bar represents 10 mm. O,P) IB analysis of WCL derived from parental or an isogenic *EWS–FLI1*‐*S464A*/*S465A*/*S466A* knock‐in A673 (L, #76) cells treated with indicated concentrations of *CK1* inhibitor D4476 for 16 h.

Since multiple serines in the “VTSSS” degron could be phosphorylated, and phosphorylation of *SPOP* degrons can enhance *SPOP*–substrate binding,^[^ ^29^, ^33^ ^]^ we next examined whether phosphorylation of the *EWS–FLI1*‐“VTSSS” degron primes *EWS–FLI1* for *SPOP* recognition and degradation. Pursuing the prediction (by GPS3.0) that the serine residues could be phosphorylated by *CKs*, we expressed several distinct *CK1* isoforms and *CK2* kinases and found that most *CK1* isoforms, but not *CK2*, promoted *EWS–FLI1* degradation (**Figure** **2**A). In addition, *CK1* kinase inhibition with D4476 resulted in the accumulation of *EWS–FLI1* in multiple Ewing sarcoma cells (Figure 2B–D and Figure S4A,B (Supporting Information)) without significantly affecting *EWS–FLI1* mRNA levels (Figure S4C–E, Supporting Information). Similar to D4476, genetic depletion of *CK1α* by shRNAs also led to accumulation of endogenous *EWS–FLI1* (Figure 2E), as well as extended *EWS–FLI1* half‐life (Figure 2F,G). Notably, *SPOP* levels were unaffected by D4476, and the effect of D4476 on *EWS–FLI1* levels was attenuated in the context of *SPOP* depletion (Figure 2H). This attenuation was explained by reduced *EWS–FLI1* binding to *SPOP* following D4476 treatment (Figure S4F, Supporting Information). In addition, lenalidomide treatment, a *CK1α* PROTAC that induces *CK1* degradation,^[^ ^34^ ^]^ increased *EWS–FLI1* protein abundance without affecting *EWS–FLI1* mRNA levels (Figure 2I and Figure S4G,H (Supporting Information)). We also found that the *3A* mutant was resistant to *CK1*‐mediated degradation (Figure 2J), supporting Ser464/Ser465/Ser466 as functional *CK1* phosphorylation sites. Cumulatively, these data suggest that *CK1* promotes *SPOP*‐mediated *EWS–FLI1* degradation in a kinase‐activity‐dependent manner (Figure 2K).

Because *3A*‐*EWS–FLI1* was resistant to both *SPOP* (Figure 1P)‐ and *CK1* (Figure 2J)‐mediated degradation, we replaced the degron with the *3A* mutant by CRISPR mediated knock‐in (KI) in A673 cells (A673*^3A^*) (Figure S5A–F, Supporting Information). A673*^3A^* cells expressed comparable levels of *EWS–FLI1* to the parental cells (Figure 2L) and displayed a similar growth in vitro (Figure 2M,N). Increased *EWS–FLI1* protein levels observed in A673 parental cells upon D4476 treatment (Figure 2O) was not observed in the A673*^3A^* KI cells (Figure 2P). In contrast to A673 cells, we observed that neither D4476 treatment (Figure S6A,B, Supporting Information), nor *SPOP* depletion (Figure S6C, Supporting Information) increased *EWS–FLI1* protein abundance in EWS502 cells. Exploring genetic alterations in Ewing sarcoma cells (DEPMAP portal), we noted a point mutation in *CUL3* (*E358Q*) that was present only in EWS502 cells (Figure S6D, Supporting Information). We hypothesized that *E358Q* might result in a loss‐of‐function mutant such that *CUL3*–*E358Q*‐containing *SPOP* E3 ligases in EWS502 cells cannot degrade physiological *SPOP* substrates. Consistent with this notion, *EWS–FLI1* protein levels did not increase following *CUL3* depletion in EWS502 cells (Figure S6E, Supporting Information). Unlike WT‐*CUL3*, ectopic expression of *E358Q–CUL3* failed to promote *EWS–FLI1* degradation (Figure S6F, Supporting Information). Interestingly, as a scaffolding subunit in *CUL3* E3 ligase complexes (Figure S6G, Supporting Information), the *E358Q–CUL3* mutant retained a comparable binding to both *SPOP* (Figure S6H, Supporting Information) and *Rbx1* (Figure S6I, Supporting Information) as WT‐*CUL3*. In addition, *E358Q–CUL3* was also efficiently neddylated to a comparable level as WT‐*CUL3* in cells (Figure S6J, Supporting Information), a modification critical for *CUL3* E3 ligase activation and function.^[^ ^35^ ^]^ These results suggest that the *E358Q–CUL3* mutant forms an intact *SPOP^CUL3^* E3 ligase complex. Notably, compared with WT‐*CUL3*, *E358Q–CUL3* was deficient in facilitating *SPOP* binding to *EWS–FLI1* (Figure S6K, Supporting Information), suggesting that the inability of *SPOP^CUL3–E358Q^* E3 ligase complexes in degrading *EWS–FLI1* might partly be due to that the *E358Q–CUL3* mutation weakens *SPOP* binding to its substrates, including *EWS–FLI1*. This result offers an additional layer of regulation for *SPOP* binding to its substrates through *CUL3* mutations. Together, these data further support a physiological role of *SPOP^CUL3^* in targeting *EWS–FLI1* for degradation and suggest that Ewing sarcoma tumors may inactivate *SPOP*‐mediated *EWS–FLI1* degradation through *CUL3* mutations to promote Ewing sarcoma growth.

### 2.3 The Deubiquitinase OTU domain‐containing protein 7A (*OTUD7A*) is Identified as a Deubiquitinating Enzyme (DUB) to Control *EWS–FLI1* Protein Stability through a Genetic Screen

**Figure 3:**
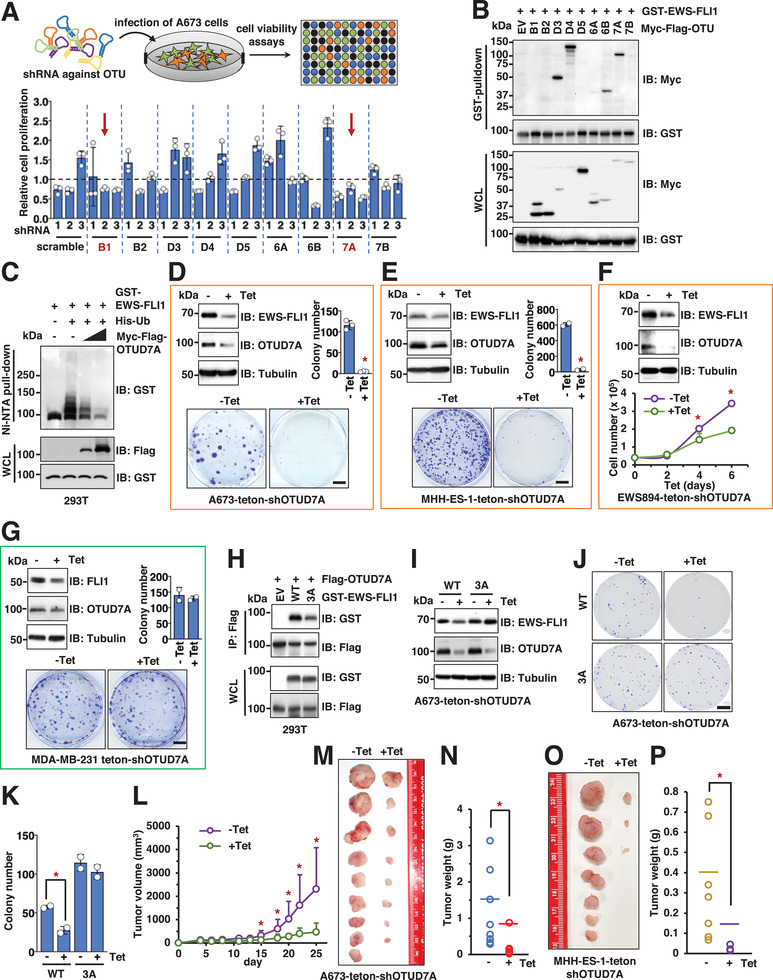
Genetic inactivation of *OTUD7A* leads to decreased *EWS–FLI1* protein abundance and subsequently impeded Ewing sarcoma cell growth in vitro and in mice. A) shRNA‐mediated *OTUB1* and *OTUD7A* depletion attenuates A673 cell viability. Top panel, illustration of the pipeline for shRNA‐guided screen: 3 independent shRNAs targeting each *OTU* were used to deplete endogenous *OTU* targets. 3 days postinfection, 1000 cells were plated into 96‐well plates in triplicates and cell viability was monitored 3 days postseeding by MTT assays. Error bars were calculated as mean +/− SD, *n* = 3. B) IB analysis of *GST*‐pulldown and WCL derived from HEK293T cells transfected with indicated DNA constructs. C) IB analysis of Ni–NTA pulldown and WCL derived from HEK293T cells transfected with indicated DNA constructs. D–G) Top panels, IB analysis of WCL derived from A673 (D), MHH‐ES‐1 (E), EWS894 (F), or MDA‐MB‐231 (G) cells depleted of endogenous *OTUD7A* by a tet‐on shRNA against endogenous *OTUD7A*. 1 µg mL^−1^ tetracycline was added into cell culture for 72 h before cell collection. Bottom panels, representative colony formation assays (D, E, G) or cell growth assays (F) using cells obtained in the corresponding top panels. Error bars were calculated as mean +/− SD, *n* = 3 for (D), (F) and *n* = 2 for (E), (G). *p < 0.05 (one‐way ANOVA test). For (D) and (G), the scale bar represents 5 mm. For (E), the scale bar represents 10 mm. H) IB analysis of Flag‐IPs and WCL derived from HEK293T cells transfected with indicated DNA constructs. I) IB analysis of WCL derived from parental or *EWS–FLI1*‐*3A* knock‐in A673 cells expressing teton‐sh*OTUD7A*. Where indicated, 1 µg mL^−1^ tetracycline was added into cell culture for 72 h before cell collection. J,K) Representative images for 2D colony formation using cells from (I) and quantified in (K). Error bars were calculated as mean +/− SD, *n* = 2. *p < 0.05 (one‐way ANOVA test). The scale bar represents 10 mm. L–N) Mouse xenograft experiments were performed with indicated A673 cells. 5 days postinjection when tumors were established in mice, either tetracycline dissolved in water with 1% sucrose, or 1% sucrose dissolved in water only, was fed to mice. Tumor volumes were monitored by caliper measurements at indicated days (L). 25 days postinjection, mice were sacrificed, and tumors were dissected (M) and weighed (N). Error bars were calculated as mean +/− SD, *n* = 9. *p < 0.05 (one‐way ANOVA test). O,P) Mouse xenograft experiments were performed with indicated MHH‐ES‐1 cells. 7 days postinjection when tumors were established in mice, either tetracycline dissolved in water with 1% sucrose, or 1% sucrose dissolved in water only, was fed to mice. 32 days postinjection, mice were sacrificed, and tumors were dissected (O) and weighed (P). Error bars were calculated as mean +/− SD, *n* = 7. *p < 0.05 (one‐way ANOVA test).

Since *SPOP*/*CK1* destabilizes *EWS–FLI1*, activation of *SPOP*/*CK1* could offer a therapeutic strategy to treat Ewing sarcoma. However, potential tumor suppressor functions of *SPOP* ^[^ ^33^, ^36^ ^]^ and *CK1*,^[^ ^37^ ^]^ as well as the predicted challenge of targeting *SPOP^CUL3^*, led us to evaluate for possible DUBs that would antagonize *SPOP^CUL3^* function to stabilize *EWS–FLI1*. Among the five families of DUBs,^[^ ^38^ ^]^ we focused on *ovarian tumor proteases* (*OTUs*) since they recognize specific ubiquitin chain linkages to regulate distinct signaling cascades associated with human tumors.^[^ ^39^ ^]^ Thus far, 16 mammalian *OTUs* have been identified. We and others have reported roles of *OTUD7B* in maintaining mechanistic target of rapamycin (*mTOR*) complex homeostasis,^[^ ^40^ ^]^ activating NF‐*κ*B signaling^[^ ^41^ ^]^ and regulating the cell cycle.^[^ ^42^ ^]^ However, the physiological roles for the majority of *OTUs* are just beginning to be appreciated. Since *EWS–FLI1* is necessary for Ewing sarcoma growth,^[^ ^43^ ^]^ we reasoned that inhibiting DUBs that stabilize *EWS–FLI1* would reduce Ewing sarcoma cell proliferation by downregulating *EWS–FLI1*. We screened *OTU*‐directed shRNAs for those that decreased A673 cell proliferation. Three independent shRNAs were used to silence each of 9 *OTU* genes. Cell viability was monitored at 3 days post‐shRNA infection by 3‐(4,5‐dimethylthiazol‐2‐yl)‐2,5‐diphenyltetrazolium bromide (MTT) assays, or at 3‐week after shRNA infection by colony formation assays. We found that depletion of Ubiquitin thioesterase OTUB1 (*OTUB1*) and *OTUD7A* reduced A673 cell growth (**Figure** **3**A) and diminished colony formation (Figure S7A,B, Supporting Information). Because alterations in cell growth could also result from non‐*EWS–FLI1 OTU* targets, we next examined interactions between each individual *OTU* with *EWS–FLI1*. We found that *OTUD3*, *OTUD4*, *OTUD6B*, and *OTUD7A* bound to *EWS–FLI1* in cells (Figure 3B). Among these 4 *OTUs*, ectopic expression of *OTUD3* or *OTUD7A* (Figure S8A,B, Supporting Information), but not *OTUD6B* nor *OTUD4* (Figure S8B,C, Supporting Information), stabilized endogenous *EWS–FLI1* proteins in A673 cells. These data support *OTUD3* and *OTUD7A* as candidates to regulate *EWS–FLI1* protein stability. Consistently, both *OTUD3* and *OTUD7A* could deubiquitinate *EWS–FLI1* in cells (Figure 3C and Figure S8D,E (Supporting Information)). However, in MHH‐ES‐1 cells, depletion of endogenous *OTUD3* minimally influenced *EWS–FLI1* protein abundance (Figure S8F, Supporting Information) but significantly affected cell growth (Figure S8G,H, Supporting Information). This suggests that sh*OTUD3*‐induced growth reduction may be *EWS–FLI1*‐independent. Depletion of endogenous *OTUD7A* by shRNAs (Figure S8I, Supporting Information) led to reduced *EWS–FLI1* protein abundance in SK‐N‐MC cells. Moreover, an interaction of OTUD7A with EWS–FLI1 at endogenous levels was observed (Figure S8J, Supporting Information). These data support *OTUD7A* as a possible *EWS–FLI1* deubiquitinating enzyme to control *EWS–FLI1* protein stability.

### 2.4 Genetic Depletion of *OTUD7A* Impedes Ewing Sarcoma Growth

Stable *OTUD7A* depletion led to cell death within a week of shRNA or sgRNA infection, preventing us from further analyzing the signaling changes and biological effects of *OTUD7A* loss. To overcome this, we developed an inducible *OTUD7A* depletion system. 48 h post‐tetracycline (Tet) addition, we observed a reduction in endogenous *OTUD7A* and *EWS–FLI1* proteins in A673 cells (Figure S9A, Supporting Information), with minimal effects on *EWS–FLI1* mRNA (Figure S9B, Supporting Information). Remarkably, induced depletion of *OTUD7A* led to reduced *EWS–FLI1* protein levels in multiple Ewing sarcoma cells, including A673 (Figure 3D), MHH‐ES‐1 (Figure 3E), and EWS894 (Figure 3F). MG132 treatment largely preserved *EWS–FLI1* protein levels following *OTUD7A* depletion (Figure S9C–E, Supporting Information), further supporting a role of *OTUD7A* in regulating *EWS–FLI1* protein stability. Importantly, for all Ewing sarcoma cell lines tested, *OTUD7A* depletion reduced cell proliferation in vitro (Figure 3D–F). By contrast, depletion of endogenous *OTUD7A* in non‐Ewing sarcoma cells, such as MDA‐MB‐231 cells, by either tet‐inducible sh*OTUD7A* (Figure 3G) or stable *OTUD7A* depletion (Figure S9F,G, Supporting Information) did not significantly affect cell growth in vitro, although it reduced endogenous *FLI1* protein abundance (Figure 3G and Figure S9H (Supporting Information)).

In further support of a role for *OTUD7A* in *EWS–FLI1* regulation, we observed an interaction of *OTUD7A* with *EWS–FLI1* at endogenous levels (Figure S8J, Supporting Information). The ubiquitination‐deficient *3A*‐*EWS–FLI1* demonstrated reduced binding ability with *OTUD7A* (Figure 3H), and depletion of *OTUD7A* failed to reduce *3A*‐*EWS–FLI1* protein levels (Figure 3I). These data suggest that *OTUD7A* stabilizes *EWS–FLI1* proteins through the *EWS–FLI1*‐“VTSSS” motif or *EWS–FLI1* ubiquitination. Thus, A673*^3A^* cells offered a model to examine specific effects of inactivating the *OTUD7A*/*EWS–FLI1* signaling. In further support of the inactivation of *OTUD7A* impeding Ewing sarcoma proliferation, depletion of *OTUD7A* resulted in significantly reduced colony formation in vitro in A673^WT^ but not A673*^3A^* cells (Figure 3J,K). Moreover, depletion of *OTUD7A* dramatically reduced tumor growth (Figure 3L) and tumor formation of A673 (Figure 3M,N), but not A673*^3A^* (Figure S10A–C, Supporting Information) cells grown as xenografts. Depletion of *OTUD7A* also retarded xenografted MHH‐ES‐1 tumor development (Figure 3O,P and Figure S10D,E (Supporting Information)). Further histological analyses of xenografted MHH‐ES‐1 tumors revealed that induced depletion of *OTUD7A* efficiently reduced *EWS–FLI1* protein levels and subsequent cell proliferation (evidenced by Ki67 staining), accompanied by increased cell death (cleaved‐*caspase 3*) (Figure S10F, Supporting Information). Together, these data demonstrate the dependence of Ewing sarcoma growth in vitro and in a xenografted mouse model on *OTUD7A*.

Although it is known that *FLI1* cannot rescue the loss of *EWS–FLI1* in Ewing sarcoma, we detected *3A* mutation in both *EWS–FLI1* and *FLI1* alleles in A673 cells (Figure S5, Supporting Information). To formally demonstrate that the effect of *OTUD7A* is through the fusion oncoprotein, we expressed *EWS–FLI1*‐*3A* by lentiviral infection in A673‐teton‐sh*OTUD7A* cells (Figure S11A, Supporting Information) and observed that *EWS–FLI1*‐*3A* was resistant to *OTUD7A* depletion (Figure S11B, Supporting Information). As a result, unlike WT‐*EWS–FLI1*, *OTUD7A* depletion failed to significantly impede *3A*‐*EWS–FLI1* expressing A673 cell growth in vitro (Figure S11C,D, Supporting Information) and as a xenograft (Figure S11E,F, Supporting Information). As predicted, reconstitution of *FLI1*‐*3A* expression in A673‐teton‐sh*OTUD7A* cells (Figure S11G,H, Supporting Information) could not rescue *OTUD7A*‐depletion‐induced A673 cell growth retardation in vitro (Figure S11I–K, Supporting Information). These data suggest that *OTUD7A* controls A673 cell growth largely through regulating *EWS–FLI1* but not *FLI1* protein stability. To further reinforce this notion, we also expressed *EWS–FLI1*‐*3A* in both EWS894‐teton‐sh*OTUD7A* (Figure S11L,M, Supporting Information) and MHH‐ES‐1‐teton‐sh*OTUD7A* cells (Figure S11O,P, Supporting Information) and found that *EWS–FLI1*‐*3A* largely rescued *OTUD7A*‐depletion‐induced growth retardation in both cell lines (Figure S11N,Q, Supporting Information). Together, these data support that *OTUD7A* largely governs Ewing sarcoma growth by maintaining *EWS–FLI1* protein stability and support prior studies demonstrating that *EWS–FLI1* acts distinctly from *FLI1*.

### 2.5 Quantitative Proteomics Supports *EWS–FLI1* as an Endogenous *OTUD7A* Target and Defines a Subset of Characterized *EWS–FLI1* Downstream Targets Mediating *OTUD7A‐*/*EWS–FLI1‐*Governed Cell Growth

**Figure 4:**
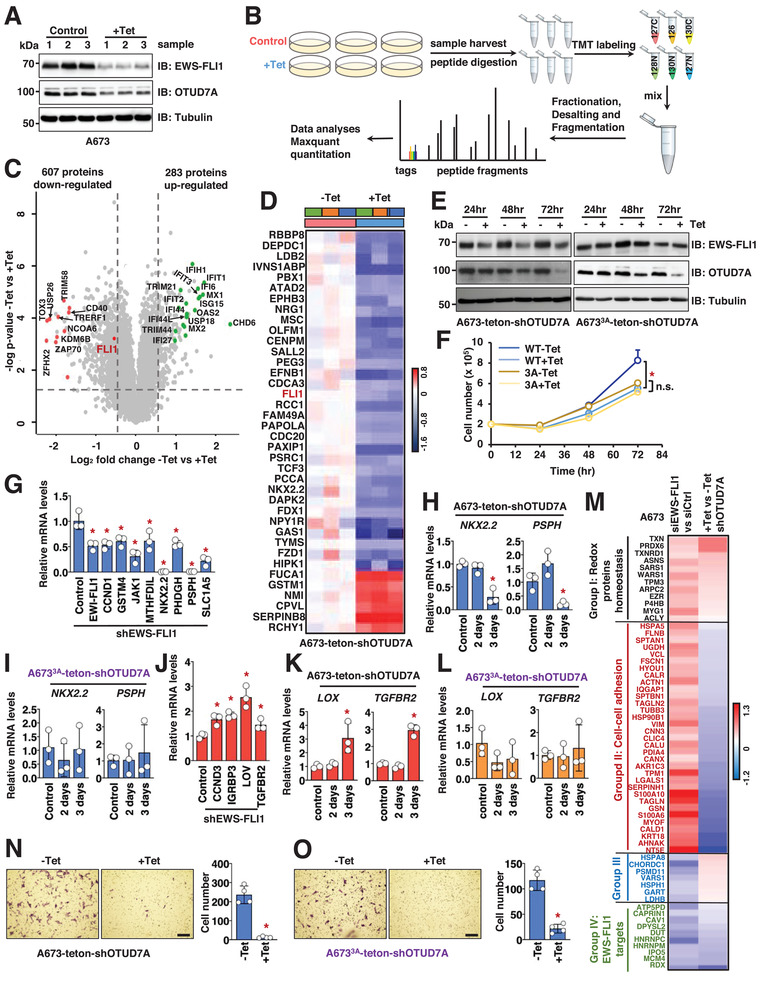
Genetic inactivation of *OTUD7A* suppresses key *EWS–FLI1* downstream signaling. A) IB analysis of WCL derived from A673 cells treated with Tet (tetracycline, 1 µg mL^−1^) for 72 h. B) A cartoon illustration of the working pipeline for TMT labeling and quantitative mass spectrometry analyses. C) A volcano plot showing down‐ and upregulated genes. *EWS–FLI1* is indicated in red color. D) A heatmap summarizing the statistically significantly changed characterized *EWS–FLI1* targets in control and *OTUD7A*‐depleted A673 cells. E) IB analyses of WCL derived from WT or *3A*‐A673 cells infected with Tet‐inducible sh*OTUD7A* constructs. Where indicated, cells were collected upon treatment with 1 µg mL^−1^ tetracycline (Tet) for indicated periods before cell collection. F) A growth curve for cells indicated in (G) for indicated time periods measured by cell number. Error bars were calculated as mean +/− SD, *n* = 3. *p < 0.05 (one‐way ANOVA test). G,J) RT‐PCR analyses of indicated gene changes in control and *EWS–FLI1*‐depleted A673 cells. Lentiviruses coding sh*EWS–FLI1* were used to infect A673 cells and selected with 1 µg mL^−1^ puromycin to eliminate noninfected cells for 72 h before mRNA extraction. Error bars were calculated as mean +/− SD, *n* = 3. *p < 0.05 (one‐way ANOVA test). H,I,K,L) RT‐PCR analyses of mRNAs derived from indicated cells treated with 1 µg mL^−1^ Tet for indicated periods before cell collection. Error bars were calculated as mean +/− SD, *n* = 3. *p < 0.05 (one‐way ANOVA test). M) A heatmap summarizing the statistically significantly changed proteins upon *OTUD7A* depletion in A673 cells in (A) that are overlapped with a previous proteomic study^[^ ^45^ ^]^ identifying protein changes upon *EWS–FLI1* depletion in A673 cells. Group I: common hits from our study and the previous study^[^ ^45^ ^]^ that show protein abundance increases upon either *EWS–FLI1* or *OTUD7A* depletion; Group II: cell–cell adhesion proteins showed decreased expression upon *OTUD7A* depletion but increased expression upon *EWS–FLI1* depletion; Group III: proteins showed decreased expression upon *EWS–FLI1* depletion but increased expression upon *OTUD7A* depletion; Group IV: common hits from our study and the previous study^[^ ^45^ ^]^ that show protein abundance decreases upon either *EWS–FLI1* or *OTUD7A* depletion. N,O) Representative images for in vitro transwell assays using indicated WT (N) or *EWS–FLI1‐3A* knock‐in (O) A673‐teton‐sh*OTUD7A* cells treated with 1 µg mL^−1^ tetracycline for 72 h before cell fixation and staining. Error bars were calculated as mean +/− SD, *n* = 4. *p < 0.05 (one‐way ANOVA test). The scale bar represents 50 µm.

To further understand the pathophysiological function of *OTUD7A* in Ewing sarcoma, we performed a quantitative proteomics study following genetic *OTUD7A* inactivation in A673 cells. 72 h following *OTUD7A* depletion by shRNA induction, we observed significantly reduced *EWS–FLI1* protein levels (**Figure** **4**A and Figure S12A (Supporting Information)). At this time, proteins extracted from *OTUD7A*‐depleted (or control) cells were subjected to nonbiased quantitative mass spectrometry analyses to determine differences in protein abundance (Figure 4B). After excluding common contaminants and proteins nonspecifically enriched from reported microproteins, a total of 7641 nonredundant proteins were identified with protein abundance changes (Table S1, Supporting Information). These data constitute one of the largest Ewing sarcoma‐related proteomic datasets to date. Applying a *p*‐value < 0.05 and log_2_ fold change > 0.5 or <‐0.5 threshold for differential protein abundance, we observed that *OTUD7A* depletion resulted in statistically significant changes of abundance for 890 endogenous proteins, with 283 being upregulated and 607 downregulated (Figure 4C). Notably, our proteomic data were highly reproducible among replicates within the same group (Figure S12B,C, Supporting Information). We found that *FLI1* C‐terminus peptides (derived from *EWS–FLI1*) were significantly decreased (Figure 4C and **Table** **1**), a result consistent with our western blot analyses (Figure 4A). To explore whether *OTUD7A* depletion may modulate the abundance of proteins encoded by *EWS–FLI1* transcriptional targets, we compared our proteomic results with a well‐developed transcriptomic study that identified 503 *EWS–FLI1* transcriptional targets.^[^ ^44^ ^]^ We found that 201 proteins were identified in both our proteomics study and the transcriptomic study. Among them, 33 proteins were significantly downregulated (including *EWS–FLI1*, Figure 4D and Table 1) and 6 proteins were significantly upregulated (**Table** **2**) upon genetic *OTUD7A* depletion. Another 99 *EWS–FLI1* transcriptional targets^[^ ^44^ ^]^ demonstrated decreased levels but did not reach statistical significance (*p* < 0.05) (Table S2, Supporting Information). These data suggest that *OTUD7A*/*EWS–FLI1* signaling modulates a subset of *EWS–FLI1* targets.

**Table 1:** A list of 33 defined *EWS–FLI1* downstream target protein abundance reduced by *OTUD7A* depletion in A673 cells

Gene	log2 ‐Tet vs +Tet fold change
*SRSF protein kinase 1 (SRPK1)*	−0.5
*cell division cycle‐associated protein 3 (CDCA3)*	−0.52
*cell division cycle protein 20 homolog (CDC20)*	−0.52
*thymidylate synthase (TYMS)*	−0.52
*paternally‐expressed gene 3 protein (PEG3)*	−0.53
*transcription factor E2‐alpha (TCF3)*	−0.55
*adrenodoxin, mitochondrial (FDX1)*	−0.56
*CYFIP‐related Rac1 interactor A (FAM49A)*	−0.56
*Friend leukemia integration 1 transcription factor (FLI1)*	−0.57
*ATPase family AAA domain‐containing protein 2 (ATAD2)*	−0.57
*proline/serine‐rich coiled‐coil protein 1 (PSRC1)*	−0.59
*pre‐B‐cell leukemia transcription factor 1 (PBX1)*	−0.6
*poly(A) polymerase alpha (PAPOLA)*	−0.61
*pro‐neuregulin‐1, membrane‐bound isoform (NRG1)*	−0.62
*homeobox protein Nkx‐2.2 (NKX2.2)*	−0.67
*ephrin type‐B receptor 3 (EPHB3)*	−0.68
*propionyl‐CoA carboxylase alpha chain, mitochondrial (PCCA)*	−0.73
*sal‐like protein 2 (SALL2)*	−0.7
*noelin (OLFM1)*	−0.76
*death‐associated protein kinase 2 (DAPK2)*	−0.7
*DNA endonuclease RBBP8 (RBBP8)*	−0.79
*frizzled‐1 (FZD1)*	−0.8
*centromere protein M (CENPM)*	−0.81
*regulator of chromosome condensation (RCC1)*	−0.84
*DEP domain‐containing protein 1A (DEPDC1)*	−0.86
*LIM domain‐binding protein 2 (LDB2)*	−0.88
*PAX‐interacting protein 1 (PAXIP1)*	−1.08
*musculin (MSC)*	−1.09
*neuropeptide Y receptor type 1 (NPY1R)*	−1.1
*ephrin‐B1 (EFNB1)*	−1.1
*homeodomain‐interacting protein kinase 1 (HIPK1)*	−1.11
*influenza virus NS1A‐binding protein (IVNS1ABP)*	−1.24
*growth arrest‐specific protein 1 (GAS1)*	−1.56

**Table 2:** A list of 6 defined *EWS–FLI1* downstream targets with protein abundance increased by *OTUD7A* depletion in A673 cells

Gene	log2 ‐Tet vs +Tet fold change
*serpin B8 (SERPINB8)*	0.72
*tissue alpha‐L‐fucosidase (FUCA1)*	0.64
*N‐myc‐interactor (NMI)*	0.60
*probable serine carboxypeptidase CPVL (CPVL)*	0.56
*glutathione S‐transferase Mu 1 (GSTM1)*	0.52
*RING finger and CHY zinc finger domain‐containing protein 1 (RCHY1)*	0.51

In addition to characterized *EWS–FLI1* target proteins whose protein abundance was controlled by *OTUD7A* (Figure 4D), there were additional 572 proteins downregulated by *OTUD7A* genetic depletion (Table S3, Supporting Information), suggesting they are potential targets for *OTUD7A* or uncharacterized *EWS–FLI1* targets. Further DAVID analyses led to identification of enriched biological functions for these hits by plotting the −log *p*‐value against log_2_ enrichment (**Tables** 2 and **3** and Figure S12D (Supporting Information)). Consistent with *EWS–FLI1* signaling being a major *OTUD7A* downstream effector, more than one tenth (62) of the downregulated proteins exert DNA‐binding transcriptional activity (Figure S12D, Supporting Information), many of which have been characterized to associate with *EWS–FLI1* on chromatin, including *CBP*, forkhead box proteins, and zinc finger proteins.

**Table 3:** Top enriched functions for downregulated and upregulated proteins in *OTUD7A*‐depleted A673 cells

Function	Count	*p*‐value	Fold enrichment
Disulfide bond	114	3.01 × 10^−25^	2.77
Transcription factor activity, sequence‐specific DNA binding	62	1.93 × 10^−10^	2.36
Homeobox	21	7.90 × 10^−10^	5.09
Immunoglobulin‐like fold	35	1.51 × 10^−9^	3.17
Extracellular matrix organization	25	1.83 × 10^−9^	4.13
Epidermal‐growth‐factor‐like domain	17	4.44 × 10^−7^	4.43
Protein digestion and absorption	11	1.32 × 10^−6^	6.72
Insulin‐like growth factor binding protein, N‐terminal	13	3.04 × 10^−6^	5.08
High mobility group (HMG) box domain	13	8.98 × 10^−6^	4.65
Integrin complex	6	3.10 × 10^−4^	8.31
Antiviral defense	29	8.83 × 10^−23^	11.31
Type I interferon signaling pathway	21	5.96 × 10^−20^	15.66
Innate immunity	25	1.68 × 10^−13^	6.6
Response to virus	16	3.93 × 10^−9^	7.01
2ʹ‐5ʹ‐Oligoadenylate synthetase activity	4	1.69 × 10^−4^	28.2
retinoic acid‐inducible gene I (*RIG‐I*)‐like receptor signaling pathway	8	9.57 × 10^−4^	4.88
Thiol protease	11	0.001573	3.33
Response to cytokine	8	0.003901	3.92
Mitophagy	6	0.004002	5.52
Nucleophagy	16	0.007451	2.14

### 2.6 Genetic *OTUD7A* Inactivation Reduces Expression of *EWS–FLI1* Transcriptional Targets

To further confirm that the decreased protein levels for a subset of characterized *EWS–FLI1* transcription targets following *OTUD7A* depletion (Figure 4D) were regulated through the *OTUD7A*/*EWS–FLI1* signaling, we examined mRNA abundance. Reduced *EWS–FLI1* protein was observed 2 days following *OTUD7A* shRNA induction (Figure 4E and Figure S12E,F (Supporting Information)). No significant cell growth changes were observed 2 days post‐Tet induction (Figure 4F and Figure S12G (Supporting Information)). Differences in cell proliferation were detected 3 days following *OTUD7A* depletion, one day following the decrease in *EWS–FLI1* (Figure S12H,I, Supporting Information). We hypothesize that this difference results from a lag in the downregulation of *EWS–FLI1* targets. To test this possibility, we extracted mRNAs from both A673^WT^‐teton‐sh*OTUD7A* and A673*^3A^*‐teton‐sh*OTUD7A* cells 2 and 3 days following tetracycline addition. A673 cells depleted of endogenous *EWS–FLI1* by shRNAs served as a control. *EWS–FLI1* depletion led to reduced *EWS–FLI1* mRNA levels, as well as downregulation of known *EWS–FLI1* target genes, with homeobox protein Nkx‐2.2 (*NKX2.2*) and phosphoserine phosphatase (*PSPH*) being the most significantly affected targets (Figure 4G). 3 days of treatment resulted in greater suppression of these targets (Figure 4H and Figure S12J–M (Supporting Information)), an effect not detected in A673*^3A^*‐teton‐sh*OTUD7A* cells (Figure 4I). *EWS–FLI1* depletion also increased expression of targets negatively regulated by *EWS–FLI1* including lysyl oxidase (*LOX*) and TGF‐beta receptor type‐2 (*TGFBR2*) (Figure 4J). *OTUD7A* depletion for 3 days, but not 2 days, led to significantly increased *LOX*, *TGFBR*, and other *EWS–FLI1* transcripts in A673^WT^‐teton‐sh*OTUD7A* (Figure 4K and Figure S12N–P (Supporting Information)) but not A673*^3A^*‐teton‐sh*OTUD7A* cells (Figure 4L). These data support that changes in *EWS–FLI1* following *OTUD7A* depletion affect a subset of *EWS–FLI1* transcriptional targets.

### 2.7 Quantitative Proteomics Identifies *OTUD7A* Downstream Targets Mediating Ewing Sarcoma Cell Migration

We compared our proteomic results with a previous study that identified protein abundance changes following *EWS–FLI1* silencing.^[^ ^45^ ^]^ Our analysis identified 103 out of 105 differentially expressed proteins controlled by *EWS–FLI1* in the previous study,^[^ ^45^ ^]^ among which 65 were significantly changed upon *OTUD7A* depletion (Figure 4M). Of the 33 proteins upregulated by si*EWS–FLI1* but downregulated by *OTUD7A* depletion, 12 were associated with cell–cell adhesion (Group II in Figure 4M). Consistent with previous reports showing *EWS–FLI1* depletion reduces proliferation but enhances motility,^[^ ^10^ ^]^ we found that *EWS–FLI1* depletion increased A673 cell migration in vitro (Figure S13A,B, Supporting Information). Intriguingly, *OTUD7A* depletion significantly reduced cell migration in vitro, in both A673^WT^ and A673*^3A^* cells (Figure 4N,O and Figure S13C,D (Supporting Information)). This result is consistent with the reduced expression of cell–cell adhesion proteins. These data suggest that *OTUD7A* influences Ewing sarcoma migration independent of *EWS–FLI1*. Protein candidates related to cell migration that were decreased upon depletion of *OTUD7A* but increased upon *EWS–FLI1* depletion included integrins (Figure S13E,F, Supporting Information) and collagens (Figure S13G,H, Supporting Information) such as *ITGAV* and *COL3A1*. Depletion of *EWS–FLI1* increased expression of integrin alpha‐V (*ITGAV*) and collagen alpha‐1(III) chain (*COL3A1*) (Figure S13I, Supporting Information), whereas *OTUD7A* depletion significantly reduced levels of both proteins (Figure S13J, Supporting Information). Interestingly, putative *SPOP* degrons were identified in *ITGAV* and *COL3A1*, suggesting that, similar to *EWS–FLI1*, *OTUD7A* may cooperate with *SPOP* to regulate these proteins (Figure S13K, Supporting Information). These data cumulatively suggest that *OTUD7A* inactivation not only impedes Ewing cell growth largely through reduced *EWS–FLI1* protein stability, but also inhibits Ewing sarcoma motility through an *EWS–FLI1*‐independent manner, possibly by regulating the levels of cell–cell‐adhesion‐related proteins (Figure S13L, Supporting Information). These results motivated us to search for potential small molecules that would inhibit *OTUD7A* catalytic activity as a possible therapeutic strategy for Ewing sarcoma.

### 2.8 *OTUD7A* is Expressed across Tissues, Including Ewing Sarcoma Tumors

**Figure 5:**
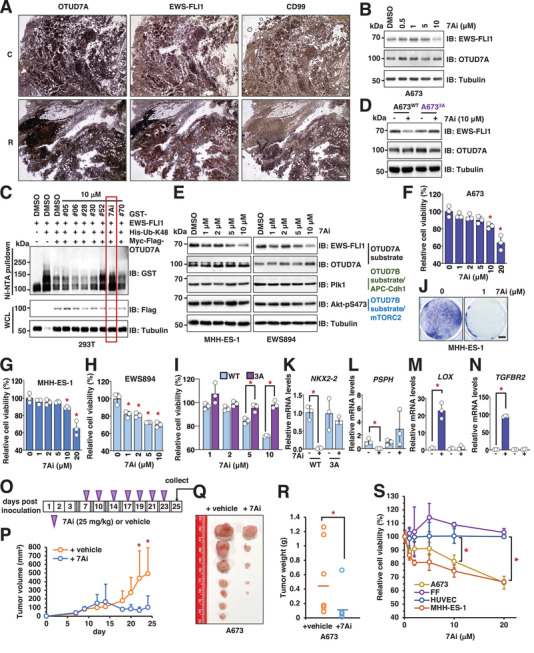
Identification of 7Ai as a lead compound to inhibit *OTUD7A* activation to suppress Ewing sarcoma growth. A) Representative IHC images for two Ewing sarcoma tumors obtained from patients stained with indicated antibodies. The scale bar represents 25 µm. C: calvarium; R: rib. B) IB analysis of WCL derived from A673 cells treated with indicated doses of compound 7Ai for 12 h before cell collection. C) IB analysis of Ni–NTA pulldowns and WCL derived from HEK293T cells transfected with indicated DNA constructs. Where indicated, indicated compounds were added to cell culture 10 h prior to cell collection. D) IB analysis of WCL derived from indicated A673 cells treated with 10 × 10^−6^ m compound 7Ai for 12 h before cell collection. E) IB analysis of WCL derived from MHH‐ES‐1 or EWS894 cells treated with indicated doses of compound 7Ai for 12 h before cell collection. F–H) Representative cell viability assays using A673 (F), MHH‐ES‐1 (G), and EWS894 (H) cells treated with indicated doses of compound 7Ai for 72 h before measurements. Error bars were calculated as mean +/− SD, *n* = 3. *p < 0.05 (one‐way ANOVA test). I) Representative cell viability assays using A673^WT^ or A673*^3A^* cells treated with indicated doses of compound 7Ai for 72 h before measurements. Error bars were calculated as mean +/− SD, *n* = 3. *p < 0.05 (one‐way ANOVA test). J) Representative images for 2D colony formation by MHH‐ES‐1 cells treated with indicated doses of compound 7Ai for 14 days. The scale bar represents 10 mm. K–N) RT‐PCR analyses of mRNA level changes of characterized *EWS–FLI1* downstream target genes in both WT and *EWS–FLI1‐3A* knock‐in A673 cells treated with 1 µg mL^−1^ Tet for 3 days including *NKX2‐2* (K), *PSPH* (L), *LOX* (M), and *TGFBR2* (N). Error bars were calculated as mean +/− SD, *n* = 3. *p < 0.05 (one‐way ANOVA test). O) An illustration of the timeline for 7Ai administration into mice. At indicated periods, 25 mg kg^−1^ 7Ai was supplied through IP injection into each mouse. P–R) Mouse xenograft experiments were performed with A673 cells treated with vehicle or 7Ai. 7 days postinjection when tumors were established in mice, 7Ai (25 mg kg^−1^) was injected through IP route to mice. Tumor volumes were monitored by caliper measurements at indicated days (P). 25 days postinjection, mice were sacrificed and tumors were dissected (Q) and weighed (R). Error bars were calculated as mean +/− SD, *n* = 7. *p < 0.05 (one‐way ANOVA test). S) Representative cell viability assays using two Ewing sarcoma cells (A673 and MHH‐ES‐1) and two normal control cells (HUVEC and foreskin fibroblast (FF)) treated with indicated doses of compound 7Ai for 72 h before measurements. Error bars were calculated as mean +/− SD, *n* = 3. *p < 0.05 (one‐way ANOVA test).

We next examined the therapeutic potential of inhibiting *OTUD7A* in treating Ewing sarcoma. We first demonstrated that, in contrast to WT, the catalytic‐dead *C210S–OTUD7A* did not stabilize *EWS–FLI1* (Figure S14A, Supporting Information). This result confirmed dependence on the *OTUD7A* deubiquitinase activity in regulating *EWS–FLI1* protein stability. We next profiled expression of *OTUD7A* proteins in a panel of commonly used Ewing sarcoma cell lines commonly used in labs and identified that all Ewing sarcoma cells expressed detectable *OTUD7A* (Figure S14B, Supporting Information). Evaluation of transcriptomic data for Ewing sarcoma cell lines and Ewing sarcoma tumors^[^ ^46^ ^]^ also revealed levels of *OTUD7A* mRNA expression (Figure S14C, Supporting Information). Following validation of an *OTUD7A* antibody (Figure S14D, Supporting Information), we assayed a human normal tissue microarray (TMA) and observed varied expression levels of *OTUD7A* among tissues (Figure S14E, Supporting Information), largely consistent with immunohistochemistry (IHC) staining of tissues provided by Human Protein Atlas. In addition, expression of *OTUD7A* in mouse tissues including brain and spleen was observed (Figure S14F, Supporting Information). Expression of *OTUD7A* was also reported in different cancer types by Human Protein Atlas (Figure S15A, Supporting Information). Importantly, we observed *OTUD7A* expression in metastatic Ewing sarcoma tumors. *OTUD7A* was detected in tumor cells identified by *CD99* and *FLI1* antibody staining (**Figure** **5**A and Figure S15B (Supporting Information)). These data suggest that *OTUD7A* is expressed in Ewing sarcomas and that the enzymatic activity offers a therapeutic target.

### 2.9 Artificial‐Intelligence‐Aided Virtual Drug Screen Identified 7Ai as an *OTUD7A* Catalytic Inhibitor

To rapidly assess the binding ability of drug‐like small molecules to *OTUD7A*, we applied AtomNet, a structure‐based deep convolutional neural network virtual screening technology developed by Atomwise Inc.^[^ ^47^, ^48^ ^]^ In the absence of a published crystal structure of the *OTUD7A–OTU* domain, we first generated a homology model of the *OTUD7A–OTU* domain based on the available crystal structure of the closely related *OTUD7B–OTU* domain (Protein Data Bank (PDB): 5LRW, 79% sequence identity in this region) (Figure S16A, Supporting Information). Using this generated structure, we performed a virtual screen by sifting through a library of 4 million commercially available, drug‐like compounds that yielded a chemically diverse set of 73 high‐scoring predicted hits. We evaluated these compounds for their ability to reduce *EWS–FLI1* protein abundance in both A673 and SK‐N‐MC cells (Figure S16B,C, Supporting Information). One compound that we termed as 7Ai, ranking 44th out of 4 025 533 compounds we screened, reduced *EWS–FLI1* protein levels in both Ewing cells without affecting *OTUD7A* protein levels (Figure S16B,C, Supporting Information). Moreover, 7Ai reduced *EWS–FLI1* protein levels in a dose‐dependent manner within 12 h (Figure 5B and Figure S16D (Supporting Information)). Importantly, this activity was not lost following high performance liquid chromatography (HPLC) purification (Figure S16D, Supporting Information), suggesting that 7Ai, rather than contaminants from the chemical synthesis process, mediates *OTUD7A* suppression. In addition, 7Ai efficiently blocked *OTUD7A*‐mediated deubiquitination of *EWS–FLI1* in cells (Figure 5C and Figure S16E (Supporting Information)). Consistent with the genetic *OTUD7A* depletion, 7Ai reduced *EWS–FLI1* protein abundance in parental A673 but not A673*^3A^* (Figure 5D and Figure S16F (Supporting Information)), highlighting the importance of the *OTUD7A*/*EWS–FLI1* signaling in mediating 7Ai function. 7Ai did not interfere with *OTUD7A* binding to *EWS–FLI1* (Figure S16G, Supporting Information), suggesting this compound might suppress *OTUD7A* catalytic activity through interaction with the catalytic domain. To explore whether 7Ai directly binds *OTUD7A*, we purified the bacterially produced His‐tagged *OTUD7A OTU* domain (aa183‐449) (Figure S16H, Supporting Information). Using isothermal titration calorimetry (ITC), we demonstrated a binding affinity of 7Ai in vitro of ≈1.1 × 10^−6^ m (stoichiometry is about 1:1) (Figure S16I, Supporting Information). 7Ai efficiently reduced *EWS–FLI1* protein expression in multiple Ewing sarcoma cells in addition to A673, including MHH‐ES‐1 and EWS894 (Figure 5E), SK‐N‐MC and EWS502 (Figure S17A, Supporting Information). Notably, 7Ai treatment did not significantly affect *OTUD7B* activities as indicated by negligible changes in known *OTUD7B* substrates, including mechanistic target of rapamycin complex 2 (*mTORC2*)^[^ ^40^ ^]^ and anaphase promoting complex (*APC*)/*Cdh1* ^[^ ^42^ ^]^ (Figure 5E and Figure S17A (Supporting Information)). These data support that compound 7Ai suppresses *OTUD7A* activity to destabilize *EWS–FLI1*.

### 2.10 7Ai Impedes Ewing Sarcoma Growth In Vitro and In Vivo

We then evaluated the effects of 7Ai treatment on Ewing sarcoma growth. 3 days treatment with 7Ai reduced proliferation of A673, MHH‐ES‐1, and EWS894 cells (Figure 5F–H), which was associated with reduced *EWS–FLI1* protein abundance (Figure 5E and Figure S17B,C (Supporting Information)). Notably, this effect was not observed in A673*^3A^* cells (Figure 5I). 7Ai also reduced *EWS–FLI1* protein abundance in A673‐teton‐sh*OTUD7A* but not same cells reconstituted with *EWS–FLI1*‐*3A* (Figure S17D, Supporting Information). Importantly, 7Ai treatment failed to significantly suppress growth of A673 cells expressing *EWS–FLI1*‐*3A* (Figure S17E, Supporting Information). 7Ai treatment reduced transcription of *EWS–FLI1* target genes (*NKX2.2* and *PSPH*, Figure 5K,L), and increased transcription of genes negatively regulated by *EWS–FLI1* (*LOX* and *TGFBR2*, Figure 5M,N). 7Ai did not affect *EWS–FLI1* mRNA levels (Figure S17F, Supporting Information). Importantly, 2 week treatment of 7Ai led to reduced colony formation ability of MMH‐ES‐1 (Figure 5J) and A673 cells (Figure S17G, Supporting Information) in vitro. Cumulatively, these data support that 7Ai suppresses Ewing sarcoma growth by reducing *EWS–FLI1* protein stability.

We then examined the effect of 7Ai on Ewing sarcoma cells grown as xenografts. Following establishment of ≈0.5 cm A673 tumors in immunocompromised mice, we administrated vehicle or 7Ai (25 mg kg^−1^, intraperitoneal (IP)) or vehicle every 2–3 days (Figure 5O). Compared with vehicle control group, 7Ai treatment significantly reduced tumor volume (Figure 5P and Figure S17H (Supporting Information)) and tumor growth (Figure 5Q,R). Notably, 7Ai administration over the 3‐week treatment period did not significantly affect body weight (Figure S17I, Supporting Information). 7Ai‐treated tumors demonstrated reduced *EWS–FLI1* protein and cell proliferation (Ki67 staining) and increased apoptosis (cleaved‐caspase3 staining) (Figure S17J, Supporting Information). In vitro, 7Ai treatment significantly reduced A673 cell migratory ability (Figure S17K,L, Supporting Information). These data indicate that 7Ai suppresses Ewing sarcoma growth and migration.

To examine if 7Ai exerts selectivity in eradicating Ewing sarcoma cells, we treated two Ewing sarcoma cell lines A673 and MHH‐ES‐1 and two normal control cell lines human umbilical vein endothelial cells (HUVEC) and foreskin fibroblast (FF) with 7Ai for 3 days in vitro. 7Ai treatment efficiently reduced *EWS–FLI1* protein abundance in both Ewing sarcoma cells (Figure S18A,B, Supporting Information) but had minimal effects on *FLI1* proteins in HUVEC and FF cells (Figure S18C,D, Supporting Information). As observed with A673, 7Ai treatment reduced MHH‐ES‐1 proliferation but exerted neglectable effects in HUVEC and FF cells (Figure 5S). Although preliminary, the limited in vivo side‐effect profile and effect on non‐Ewing sarcoma cells offers the possibility of a therapeutic window for Ewing sarcoma treatment. Subsequent formal in vivo studies will be necessary to support this observation.

### 2.11 *OTUD7A* Might Also Control *EWS–ERG* Fusion Protein Stability in Ewing Sarcoma

In addition to *EWS–FLI1* fusion observed in ≈85% Ewing sarcoma tumors, other fusions including *EWS–*transcriptional regulator ERG (*ERG*) (≈10% patients) and *EWS–*protein FEV (*FEV*) (≈1% patients) have also been observed. Variation in translocation breakpoints result in type I and type II fusions which differ based on included exons (Figure S19A,B, Supporting Information). Our data suggest that *SPOP*/*CK1* and *OTUD7A* regulate both type I and type II fusions, as the *SPOP* degron is present in both fusion types (Figure S19A–C, Supporting Information). Moreover, as predicted by the presence of the *SPOP* degron in the *ERG* segment in *EWS–ERG* fusion (Figure S19A, Supporting Information), *SPOP* also targeted *EWS–ERG* for degradation (Figure S19D, Supporting Information), and *OTUD7A* stabilized *EWS–ERG* (Figure S19E, Supporting Information). *EWS–ERG* could partially replace *EWS–FLI1* in A673 cells to maintain cell growth in vitro (Figure S19F,G, Supporting Information). In this setting, *SPOP* depletion stabilized *EWS–ERG* (Figure S19H, Supporting Information). *OTUD7A* depletion reduced *EWS–ERG* (Figure S19I, Supporting Information) associated with reduced cell growth (Figure S19J), Supporting Information. 7Ai treatment reduced *EWS–ERG* protein levels (Figure S19K, Supporting Information). Cumulatively, these data support that the vast majority of Ewing sarcoma would be targets of *OTUD7A*‐directed treatment. More broadly, this project offers a strategy to therapeutically target a critical oncoprotein initiated by the recognition of a putative protein degron sequence.

## 3 Discussion

Because it is indispensable for Ewing sarcoma growth, targeting the *EWS–FLI1* fusion oncoprotein offers an important and specific therapeutic strategy. Here, we report the identification of a pathophysiologically relevant protein control mechanism. *SPOP* is the first E3 ubiquitin ligase that targets *EWS–FLI1* for ubiquitination and degradation in a *CK1*‐phosphorylation‐dependent manner. The deubiquitinase *OTUD7A* antagonizes *SPOP* function to stabilize *EWS–FLI1*, revealing *OTUD7A* as a new Ewing‐sarcoma‐growth‐dependent gene. Applying quantitative proteomic analyses, we confirmed *EWS–FLI1* as a bona fide *OTUD7A* substrate and identified additional *OTUD7A* substrates that may mediate cellular motility, independent of *EWS–FLI1*. Since, genetic inactivation of *OTUD7A* reduced Ewing sarcoma proliferation and motility, sought to target *OTUD7A*. Using artificial‐intelligence (AI)‐aided virtual drug screening, we identify the first *OTUD7A* catalytic inhibitor, which limits Ewing sarcoma growth in vitro and in mice by degrading *EWS–FLI1*.

The *FLI1* domain in *EWS–FLI1* is targeted by *SPOP* and *OTUD7A*. *FLI1* has tissue restricted expression and deficiency is associated with thrombocytopenia in humans and mice. *FLI1* is not broadly considered an essential gene for cell proliferation (such as MDA‐MD‐231 (Figure 3G and Figure S9F,G (Supporting Information)) although exceptions include certain cancers such as blood and kidney cancer (Figure S20A,B (Supporting Information) from DEPMAP portal). In support of this association, Tet‐induced depletion of *OTUD7A* in kidney cancer (ACHN) and leukemia (Jurkat and CUTLL1) cells reduced levels of endogenous *FLI1* (Figure S20C–E, Supporting Information) accompanied by reduced cell proliferation (Figure S20C–E, Supporting Information). Like genetic *OTUD7A* depletion, pharmacological inhibition of *OTUD7A* by the compound 7Ai also decreased proliferation of Jurkat cells (Figure S20F, Supporting Information). Therefore, in addition to Ewing sarcoma, targeted inhibition of *OTUD7A* may be relevant for other cancers dependent on *FLI1* for proliferation, such as leukemia and kidney cancer (Figure S20G, Supporting Information).

Notably, due to the lack of a large cohort of patient data in Ewing sarcoma as a rare cancer, analyzing the Cancer Genome Atlas (TCGA) sarcoma dataset revealed that *OTUD7A* gene was infrequently altered (Figure S21A, Supporting Information). *OTUD7A* mRNA levels were not associated with overall patient survival in adult soft tissue sarcomas analyze by TCGA (Figure S21B, Supporting Information). Whether *OTUD7A* protein abundance predicts Ewing sarcoma patient survival remains to be determined. Interestingly, high *OTUD7A* mRNA levels were associated with worse patient survival in thymoma (Figure S21C, Supporting Information), uterine corpus endometrial carcinoma (Figure S21D, Supporting Information), and esophageal squamous cell carcinoma (Figure S21E, Supporting Information) patient cohorts, and neared statistical significance for worse breast cancer survival (Figure S21F, Supporting Information). By contrast, high *OTUD7A* mRNA expression was not associated with survival in ovarian (Figure S21G, Supporting Information) and stomach (Figure S21H, Supporting Information) cancers, and with improved survival of cervical cancer patients (Figure S21I, Supporting Information).

Two deubiquitinases, *USP7* ^[^ ^18^ ^]^ and *USP19* ^[^ ^49^ ^]^ had been reported as vulnerabilities in Ewing sarcoma. Genetic and pharmacologic inactivation of *USP7* was shown to reduce Ewing sarcoma growth, although the substrate(s) through which *USP7* acted on Ewing sarcoma growth remained unclear.^[^ ^18^ ^]^ Genetic depletion of *USP19* reduced Ewing sarcoma cell growth in vitro and in mice largely through destabilizing *EWS–FLI1* proteins.^[^ ^19^ ^]^

In our study, we find that both genetic and pharmacological inactivation of *OTUD7A* impede not only Ewing sarcoma growth but also decreased motility. It is possible that the activity of *OTUD7A* on motility is through *EWS–FLI1*‐independent substrates such as *ITGAV* and *COL3A*. Thus, it seems that inhibiting *OTUD7A* suppresses both Ewing sarcoma proliferation and may affect its ability to disseminate. Whole‐animal *OTUD7A* deletion in mice led to decreased dendritic spine density that mimicked neurodevelopmental disorders^[^ ^50^ ^]^ associated with 15q13.3 microdeletion syndrome.^[^ ^51^ ^]^ Recently, a homozygous *OTUD7A–L233F* mutation was found in a patient with the 15q13.3 microdeletion syndrome with characterized proteasome dysfunction presumably caused by the loss of function of the *OTUD7A* deubiquitinase activity.^[^ ^52^ ^]^ Although it remains unclear if these neurological disorders caused by *OTUD7A* dysfunction are limited to changes in dendritic spines, these results offer additional considerations if 7Ai or other *OTUD7A* inhibitors begin preclinical evaluation for Ewing sarcoma.

Our studies demonstrated efficacy of 7Ai in vivo and in vitro efficacy in the micromolar range. Additional medicinal chemistry studies are needed to further improve its potency and to evaluate pharmacokinetic and side effect properties. Since therapy for initial and relapsed Ewing sarcoma includes cytotoxic chemotherapies, it would be of interest to evaluate the combination of 7Ai with active chemotherapeutic drugs, including the treatment of patients with metastatic disease. Because of the development of other biologically targeted therapies, including those directed at *EWS–FLI1* and *USP19* and *USP7*, assaying the activity of 7Ai with these agents would also be of interest.

## 4 Experimental Section

### Cell Culture and Transfection

HEK293, HEK293T, FF, A673, MHH‐ES‐1, MDA‐MB‐231 and ACHN cells were cultured in Dulbecco's Modified Eagle Medium (DMEM) supplemented with 10% fetal bovine serum (FBS). Jurkat and CUTLL1 cells were cultured in RPMI‐1640 medium supplemented with 10% FBS. EWS502 and EWS894 cells were maintained in RPMI‐1640 medium supplemented with 15% FBS. SK‐N‐MC were cultured in RPMI‐1640 medium supplemented with 10% FBS, 200 × 10^−6^ m glutamine (Gibco, 25030081) and nonessential amino acids (Gibco, 11140050). HUVEC cells were cultured in Endothelial Cell Growth Medium 2 (PromoCell, C‐22111) supplemented with 10% FBS. All cell culture media were supplemented with 100 units of penicillin and 100 mg mL^−1^ streptomycin unless otherwise stated.

Cell transfection was performed using lipofectamine 3000 or polyethylenimine, as described previously.^[^ ^53^, ^54^, ^55^ ^]^ Packaging of lentiviral shRNA or complementary DNA (cDNA) expressing viruses, as well as subsequent infection of various cell lines were performed according to the protocols described previously.^[^ ^56^, ^57^ ^]^ Following viral infection, cells were maintained in the presence of blasticidin (5 µg mL^−1^) or puromycin (1 µg mL^−1^), depending on the viral vector used to infect cells.

MG132 (S2619), MLN4924 (S7109), cycloheximide (S6611), D4476 (S7642), and lenalidomide (S1029) were purchased from Selleck. Tetracycline (87128) and doxycycline (D9891) were purchased from Sigma‐Aldrich. JQ1 was purchased from Sigma (SML0974). Larger quantities of compound 7Ai was purchased from Princetonbio or obtained from Atomwise, Inc.

### Plasmid Construction

Flag‐*SPOP* and CMV‐glutathione‐S‐transferase (*GST*)‐*SPOP* were as described previously.^[^ ^29^ ^]^ pCDNA3‐HA‐*SPOP* plasmid was constructed by cloning *SPOP* into pCDNA3‐HA vector using primers listed below. *Myc*‐tagged *CK1s* and *CK2s* were as described.^[^ ^29^ ^]^ His‐ub plasmids were as described.^[^ ^58^ ^]^ *Myc*‐Flag‐*OTU* plasmids were as described.^[^ ^40^ ^]^ His‐*OTUD7A* was constructed by cloning *OTUD7A* into pET28a vector using primers listed below. HA‐*EWS–FLI1* and HA‐*FLI1* were cloned into pCDNA3‐HA vector using primers listed below. pLenti‐HA‐*FLI1*‐WT and *3A* plasmids were cloned into the pLenti‐HA‐hygro vector using primers listed below. pLL5.5‐HA‐*EWSR1* and pLL5.5‐HA‐*EWS–ERG* were previously described.^[^ ^2^ ^]^ HA‐*SPOP* was cloned into pCDNA3‐HA vector using primers listed below. His‐*OTUD7A* was cloned into pET28a vector using primers listed below. pLenti‐*EWS–FLI1*‐*3A* was constructed by cloning *EWS–FLI1*‐*3A* into pLenti‐GFP‐hygro vector using primer below from Flag‐*EWS–FLI1*‐*3A* plasmid. *Myc*–*cullin* plasmids were a generous gift from Yue Xiong lab at University of North Carolina at Chapel Hill.

*EWS–FLI*‐BglII‐F: GCATAGATCTGCGTCCACGGATTACAGTACC
*FLI1*‐BglII‐F: GCATAGATCTGACGGGACTATTAAGGAGGC
*FLI1*‐XhoI‐R: GCATCTCGAGCTAGTAGTAGCTGCCTAAGTG
h*SPOP*‐BamHI‐F: GCATGGATCCTCAAGGGTTCCAAGTCCTCCAC
h*SPOP*‐XhoI‐R: GCATCTCGAGTTAGGATTGCTTCAGGCGTTTGCG
*OTUD7A*‐BglII‐F: GCATAGATCTGTTTCTAGTGTGCTTCCAAACC
*OTUD7A*‐SalI‐R: GCATGTCGACTCACAGCTCCTCGCGG

*EWS–FLI1*‐*3A*, *OTUD7A–C210S*, and *CUL3–E358Q* mutants were generated using the QuikChange XL Site‐Directed Mutagenesis Kit (Stratagene) according to the manufacturer's instructions. Details of plasmid constructions were available upon request.

EWS–FLI‐3A‐F: CCTCCATGCCTGTCACTGCCGCCGCCTTCTTTGGAGCCGCATCAC
EWS–FLI‐3A‐R: GTGATGCGGCTCCAAAGAAGGCGGCGGCAGTGACAGGCATGGAGG
OTUD7A–C210S‐F: CAGGGGATGGGAACTCCCTTTTACATGCTGCTTCACTG
OTUD7A–C210S‐R: CAGTGAAGCAGCATGTAAAAGGGAGTTCCCATCCCCTG
CUL3–E358Q‐F: GTTCGATCGCTTCCTCCTGCAATCATTCAACAATGACCGTCTC
CUL3–E358Q‐R: GAGACGGTCATTGTTGAATGATTGCAGGAGGAAGCGATCGAAC

Reverse transcription PCR (RT‐PCR) primers to examine *EWS–FLI1* mRNA changes upon *SPOP* or *OTUD7A* depletion were listed below:

*EWS*‐F: TCCTACAGCCAAGCTCCAAGTC
*FLI1*‐R: ACTCCCCGTTGGTCCCCTCC

RT‐PCR primers to examine *EWS–FLI1* transcriptional targets used in this study were listed below:

*EWS–FLI1*‐F: CAGTCACTGCACCTCCATCC
*EWS–FLI1*‐R: TTCATGTTATTGCCCCAAGC
*NKX2.2*‐F: CTACGACAGCAGCGACAACC
*NKX2.2*‐R: GCCTTGGAGAAAAGCACTCG
*TGFBR2*‐F: CATCTGTGAGAAGCCACAGG
*TGFBR2*‐R: TGCACTCATCAGAGCTACAGG
insulin‐like growth factor‐binding protein 3 (*IGFBP3*)‐F: CTGCTCAGATTTCCCCAAAG
*IGFBP3*‐R: TGGCATCAAGCAGGTCATAG
*LOX*‐F: CATCAAGAAAGGGCATGCTAA
*LOX*‐R: CTACGGCAGGGACCATATTCT
janus kinase 1 (*JAK1*)‐F: CAGGTCTCCCACAAACACATCG
*JAK1*‐R: ACCAGGTCTTTATCCTCCAAGTAGC
G1/S‐specific cyclin‐D1 (*CCND1*)‐F: CGCACGATTTCATTGAACACTT
*CCND1*‐R: CGGATTGGAAATACTTCACAT
*CCND3*‐F: CCTCTGTGCTACAGATTATACCTTTGC
*CCND3*‐R: TTGCACTGCAGCCCCAAT
glutathione S‐transferase Mu 4 (*GSTM4*)‐F: TGGAGAACCAGGCTATGGACGT
*GSTM4*‐R: CCAGGAACTGTGAGAAGTGCTG
D‐3‐phosphoglycerate dehydrogenase (*PHGDH*)‐F: CTGCGGAAAGTGCTCATCAGT
*PHGDH*‐R: TGGCAGAGCGAACAATAAGGC
*PSPH*‐F: GATGCTGTGTGTTTTGATGTTGAC
*PSPH*‐R: CTTGACTTGTTGCCTGATCACATT
neutral amino acid transporter B(0) (*SLC1A5*)‐F: CTTGGTAGTGTTTGCCATCGT
*SLC1A5*‐R: TGCGGGTGAAGAGGAAGTAG
monofunctional C1‐tetrahydrofolate synthase, mitochondrial (*MTHFD1L*)‐F: GAGCTCTGAAGARGCATGGAG
*MTHFD1L*‐R: TGCTTCTGGAGGTTACAGCA

### shRNAs and sgRNAs

shRNA vectors to deplete endogenous *SPOP*, *CUL3*, and various *OTU*s were purchased from Sigma. Their sequence was listed below:

sh*SPOP*‐1: CCGGCACAGATCAAGGTAGTGAAATCTCGAGATTTCACTACCTTGATCTGTGTTTTTTG
sh*SPOP*‐2: CCGGCAAGGTAGTGAAATTCTCCTACTCGAGTAGGAGAATTTCACTACCTTGTTTTTTG
sh*SPOP*‐3: CCGGCAAACGCCTGAAGCAATCCTACTCGAGTAGGATTGCTTCAGGCGTTTGTTTTTTG
sh*SPOP*‐4: CCGGCTCCTACATGTGGACCATCAACTCGAGTTGATGGTCCACATGTAGGAGTTTTTTG
sh*CUL3*‐1: CCGGCGTGTGCCAAATGGTTTGAAACTCGAGTTTCAAACCATTTGGCACACGTTTTTG
sh*CUL3*‐2: CCGGTTCAGGCTTTACAACGTTTATCTCGAGATAAACGTTGTAAAGCCTGAATTTTTG
sh*CUL3*‐3: CCGGCGTGTGCCAAATGGTTTGAAACTCGAGTTTCAAACCATTTGGCACACGTTTTTG
sh*OTUB1*‐1: CCGGAGGAGTATGCTGAAGATGACACTCGAGTGTCATCTTCAGCATACTCCTTTTTT
sh*OTUB1*‐2: CCGGTGTTTCTATCGGGCTTTCGGACTCGAGTCCGAAAGCCCGATAGAAACATTTTT
sh*OTUB1*‐3: CCGGTGTGGTTGTAAATGGTCCTATCTCGAGATAGGACCATTTACAACCACATTTTT
sh*OTUB2*‐1: CCGGCCTATGTGTCACTGGATTATTCTCGAGAATAATCCAGTGACACATAGGTTTTTG
sh*OTUB2*‐2: CCGGTGGGCTGCTATGTCTCTGTATCTCGAGATACAGAGACATAGCAGCCCATTTTT
sh*OTUB2*‐3: CCGGCCTTCCGTTTACCTGCTCTATCTCGAGATAGAGCAGGTAAACGGAAGGTTTTT
sh*OTUD3*‐1: CCGGGACGTCTGCCATCGCATATTACTCGAGTAATATGCGATGGCAGACGTCTTTTTG
sh*OTUD3*‐2: CCGGTTTGGAAATCAGGGCTTAAATCTCGAGATTTAAGCCCTGATTTCCAAATTTTTG
sh*OTUD3*‐3: CCGGGGGAGTTACACATCGCATATCCTCGAGGATATGCGATGTGTAACTCCCTTTTTG
sh*OTUD4*‐1: CCGGCAAGTCGAGAATCTAACTATTCTCGAGAATAGTTAGATTCTCGACTTGTTTTTG
sh*OTUD4*‐2: CCGGTATGCAATGCCTTAGTCATAACTCGAGTTATGACTAAGGCATTGCATATTTTTG
sh*OTUD4*‐3: CCGGCACTATAGATTCCAAACATAACTCGAGTTATGTTTGGAATCTATAGTGTTTTTG
sh*OTUD5*‐1: CCGGCCATCATTCAAACCAGGGTTTCTCGAGAAACCCTGGTTTGAATGATGGTTTTTTG
sh*OTUD5*‐2: CCGGCCGACTACTTCTCCAACTATGCTCGAGCATAGTTGGAGAAGTAGTCGGTTTTTG
sh*OTUD5*‐3: CCGGAGAACGTCTGAGCCTTCAATGCTCGAGCATTGAAGGCTCAGACGTTCTTTTTTG
sh*OTUD6A*‐1: CCGGCATGATCTACTGCGACAACATCTCGAGATGTTGTCGCAGTAGATCATGTTTTTTG
sh*OTUD6A*‐2: CCGGCACCAACTAAGATTTGGTCATCTCGAGATGACCAAATCTTAGTTGGTGTTTTTTG
sh*OTUD6A*‐3: CCGGGATTTGGTCATGTTGCGTATACTCGAGTATACGCAACATGACCAAATCTTTTTTG
sh*OTUD6B*‐1: CCGGGCAAAGCTACTAACAGGTGTTCTCGAGAACACCTGTTAGTAGCTTTGCTTTTTTG
sh*OTUD6B*‐2: CCGGGCTGACTACTAAGGAGAATAACTCGAGTTATTCTCCTTAGTAGTCAGCTTTTTTG
sh*OTUD6B*‐3: CCGGCGATGAGACTAATGCAGTGAACTCGAGTTCACTGCATTAGTCTCATCGTTTTTTG
sh*OTUD7A*‐1: CGGGCAGCAATTCTAACAGCAATACTCGAGTATTGCTGTTAGAATTGCTGCTTTTTG
sh*OTUD7A*‐2: CCGGCGCACACACTTCAGCAAGAATCTCGAGATTCTTGCTGAAGTGTGTGCGTTTTTG
sh*OTUD7A*‐3: CCGGGCGCGAGAACTGTGCGTTCTACTCGAGTAGAACGCACAGTTCTCGCGCTTTTTG
sh*OTUD7B*‐1: GTACCGGTTGAAGAGTTTCACGTCTTTGCTCGAGCAAAGACGTGAAACTCTTCAATTTTTTG
sh*OTUD7B*‐2: CCGGTGGAAATGCTCACGGTTTATACTCGAGTATAAACCGTGAGCATTTCCATTTTTG
sh*OTUD7B*‐3: CCGGGCAAGGAGGCTAAACAAAGTTCTCGAGAACTTTGTTTAGCCTCCTTGCTTTTT
sh*CK1α*‐42: CCGGGCAGAATTTGCGATGTACTTACTCGAGTAAGTACATCGCAAATTCTGCTTTTT
sh*CK1α*‐87: CCGGGCAAGCTCTATAAGATTCTTCCTCGAGGAAGAATCTTATAGAGCTTGCTTTTTTG
sh*FLI1*: TGCCCATCCTGCACACTTACTTCAAGAGAGTAAGTGTGCAGGATGGGCTTTTTTC (targeting the 3ʹuntraslated region (UTR) of *FLI1* as reported in ref. [2])

Teton‐sh*OTUD7A* primers were listed below:

Teton‐sh*OTUD7A*‐F: CCGGGCGCGAGAACTGTGCGTTCTACTCGAGTAGAACGCACAGTTCTCGCGCTTTTT
Teton‐sh*OTUD7A*‐R: AATTAAAAAGCGCGAGAACTGTGCGTTCTACTCGAGTAGAACGCACAGTTCTCGCGC

sh*OTUD7A*‐resistant *OTUD7A* construct was generated using the QuikChange XL Site‐Directed Mutagenesis Kit (Stratagene) according to the manufacturer's instructions.

sh*OTUD7A*‐62‐resistant‐F: CTGCCAGCGGGAAAATTGCGCGTTCTACGG
sh*OTUD7A*‐62‐resistant‐R: CCGTAGAACGCGCAATTTTCCCGCTGGCAG

*EWS–FLI1*‐*3A* knock‐in experiment was performed using *EWS–FLI1* sgRNAs and single‐stranded donor oligonucleotides (ssoDNA) as listed below:

*EWS–FLI1*‐*3A*‐sgRNA‐F: CACCG TGCGGCTCCAAAGAAGCTGG
*EWS–FLI1*‐*3A*‐sgRNA‐R: AAAC CCAGCTTCTTTGGAGCCGCA C
*EWS–FLI1*‐*3A*‐ssoDNA: GCCCACCAGCAGAAGGTGAACTTTGTCCCTCCCCATCCATCCTCCATGCCTGTCACTGCCGCCGCCTTCTTTGGAGCCGCATCACAATACTGGACCTCCCCCACGGGGGGAATCTACCCC

Knock‐in clones were screened by PCR using primers listed below to search for clone loss of BpmI site after knock‐in.

*EWS–FLI*‐*3A*‐KI‐PCR‐F: GTGCACGGCAAAAGATATGCTTAC
*EWS–FLI*‐*3A*‐KI‐PCR‐R: CTAGTAGTAGCTGCCTAAGTGTG

sgRNAs to stably deplete endogenous *OTUD7A* were listed below:

sg*OTUD7A*‐1A‐F: CACCGAGACTTGTTCGGTCCACGG
sg*OTUD7A*‐1A‐R: AAACCCGTCCACCGAACAAAGTCTC
sg*OTUD7A*‐1B‐F: CACCGTGCTGCCCAACACTCAGCCG
sg*OTUD7A*‐1B‐R: AAACCGGCTGAGTGTTGGGAGCAC
sg*OTUD7A*‐1C‐F: CACGCAGACCAGGTTCTGCCCCCG
sg*OTUD7A*‐1C‐R: AAACCGGGGGCAGAACCTGGTCTGC

### Immunoblot and Immunoprecipitations Analyses

Cells were lysed in EBC buffer (50 × 10^−3^ m Tris pH 7.5, 120 × 10^−3^ m NaCl, 0.5% NP‐40) or Triton X‐100 buffer (50 × 10^−3^ m Tris pH 7.5, 150 × 10^−3^ m NaCl, 1% Triton X‐100) supplemented with protease inhibitors (Complete Mini, Roche) and phosphatase inhibitors (phosphatase inhibitor cocktail sets I and II, Calbiochem). The protein concentrations of whole cell lysates were measured by NanoDrop OneC using the Bio‐Rad protein assay reagent as described previously.^[^ ^55^ ^]^ Equal amounts of whole cell lysates were resolved by sodium dodecyl sulfate polyacrylamide gel electrophoresis (SDS‐PAGE) and immunoblotted with indicated antibodies. For immunoprecipitation analysis, unless specified, 1000 µg lysates were incubated with the indicated antibody (1–2 µg) for 3–4 h at 4 °C followed by 1 h incubation with 10 µL Protein A magnetic beads (New England Biolabs). Or, 1000 µg lysates containing tagged molecules were incubated with agarose‐bead‐coupled antibodies for the specific tag for 3–4 h at 4 °C. For endogenous IPs, incubation of cell lysates with antibodies was extended to overnight. The recovered immunocomplexes were washed 5 times with NETN buffer (20 × 10^−3^ m Tris, pH 8.0, 100 × 10^−3^ m NaCl, 1 × 10^−3^ m ethylenediaminetetraacetic acid (EDTA), and 0.5% NP‐40) before being resolved by SDS‐PAGE and immunoblotted with indicated antibodies.

### Antibodies

All antibodies were used at a 1:1000 dilution in Tris buffered saline with Tween 20 (TBST) buffer with 5% nonfat milk for western blotting. Anti‐*GST* antibody (2625), anti‐*Cullin3* antibody (2759), anti‐*CD99* antibody (20992), anti‐*CK1* antibody (2655), anti‐*BRD4* antibody (13440), anti‐*Plk1* antibody (4513), anti‐*Akt–pS473* antibody (4060), anticleaved‐*caspase 3* antibody (9661), anti c‐*Myc* antibody (5605), and anti‐*myc*‐tag antibody (2278) were obtained from Cell Signaling Technology. Anti‐*FLI1* antibody (ab180902), anti‐nuclear factor erythroid 2‐related factor 2 (NRF2) (ab62352), anti‐*ERG* (ab92513), and anti‐Ki67 antibody (ab254123) were obtained from Abcam. Anti‐*SPOP* antibody (16750‐1‐AP) was purchased from Proteintech. Anti‐*EWSR1* antibody (A300‐417) was purchased from Bethyl Laboratories. Polyclonal anti‐HA antibody (sc‐805), anti‐p27‐antibody (sc1641), anti‐NRF2 antibody (sc81342), anti‐*ERG* antibody (271048), anti‐*ITGAV* antibody (376156), anti‐*COL3A1* antibody (271249), and anti‐Vinculin antibody (sc‐25336) were obtained from Santa Cruz Biotechnology. Polyclonal anti‐Flag antibody (F‐2425), monoclonal anti‐Flag antibody (F‐3165, clone M2), anti‐Tubulin antibody (T‐5168), anti‐*OTUD3* antibody (PA5‐98487), anti‐*OTUD7A* antibody (SABB04135), anti‐Flag agarose beads (A‐2220), anti‐HA agarose beads (A‐2095), glutathione agarose beads (G4510), peroxidase‐conjugated anti‐mouse secondary antibody (A‐4416), and peroxidase‐conjugated anti‐rabbit secondary antibody (A‐4914) were obtained from MilliporeSigma. Monoclonal anti‐HA antibody (MMS‐101P) was obtained from BioLegend.

### Generation of *EWS–FLI1*‐*3A* Knock‐In A673 Cells

Parental A673 cells were split into 24‐well plates and transfected with sgRNA against *EWS–FLI1* together with *EWS–FLI1*‐*3A*‐ssoDNA following protocols as described.^[^ ^59^ ^]^ 1 day post‐transfection, cells were selected with 1 µg mL^−1^ puromycin for 3 days. Surviving cells were counted and each single cell was seeded into 96‐well plates. Each single clone grown up in 96‐well plates was amplified and one copy was used for genomic DNA extraction, followed by PCR and BpmI digestion to screen for potential knock‐in clones. BpmI negative clones were selected and sequenced to verify the knock‐in at the DNA level. 3 isogenic knock‐in clones were selected and saved.

### shRNA‐Mediated *OTU* Screen to Identify *OTUs* Critical to Maintain Ewing Sarcoma Growth

Three independent shRNAs against each *OTU* member were selected and lentiviruses expressing each shRNA was individually packaged following protocols as described.^[^ ^55^ ^]^ A673 cells were infected with each individual shRNA expressing lentiviruses for 24 h, recovered for 72 h before 1000 surviving cells from each group were seeded in 96‐well plates in triplicates. 72 h later, MTT assays were performed to determine cell viability.

### Sample Preparation for Proteomic Analysis

A673 cells were treated with H_2_O or 1 µg mL^−1^ tetracycline (to induce a Tet‐inducible *OTUD7A* depletion) for 72 h (*n* = 3 biological replicates per time point). Cells were washed 3 times with ice‐cold phosphate‐buffered saline (PBS), then lysed in 8 m urea, Tris‐HCl (pH 7.6) with protease and phosphatase inhibitors (Bimake). Lysates were reduced with 5 × 10^−3^ m dithiothreitol (DTT), alkylated with 15 × 10^−3^ m iodoacetamide, then subjected to digestion with LysC (Wako) for 2 h, then trypsin (Promega) overnight at 37 °C at a 1:50 enzyme:protein ratio. The resulting peptide samples were acidified, desalted using Thermo desalting spin columns, then the eluates were dried via vacuum centrifugation. Peptide concentration was determined using Pierce Quantitative Colorimetric Peptide Assay. 40 µg of each sample was reconstituted with 50 × 10^−3^ m 4‐(2‐hydroxyethyl)‐1‐piperazineethanesulfonic acid (HEPES) pH 8.5, then individually labeled with 60 µg of TMT (tandem mass tag) 10plex reagent (Thermo Fisher) for 1 h at room temperature. Labeling efficiency was evaluated by liquid chromatography with tandem mass spectrometry (LC–MS/MS) analysis of a pooled test mix. Samples were quenched with 50% hydroxylamine to a final concentration of 0.4%. Labeled peptide samples were pooled, desalted using Thermo desalting spin column, and dried via vacuum centrifugation. The dried TMT‐labeled sample was fractionated using high pH reversed phase HPLC.^[^ ^60^ ^]^ Briefly, the sample was offline fractionated over a 90 min run, into 96 fractions by high pH reverse‐phase HPLC (Agilent 1260) using an Agilent Zorbax 300 Extend‐C18 column (3.5 µm, 4.6 × 250 mm) with mobile phase A containing 4.5 × 10^−3^ m ammonium formate (pH 10) in 2% v/v LC–MS grade acetonitrile, and mobile phase B containing 4.5 × 10^−3^ m ammonium formate (pH 10) in 90% v/v LC–MS grade acetonitrile. The 96 resulting fractions were then pooled in a noncontinuous manner into 24 fractions. The 24 fractions were dried via vacuum centrifugation.

### LC/MS/MS Analyses

The 24 fractions were analyzed by LC/MS/MS using an Easy nLC 1200 coupled to a QExactive HF mass spectrometer (Thermo Scientific). Samples were injected onto an EASY‐Spray PepMap C18 column (75 µm id × 25 cm, 2 µm particle size) (Thermo Scientific) and separated over a 150 min method. The gradient for separation consisted of 5–50% mobile phase B at a 250 nL min^−1^ flow rate, where mobile phase A was 0.1% formic acid in water and mobile phase B consisted of 0.1% formic acid in 80% acetonitrile (ACN). The QExactive HF was operated in data‐dependent mode where the 15 most intense precursors were selected for subsequent higher‐energy collisional dissociation (HCD) fragmentation. Resolution for the precursor scan (*m*/*z* 350–1600) was set to 60 000 with a target value of 3 × 10^6^ ions and a maximum injection time of 100 ms. MS/MS scan resolution was set to 60 000 with a target value of 1 × 10^5^ ions and a maximum injection time of 100 ms. Fixed first mass was set to 110 *m*/*z* and the normalized collision energy was set to 32% for HCD. Dynamic exclusion was set to 30 s, peptide match was set to preferred, and precursors with unknown charge or a charge state of 1 and ≥8 were excluded.

### Proteomics Data Analyses

Raw data files were processed using MaxQuant v1.6.12.0, set to “reporter ion MS2” with “10plex TMT.” Peak lists were searched against a reviewed Uniprot human database (downloaded Feb 2020 containing 20350 sequences), appended with *EWS–FLI1* sequences and a common contaminants database, using Andromeda within MaxQuant. All fractions were searched with up to three missed trypsin cleavage sites, fixed carbamidomethylation (C) modification, dynamic oxidation (M), deamidation (NQ), and acetylation (N‐terminal) modifications. Peptide false discovery rate was set to 1%. Data were further analyzed in Perseus and Microsoft Excel. Each reporter ion channel was summed across all quantified proteins and mean‐normalized assuming equal protein loading of all samples.

### RNA Extraction and Real‐Time Quantitative Reverse Transcription PCR (qRT‐PCR)

Cells were isolated by dissociation with 0.05% Trypsin, followed by media quenching. The cells were spun down at 300× rcf for 5 min. The media was aspirated, the pellet was suspended with 1× PBS, and then the cells were spun down again. The PBS was aspirated. RNA extraction was performed with a RNA extraction kit (BioBasic BS584). The final elution step was done with 50 µL of RNAse‐free water. The relative enrichment of mRNA was quantified with the NanoDrop OneC (Thermo Fisher Scientific). Three biological replicates were performed for RNA extraction. Quartile analysis was done to exclude outliers and significance was determined by one‐way analysis of variance (ANOVA) tests.

### Cell Viability (MTT) Assays

2000 indicated cells were seeded in each well of 96‐well plates for MTT assays to monitor cell viability at indicated time periods using a method adapted from https://www.thermofisher.com/us/en/home/references/protocols/cell‐culture/mtt‐assay‐protocol/vybrant‐mtt‐cell‐proliferation‐assay‐kit.html. Briefly, at indicated time points post‐cell seeding, 10 µL MTT solution was added into each well and incubated in the culture incubator (37 °C with 5% CO_2_) for 4 h. Then, medium was removed and 100 µL dimethyl sulfoxide (DMSO) was added into each well to dissolve the formazan crystal and incubated for 10 min at 37 °C. After thorough mixing, absorbance at 540 nm was measured using the BioTek Cytation 5 Cell Imaging reader.

### Colony Formation Assays

Indicated cells were seeded into 6‐well plates (300 or 600 cells per well) or 6 cm dishes (500 or 1000 cells per dish) and cultured in 37 °C incubator with 5% CO_2_ for ≈14 days (as indicated in figure legends) until formation of visible colonies. 7Ai was refresed every other day. Colonies were washed with 1× PBS and fixed with 10% acetic acid/10% methanol for 30 min, stained with 20% acidic acid/10% methanol with 0.1% crystal violet until colonies were visibly stained. Colonies were then washed by tap water and air‐dried. Colony numbers were manually counted. At least two independent experiments were performed to generate the error bars.

### Soft Agar Assays

The anchorage‐independent cell growth assays were performed as described previously.^[^ ^53^ ^]^ Briefly, the assays were preformed using 6‐well plates where the solid medium consisted of two layers. The bottom layer contained 0.8% noble agar and the top layer contained 0.4% agar suspended with 3 × 10^4^ or indicated number of cells. 500 µL complete DMEM medium with 10% FBS was added every 4 days. About 4 weeks later, the cells were stained with iodonitrotetrazolium chloride (1 mg mL^−1^) (Sigma I10406) overnight for colony visualization and counting. At least two independent experiments were performed to generate the error bar.

### Mouse Xenograft Assays

All mouse work was reviewed and approved by UNC Institutional Animal Care and Use Committee under IACUC#19‐031. Mouse xenograft assays were performed as described previously.^[^ ^53^, ^54^ ^]^ Briefly, for mouse xenograft experiments, 2.5 × 10^6^ parental or *EWS–FLI1*‐*3A* knock‐in A673 cells, or A673/MHH‐ES‐1 teton‐shOTUD7A cellsas indicated were mixed 1:1 with matrigel (Corning 354230) and injected into the flank of indicated female nude mice (NCRNU‐M‐M from UNC Animal Facility, 4 weeks old). Six days postinjection, when the tumors were established, 2% sucrose with or without 1 µg mL^−1^ tetracycline was supplied in water for mice and refreshed every 5 days. Tumor size was measured every two days with a digital caliper, and the tumor volume was determined with the formula: *L* × *W* ^2^ × 0.52, where *L* is the longest diameter and *W* is the shortest diameter. After 22 days, mice were sacrificed, and tumors were dissected and weighed.

For 7Ai treatment in nude mice, 2.5 × 10^6^ A673 cells were mixed 1:1 with matrigel (Corning 354230) and injected into the flank of indicated female nude mice (NCRNU‐M‐M from UNC Animal Facility, 4 weeks old). Seven days postinjection, when the tumors were established, 100 µL 7Ai (25 mg kg^−1^, dissolved in ethanol followed by the addition of sunflower oil and then sonicated at 4 °C in a water bath sonicator until 7Ai was completely dissolved) was given to mice through IP injections and this treatment was repeated every 3 days.

### Transwell Assays

1 × 10^5^ cells were plated in an 8.0 mm, 24‐well plate chamber insert (Corning Life Sciences, catalog no. 3422) with serum free DMEM medium at the top of the insert and the same medium containing 20% FBS at the bottom of the insert. Cells were incubated for 24 h and fixed with 4% paraformaldehyde for 15 min. After washing with PBS, cells at the top of the insert were scraped with a cotton swab. Cells adherent to the bottom were stained with 0.5% crystal violet blue for 60 min and then washed with double‐distilled H_2_O. The positively stained cells were examined under the microscope.

### IHC

Freshly dissected xenografted tumors were immediately fixed in 10% formalin for 2 days before transferring to 80% ethanol for one day to prepare tumor blocks. 4 µm sections were cut from each of the tumor blocks by UNC TPL facility and used for IHC study. IHC was performed as described previously.^[^ ^53^ ^]^ Normal human tissue TMA was purchased from UNC TPL facility. Human Ewing sarcoma tissues were obtained postmortem and fixed in 10% formalin and embedded in paraffin. This protocol was approved by UNC Office of Human Research Ethics under institutional review board (IRB): 20‐3178.

### ITC

ITC measurements were performed using a MicroCal auto‐iTC200 calorimeter (MicroCal, LLC) as described previously.^[^ ^61^ ^]^ Briefly, His‐tagged *OTU* domain of *OTUD7A* proteins were purified using BL21 strain upon induced by 0.3 × 10^−3^ m isopropyl β‐d‐1‐thiogalactopyranoside (IPTG) when OD600 around 0.6 overnight at 16 °C. Then, purified human His‐tagged *OTU* domain of *OTUD7A* was dialyzed against 50 × 10^−3^ m HEPES buffer (pH 7.2) with 100 × 10^−3^ m NaCl for overnight at 4 °C. The concentration of the protein was determined by band intensity in a gel‐cod staining SDS‐PAGE gel. The ITC assay was carried out at 37 °C. The dialyzed His‐*OTUD7A*‐*OTU* proteins were diluted to 40 × 10^−6^ m in the dialysis buffer containing 4% DMSO. Then, 400 × 10^−6^ m 7Ai, dissolved in the same dialysis buffer with 4% DMSO (50 × 10^−3^ m HEPES buffer (pH 7.2) with 100 × 10^−3^ m NaCl) was injected into 0.4 mL of His‐*OTUD7A*‐*OTU* protein in the chamber for every 180 s. The dissociation constants and thermodynamic parameters were determined by using the embedded software package Origin7 (Microcal).

### *OTUD7A OTU* Domain Homology Model

A homology model for *OTUD7A* was built on the homologous protein *OTUD7B*, for which the catalytic *OTU* domain was crystallized both in complex with a diubiquitin, monoubiquitin, and as an apo structure (PDB codes 5LRV, 5LRW, 5LRU, respectively.^[^ ^62^ ^]^ The paper reported three sites of interest, the distal (S1) and proximal (S1ʹ) ubiquitin‐binding sites, as well as the catalytic center. Substrate recruitment was shown to be primarily driven by the S1 site. In the interest of selectivity, it was desirable to identify a small molecule binding site that was different in the two proteins. In *OTUD7B*, both S1ʹ and the catalytic site underwent significant conformational changes upon substrate binding. Furthermore, the region around the catalytic site was highly conserved between the two homologs. S1, on the other hand, had a binding pocket formed in part by two alpha helices connected by a loop that appeared suitable for small molecule inhibitors and showed relatively small changes between the three different *OTUD7B* crystal structures. In *OTUD7A*, this loop (Q257‐W263) was shorter, suggesting a different shape and smaller size of S1 in the *OTUD7A*, providing for potential ligand selectivity.

Based on these observations, the S1 region of the *OTUD7A* model was selected for the virtual screening campaign. The monoubiquitin‐bound *OTUD7B* crystal structure 5LRW (after removal of the monoubiquitin) seemed the best suited as a modeling template, as it was both in a ligand‐bound conformation and had density for all the residues surrounding the area of interest. The homology model of *OTUD7A* was built using ICM Pro v3.8.7 (Molsoft L.L.C.). Like in the experimental *OTUD7B* structures, the long, unstructured V‐loop (residues 276–300) was replaced by QPG.

### AI‐Based Small Molecule Virtual Screen

The virtual screen was carried out using the AtomNet neural network, the first deep convolutional neural network for structure‐based drug design.^[^ ^47^, ^48^ ^]^ A single global AtomNet model was deployed to predict binding affinity of small molecules to a target protein. The model was trained with experimental *K*_i_, *K*_d_, and the half maximal inhibitory concentration (IC50) values of several million small molecules and protein structures spanning several thousand different proteins, curated from both public databases and proprietary sources. Because AtomNet was a global model, it could be applied to novel binding sites with no known ligands, a prerequisite to most target‐specific machine‐learning models. Another advantage of using a single global model in prospective predictions was that it helped prevent the so‐called model overfitting. The following three‐step procedure was applied to train AtomNet models. The first step was to define the binding site on a given protein structure using a flooding algorithm^[^ ^63^ ^]^ based on an initial seed. The initial starting point of the flooding algorithm might be determined using either a bound ligand annotated in the PDB database or crucial residues as revealed by mutagenesis studies, or identification of catalytic motifs previously reported. The second step was to shift the coordinates of the protein–ligand cocomplex to a 3D Cartesian system with an origin at the center‐of‐mass of the binding site. In order to prevent the neural network from memorizing a preferred orientation of the protein structure, data augmentation was then performed by randomly rotating and translating the protein structure around the center‐of‐mass of the binding site. The third step was to sample the conformations or poses of a small molecule ligand within the binding site pocket. For a given ligand, an ensemble of poses were generated, and each of these poses represented a putative cocomplex with the protein. Each generated cocomplex was then rasterized into a fixed‐size regular 3D grid, where the values at each grid point represented the structural features that were present at each point. Similar to a photo pixel containing three separate channels representing the presence of red, green, and blue colors, the grid points represented the presence of different atom types. These grids served as the input to a convolutional neural network, and defined the receptive field of the network. A network architecture of a 30 × 30 × 30 grid with 1 Å spacing was used for the input layer, followed by five convolutional layers of 32 × 3^3^, 64 × 3^3^, 64 × 3^3^, 64 × 3^3^, 64 × 2^3^ (number of filters × filter dimension), and a fully connected layer with 256 ReLU hidden units. The scores for each pose in the ensemble were combined through a weighted Boltzmann averaging to produce a final score. These scores were compared against the experimentally measured p*K*_i_ or pIC50 (converted from *K*_i_ or IC50) of the protein and ligand pair, and the weights of the neural network were adjusted to reduce the error between the predicted and experimentally measured affinity using a mean‐square‐error loss function. Training was done using the ADAM^[^ ^64^ ^]^ adaptive learning method, the backpropagation algorithm, and minibatches with 64 examples per gradient step.

AtomNet could take any form of 3D protein structures determined by experimental methods including crystallography, NMR, and cryogenic electron microscopy (cryo‐EM) published in PDB format. In case of no available experimental protein structure of the target, the amino acid sequence of the target could be used to build a homology model using the most homologous protein structure as a template as described above. The binding site was identified surrounded by residues R249, W250, R251, W252, Q253, Q254, T255, Q256, Q257, K259, E261, R265, E266, W267, E269, L270, L273, E304, E305, F306, H307, P339, F340, F400 on the *OTUD7A* homology model.

The Mcule small‐molecule library version v20171018, containing 5 648 837 small organic molecules for drug discovery purchasable from the chemical vendor Mcule, was screened. The library in simplified molecular‐input line‐entry system (SMILES) format was downloaded from Mcule's website (https://mcule.com/). Every compound in the library was pushed through a standardization process including the removal of salts, isotopes, and ions, and conversion to neutral form; conversion of functional groups and aromatic rings to consistent representations. Filters were then applied on some molecular properties including molecular weight between 100 and 700 Da, total number of chiral centers in a molecule ≤ 6, total number of atoms in a molecule ≤ 60, total number of rotatable bonds ≤ 15, and only molecules containing C, N, S, H, O, P, B, halogens were allowed. Other filters such as toxicophores, Eli Lilly's MedChem Rules,^[^ ^65^ ^]^ and pan‐assay interference compounds (PAINS) were also applied to remove compounds with undesirable substructures, resulting in a filtered library of 4 025 533 compounds. For each small molecule, a set of 64 poses within the binding site was generated. Each of these poses was scored by the trained model, and the molecules were ranked by their scores. The top 5000 ranking compounds were examined from which a set of 89 compounds containing diverse chemical scaffolds was selected. The selected compounds were sourced from Mcule. Of 88 available compounds, 73 passed quality control with 62 compounds having at least 90% purity measured by LC–MS. Eleven compounds had purity between 78% and 90%. The compound 7Ai had a clean mass spectrum and was at 86.9% purity. After being identified as a hit, compound 7Ai was purified by HPLC and assayed again to confirm its activity.

### Activity Assay for 73 Predicted Compounds from AI‐Based Virtual Screen

Each compound was dissolved in DMSO with a concentration of 10 × 10^−3^ m. The compound samples were assayed in a blinded way (chemical identities unknown to the lab researcher, with two negative control samples containing pure DMSO mixed in). A673 and SK‐N‐MC cells were splitted into 6‐well plates and treated with each compound with a final compound concentration of 10 × 10^−6^ m for 12 h. Cells were harvested and subjected to western blot analyses.

### Statistical Analysis

Statistical analyses were performed using the SPSS 11.5 Statistical Software. *p* ≤ 0.05 was considered statistically significant. The results were shown as means ± standard deviation (SD) from at least two or three independent experiments as indicated in figure legends. Differences between control and experimental conditions were evaluated by one‐way ANOVA.

## Conflict of Interest

Christian Laggner and Kong T. Nguyen are current employees of Atomwise Inc.

## Author Contributions

J.C. and Y.J. are co‐first authors. S.S., J.C., and Y.J. contributed equally to this work. I.J.D. and P.L. conceived the project. S.S., J.C., C.L., K.T.N., I.J.D., and P.L. designed experiments. S.S., J.C., Y.J., Y.W., T.V., J.Z., C.L., K.T.N., Z.Z., A.W.P., N.K.B., and L.E.H. performed experiments. S.S., C.L., K.T.N., L.E.H., I.J.D., and P.L. analyzed the data. I.J.D. and P.L. supervised this study. S.S., I.J.D., and P.L. wrote the paper.

## Supporting information

Supporting Information

Supporting Information

Supporting Information

## Data Availability

The data that support the findings of this study are available from the corresponding author upon reasonable request.
