# Supplementary material for: SPOP and OTUD7A Control EWS–FLI1 Protein Stability to Govern Ewing Sarcoma Growth
Source: Adv Sci (Weinh). 2021 Jun 1;8(14):2004846. doi: 10.1002/advs.202004846 (PMC8292909; doi:10.1002/advs.202004846)
Supplement: Supplementary file 1 — Supporting Information [file ADVS-8-2004846-s001.pdf]

© 2021 Wiley-VCH GmbH

ADVANCED SCIENCE
Open Access

Supporting Information

for *Adv. Sci.*, DOI: 10.1002/advs.202004846

SPOP and OTUD7A control EWS-FLI1 protein stability to govern Ewing sarcoma growth

Siyuan Su, Jianfeng Chen, Yao Jiang, Ying Wang, Tamara Vital, Jiaming Zhang, Christian Laggner, Kong T. Nguyen, Zhichuan Zhu, Alex W. Prevatte, Natalie K. Barker, Laura E. Herring, Ian J. Davis and Pengda Liu

ADVANCED SCIENCE
Open Access

Supporting Information for

SPOP and OTUD7A control EWS-FLI1 protein stability to govern Ewing sarcoma growth

Siyuan Su, Jianfeng Chen, Yao Jiang, Ying Wang, Tamara Vital, Jiaming Zhang, Christian Laggner, Kong T. Nguyen, Zhichuan Zhu, Alex W. Prevatte, Natalie K. Barker, Laura E. Herring, Ian J. Davis and Pengda Liu

correspondence to: ian_davis@med.unc.edu and pengda_liu@med.unc.edu

This PDF file includes:

Figs. S1 to S21
Table S2: 99 characterized EWS-FLI1 target proteins with -0.5<$\log_2$ fold change (p value<0.05) upon *OTUD7A* depletion.
Tables S1 and S3 are uploaded in separate excel files due to large size.

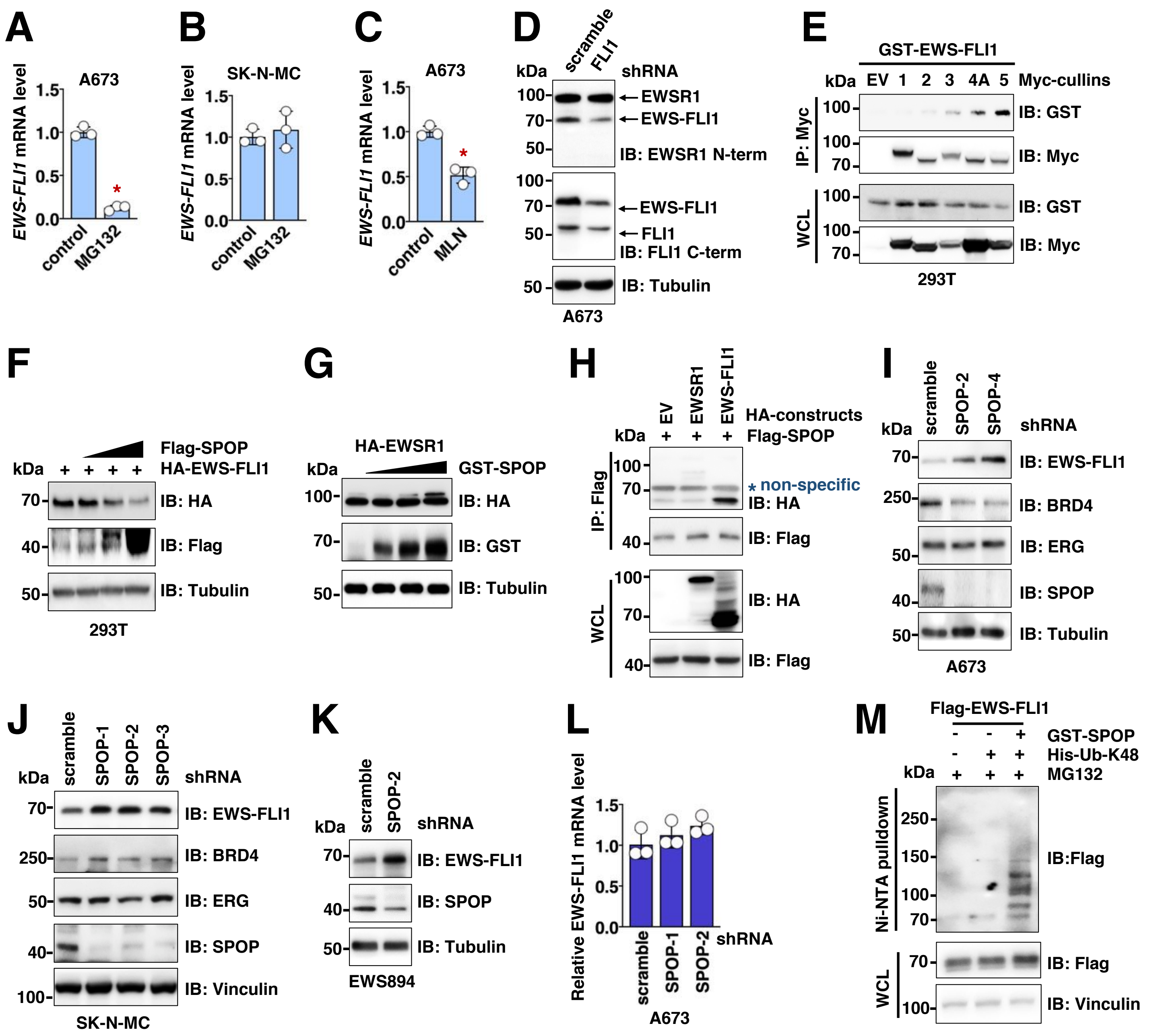

Fig. S1. SPOP targets EWS-FLI1 for ubiquitination and degradation.
(A, B, C) RT-PCR analyses of *EWS-FLI1* mRNA expression changes upon treatment by 10 μM MG132 for 4 hrs or 1 μM MLN4924 overnight in A673 (A, C) or SK-N-MC (B) cells. Error bars were calculated as mean+/-SD, n=3. *p < 0.05 (one-way *ANOVA* test).
(D) IB analyses of WCL derived from A673 cells infected with FLI1 shRNA expressing viruses and harvested 2 days post-infection.
(E) IB analyses of GST-pulldown and WCL derived from HEK293T cells transfected with indicated DNA constructs.
(F, G) IB analyses of WCL derived from HEK293T cells transfected with indicated DNA constructs.
(H) IB analyses of Flag-IP and WCL derived from HEK293T cells transfected with indicated DNA constructs.
(I, J, K) IB analyses of WCL derived from A673 (I), SK-N-MC (J) and EWS894 (K) cells depleted of endogenous *SPOP*.
(L) *EWS-FLI1* mRNA analyses by RT-PCR in indicated A673 cells. Error bars were calculated as

mean+/-SD, n=3. $p > 0.05$ (one-way *ANOVA* test) and no significance was found.
(M) IB analyses of Ni-NTA pulldown and WCL derived from HEK293T cells transfected with indicated DNA constructs. Cells were treated with 10 μM MG132 for 12 hours before cell collection.

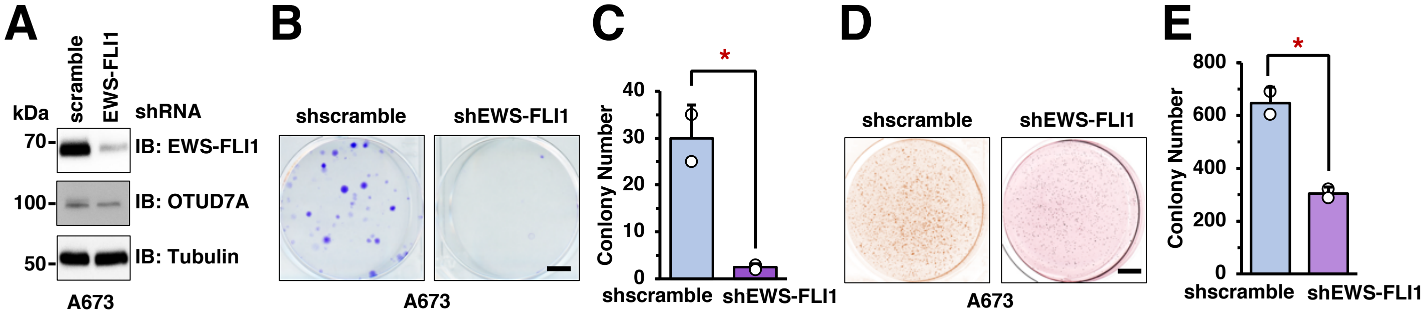

Fig. S2. Depletion of endogenous *EWS-FLI1* impedes A673 cell growth *in vitro*.

(A) IB analyses of WCL derived from A673 infected with FLI1 shRNA expressing viruses and harvested 2 days post-infection.

(B, C) Representative images for 2D colony formation assays (B) using cells from (A) and quantified in (C). Error bars were calculated as mean+/-SD, n=2. *$p < 0.05$ (one-way *ANOVA* test). The scale bar represents 10 mm.

(D, E) Representative images for 3D soft agar assays (D) using cells from (A) and quantified in (E). Error bars were calculated as mean+/-SD, n=2. *$p < 0.05$ (one-way *ANOVA* test). The scale bar represents 10 mm.

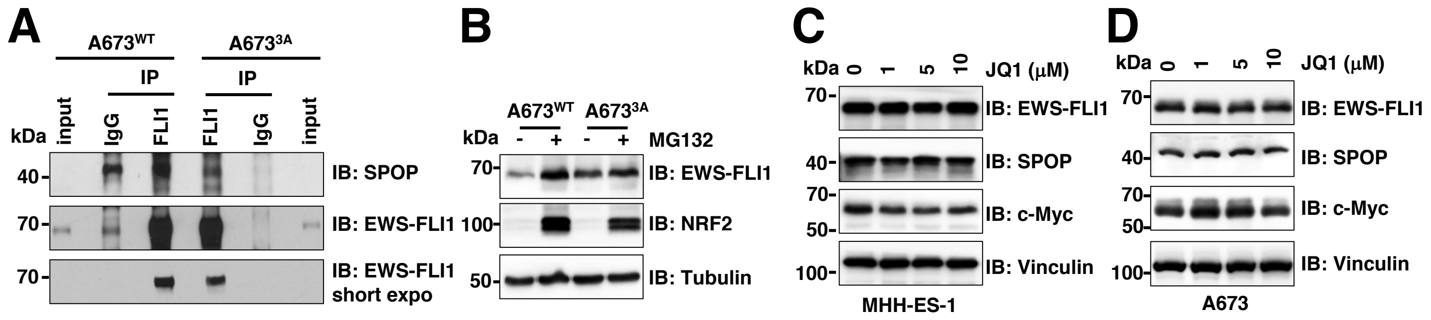

Fig. S3. EWS-FLI1 protein stability is controlled by SPOP but not BRD4.

(A) IB analyses of FLI1-IPs and WCL derived from either parental A673 and *EWS-FLI1-3A* knockin A673 cells.

(B) IB analyses of WCL derived from indicated A673 cells treated with 10 μM MG132 for 4 hours before cell collection.

(C, D) IB analyses of WCL derived from MHH-ES-1 (C) or A673 (D) cells treated with indicated doses of BRD4 inhibitor JQ1 for 24 hours before cell collection.

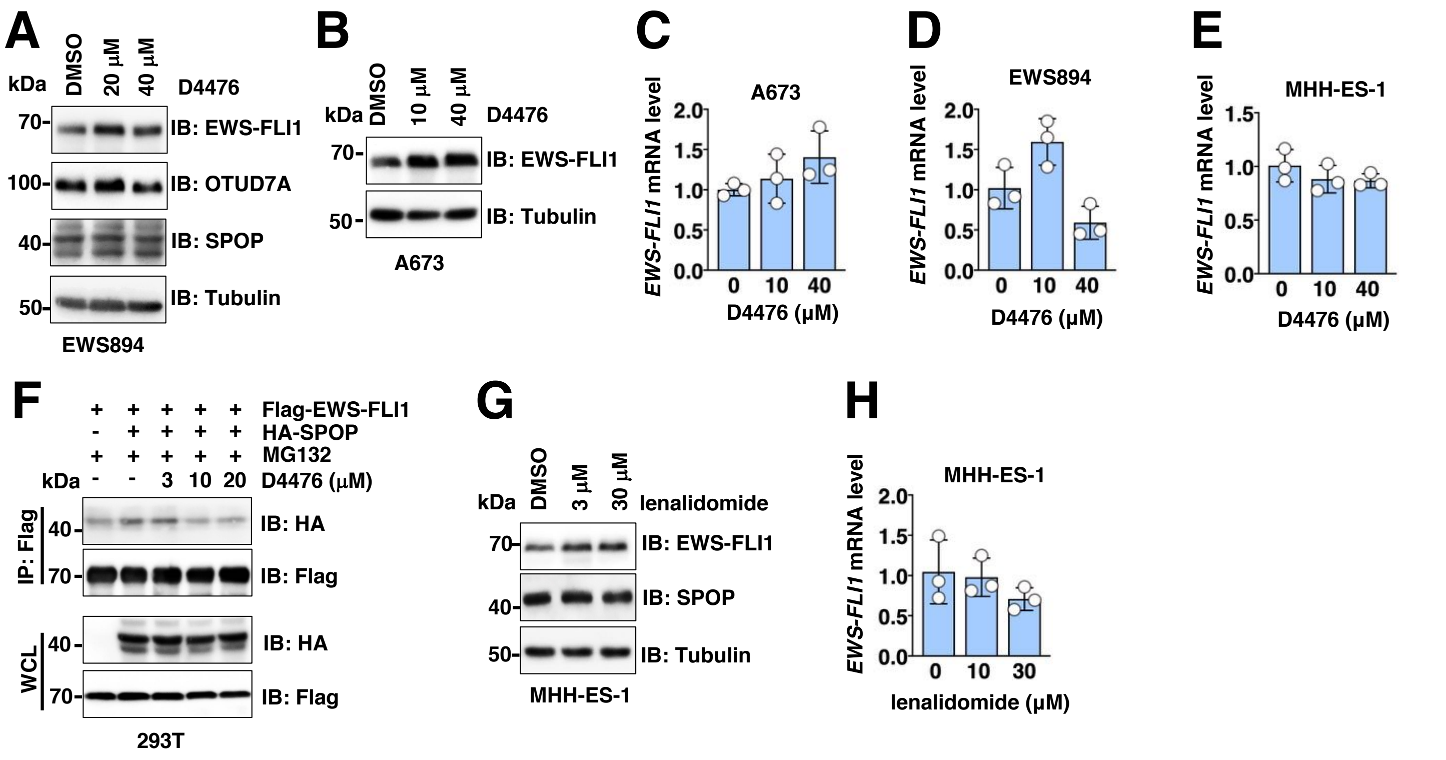

Fig. S4. CK1 inactivation stabilizes EWS-FLI1 proteins in Ewing sarcoma.
(A, B) IB analyses of WCL derived from EWS894 (A) and A673 (B) cells treated with indicated doses of D4476 for 12 hrs.
(C, D, E) RT-PCR analyses of *EWS-FLI1* mRNA changes in A673 (C), EWS894 (D) or MHH-ES-1 (E) cells treated with indicated doses of D4476 for 12 hours before cell collection. Error bars were calculated as mean+/-SD, n=3. $p > 0.05$ (one-way *ANOVA* test) and no statistical significance was observed.
(F) IB analyses of Flag-IP and WCL derived from HEK293 cells transfected with indicated DNA constructs.
(G) IB analyses of WCL derived from MHH-ES-1 cells treated with indicated doses of lenalidomide for 12 hours before cell collection.
(H) RT-PCR analyses of *EWS-FLI1* mRNA changes derived from MHH-ES-1 cells treated with indicated doses of lenalidomide for 12 hours before cell collection. Error bars were calculated as mean+/-SD, n=3. $p > 0.05$ (one-way *ANOVA* test) and no statistical significance was observed.

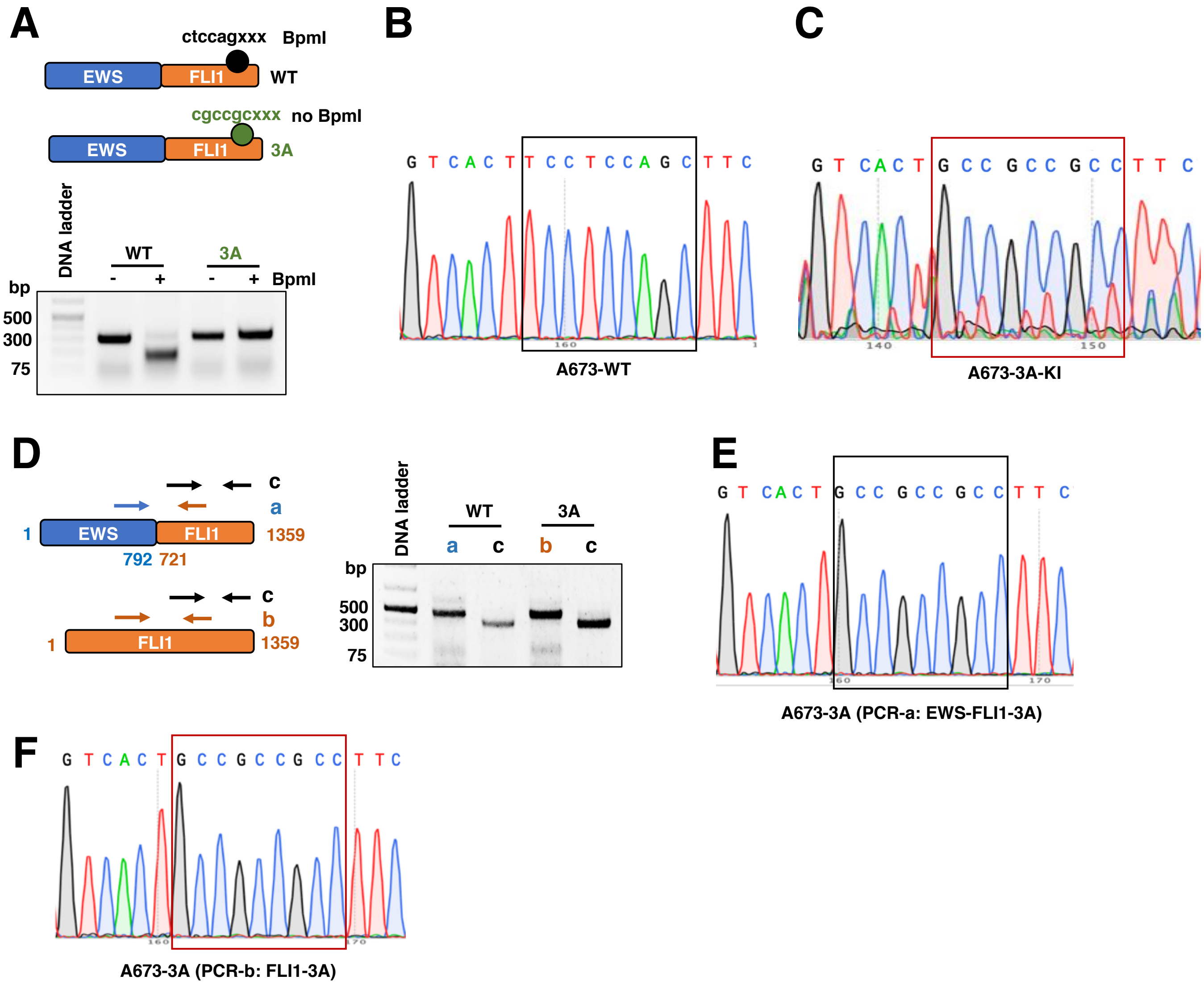

Fig. S5. Validation of *EWS-FLI1-3A* knockin A673 clones.

(A) Top, a cartoon illustration of nucleotide changes upon *EWS-FLI1-3A* knockin. Bottom, BpmI digestion analysis of PCR products derived from genomic DNA extracted from either parental or *EWS-FLI1-3A knockin* A673 cells.

(B, C) Representative sanger sequencing results from either parental A673 (B) or *EWS-FLI1-3A* knockin A673 cells (C).

(D) Left, a cartoon illustration of primer pairs designed specific to either *EWS-FLI1* fusion (a) or *FLI1-WT* (b), or both (c). Right, PCR analysis of PCR products derived from genomic DNA extracted from either parental or *EWS-FLI1-3A* knockin A673 cells.

(E, F) Representative sanger sequencing results from *EWS-FLI1-3A* knockin A673 cells by either PCR primer pair a (E) or b (F).

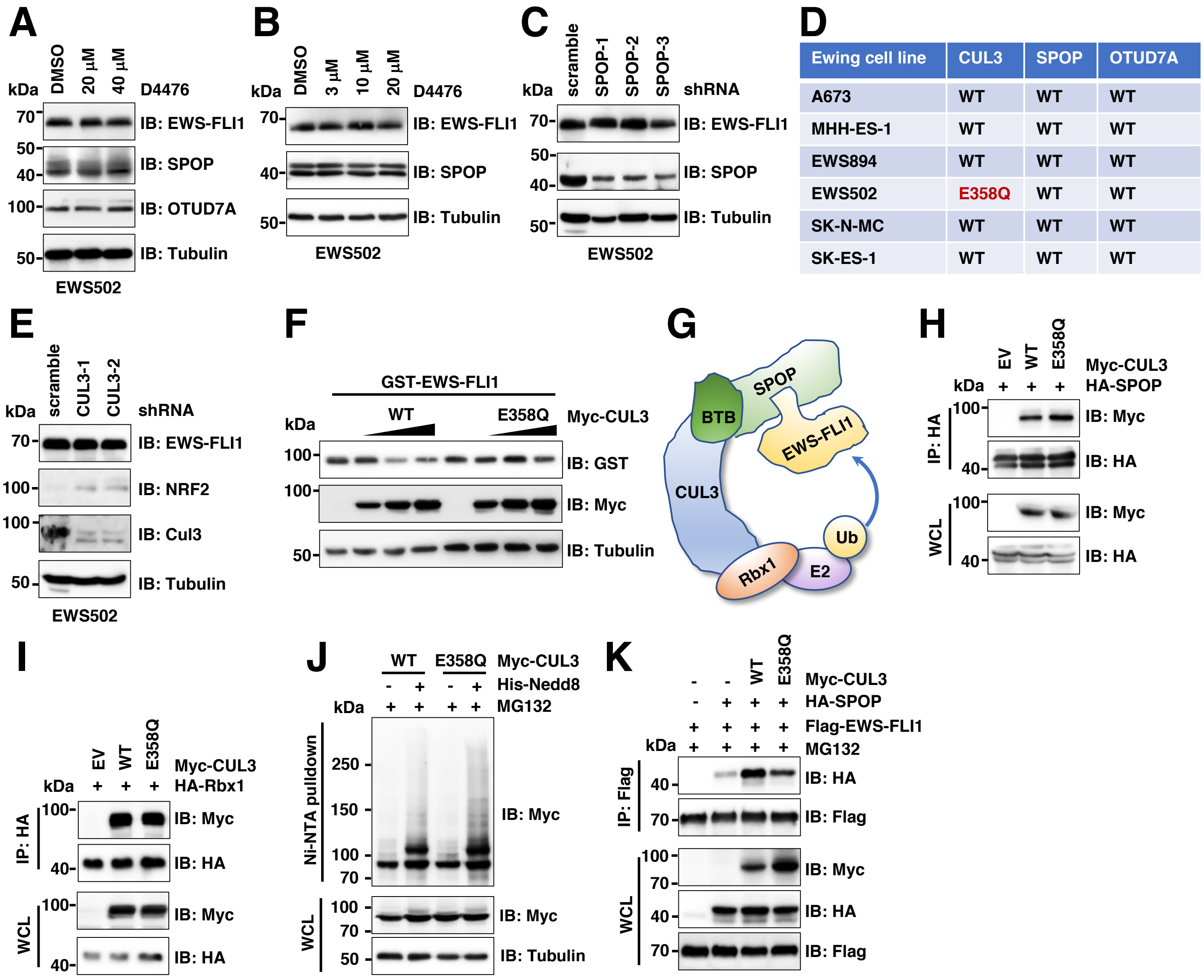

Fig. S6. The cullin 3-E358Q mutant is deficient in targeting EWS-FLI1 for degradation.
(A, B) IB analyses of WCL derived from EWS502 cells treated with indicated doses of D4476 for 16 hrs.
(C) IB analyses of WCL derived from EWS502 cells infected with indicated *shSPOP* expressing lentiviruses and selected for 72 hrs with 1 µg/mL puromycin to eliminate non-infected cells.
(D) Summary of CUL3, SPOP and OTUD7A genetic status in indicated Ewing sarcoma cell lines. Information was obtained from DEPMAP portal.
(E) IB analyses of WCL derived from EWS502 cells infected with indicated shCUL3 expressing lentiviruses and selected for 72 hrs with 1 µg/mL puromycin to eliminate non-infected cells.
(F) IB analyses WCL derived from HEK293T cells transfected with indicated DNA constructs.
(G) A cartoon illustration of SPOP/CUL3 E3 ligase complex composition.
(H, I) IB analyses of HA-IP and WCL derived from HEK293T cells transfected with indicated DNA constructs.
(J) IB analyses of Ni-NTA pulldowns and WCL derived from HEK293T cells transfected with indicated DNA constructs. Cells were treated with 10 µM MG132 overnight before cell collection.
(K) IB analyses of Flag-IP and WCL derived from HEK293T cells transfected with indicated DNA constructs. Cells were treated with 10 µM MG132 overnight before cell collection.

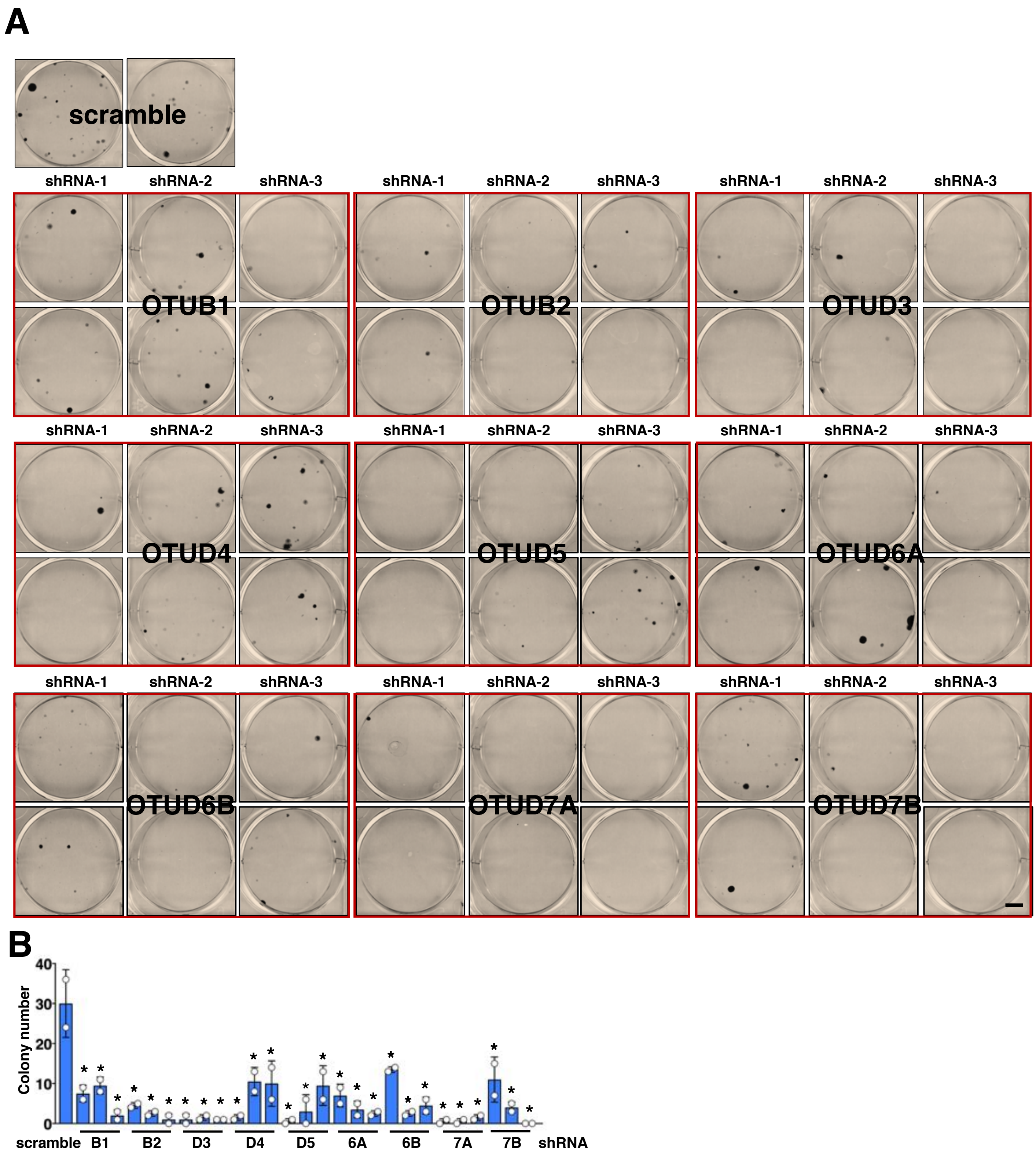

Fig. S7. Depletion of *OTUD7A* leads to reduced A673 cell growth *in vitro*.

(A) Representative images for *shOTU*s-mediated colony formation assays in A673 cells with quantifications in (B). Error bars were calculated as mean+/-SD, n=2. *p<0.05 (one-way *ANOVA* test). The scale bar represents ~6 mm.

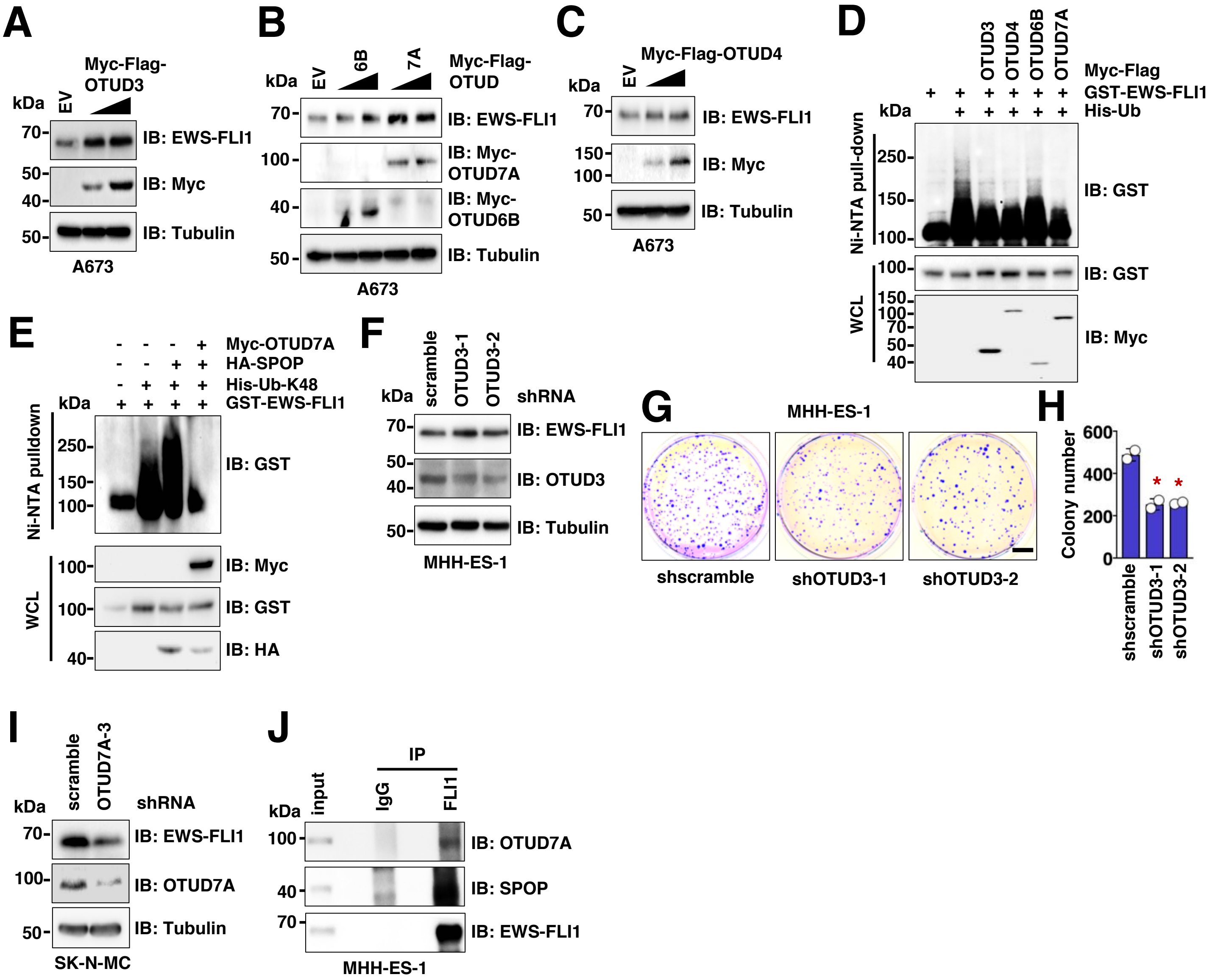

Fig. S8. Depletion of *OTUD7A* leads to reduced EWS-FLI1 protein abundance in Ewing sarcoma cells and subsequently impeded Ewing sarcoma growth.
(A, B, C) IB analyses of WCL derived from A673 cells transfected with indicated DNA constructs.
(D, E) IB analyses of Ni-NTA pulldown and WCL derived from HEK293T cells transfected with indicated DNA constructs.
(F) IB analyses of WCL derived from MHH-ES-1 cells infected with indicated shOTUD3 expressing lenti-viruses and selected for 72 hrs with 1 μg/mL puromycin to eliminate non-infected cells.
(G, H) Representative colony formation images using cells obtained from (F) with quantifications in (H). Error bars were calculated as mean+/-SD, n=2. *$p<0.05$ (one-way *ANOVA* test). The scale bar represents 10 mm.
(I) IB analyses of WCL derived from SK-N-MC cells infected with indicated shOTUD7A expressing lenti-viruses and selected for 72 hrs with 1 μg/mL puromycin to eliminate non-infected cells.
(J) IB analyses of FLI1-IP and WCL derived from MHH-ES-1 cells.

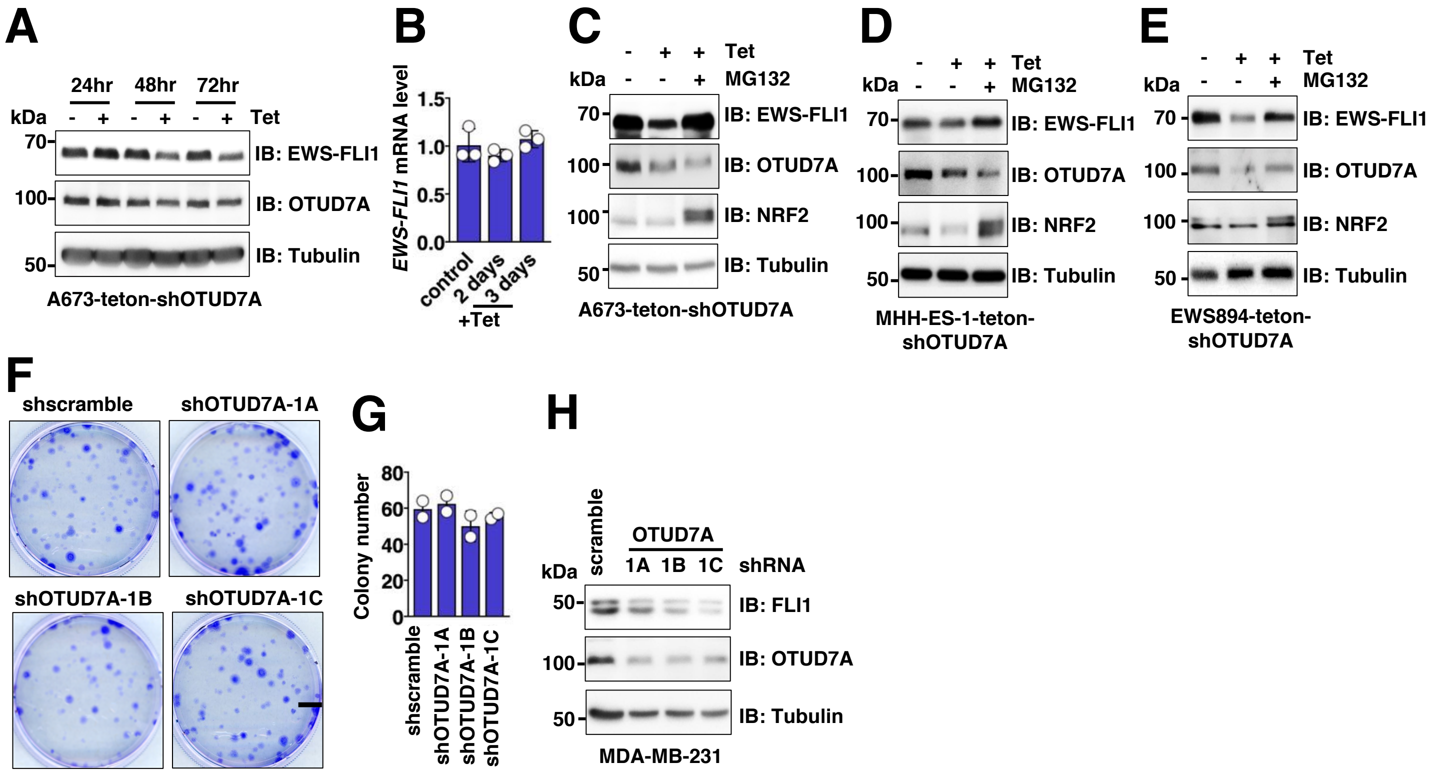

Fig. S9. Depletion of *OTUD7A* leads to impeded Ewing sarcoma growth.

(A) IB analyses of WCL derived from A673 cells expressing a teton-shOTUD7A construct. Where indicated, 1 μg/mL tetracycline was added into cell culture and cells were collected at indicated time periods post-tet addition.

(B) RT-PCR analyses of EWS-FLI1 mRNA levels upon *OTUD7A* depletion in A673 cells. Error bars were calculated as mean+/-SD, n=3. p>0.05 (one-way *ANOVA* test) and no statistical significance was observed.

(C, D, E) IB analyses of WCL derived from indicated tet-inducible OTUD7A depletion cells including A673 (C), MHH-ES-1 (D) and EWS894 (E). Where indicated, 1 μg/mL tetracycline was added into cell culture for 3 days, or 10 μM MG132 overnight before cell collection.

(F, G) Representative images of colony formation assays (F) using MDA-MB-231 cells depleted of endogenous *OTUD7A* obtained in (H) and quantified in (G). Error bars were calculated as mean+/-SD, n=2. p>0.05 (one-way *ANOVA* test) and no statistical significance was observed. The scale bar represents 10 mm.

(H) IB analyses of WCL derived from MDA-MB-231 cells infected with indicated shOTUD7A expressing lenti-viruses and selected for 72 hrs with 1 μg/ml puromycin to eliminate non-infected cells.

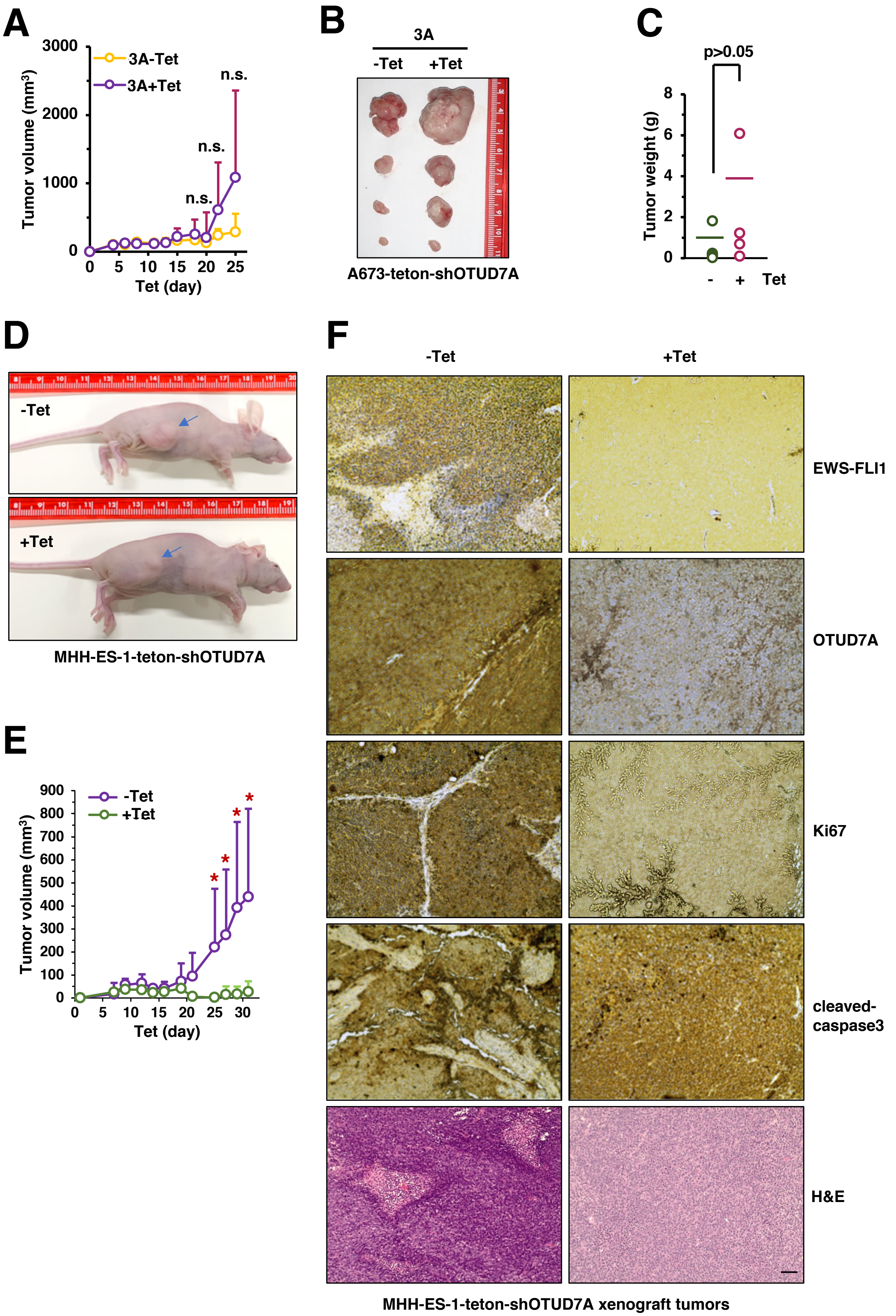
A
3000
2000
1000
0
Tumor volume (mm³)
3A-Tet
3A+Tet
n.s.
n.s.
n.s.
0 5 10 15 20 25
Tet (day)
B
3A
-Tet
+Tet
A673-teton-shOTUD7A
C
p>0.05
8
6
4
2
0
Tumor weight (g)
- + Tet
D
-Tet
+Tet
MHH-ES-1-teton-shOTUD7A
E
900
800
700
600
500
400
300
200
100
0
Tumor volume (mm³)
-Tet
+Tet
*
*
*
*
0 5 10 15 20 25 30
Tet (day)
F
-Tet
+Tet
EWS-FLI1
OTUD7A
Ki67
cleaved-caspase3
H&E
MHH-ES-1-teton-shOTUD7A xenograft tumors

Fig. S10. Depletion of *OTUD7A* leads to impeded Ewing sarcoma growth in mice.
(A, B, C) Mouse xenograft experiments were performed with indicated $A673^{3A}$ cells. 5-day post-injection when tumors were established in mice, tetracycline dissolved in water with 2% sucrose, or only 2% sucrose was fed to mice. Tumor volumes were monitored by caliper measurements at indicated days (A). 25 days post-injection, mice were sacrificed, and tumors were dissected (B) and weighed (C). Error bars were calculated as mean+/-SD, n=4. $p>0.05$ (one-way *ANOVA* test) and no statistical significance was observed.
(D, E, F) Mouse xenograft experiments were performed with indicated MHH-ES-1-teton-shOTUD7A cells. 7-day post-injection when tumors were established in mice, tetracycline dissolved in water with 2% sucrose, or only 2% sucrose was fed to mice. Tumor volumes were monitored by caliper measurements at indicated days (E). 30 days post-injection, mice were sacrificed, and tumors were dissected for histological analyses by indicated antibodies (F). Error bars were calculated as mean+/-SD, n=10. *$p<0.05$ (one-way *ANOVA* test). The scale bar represents 25 μm.

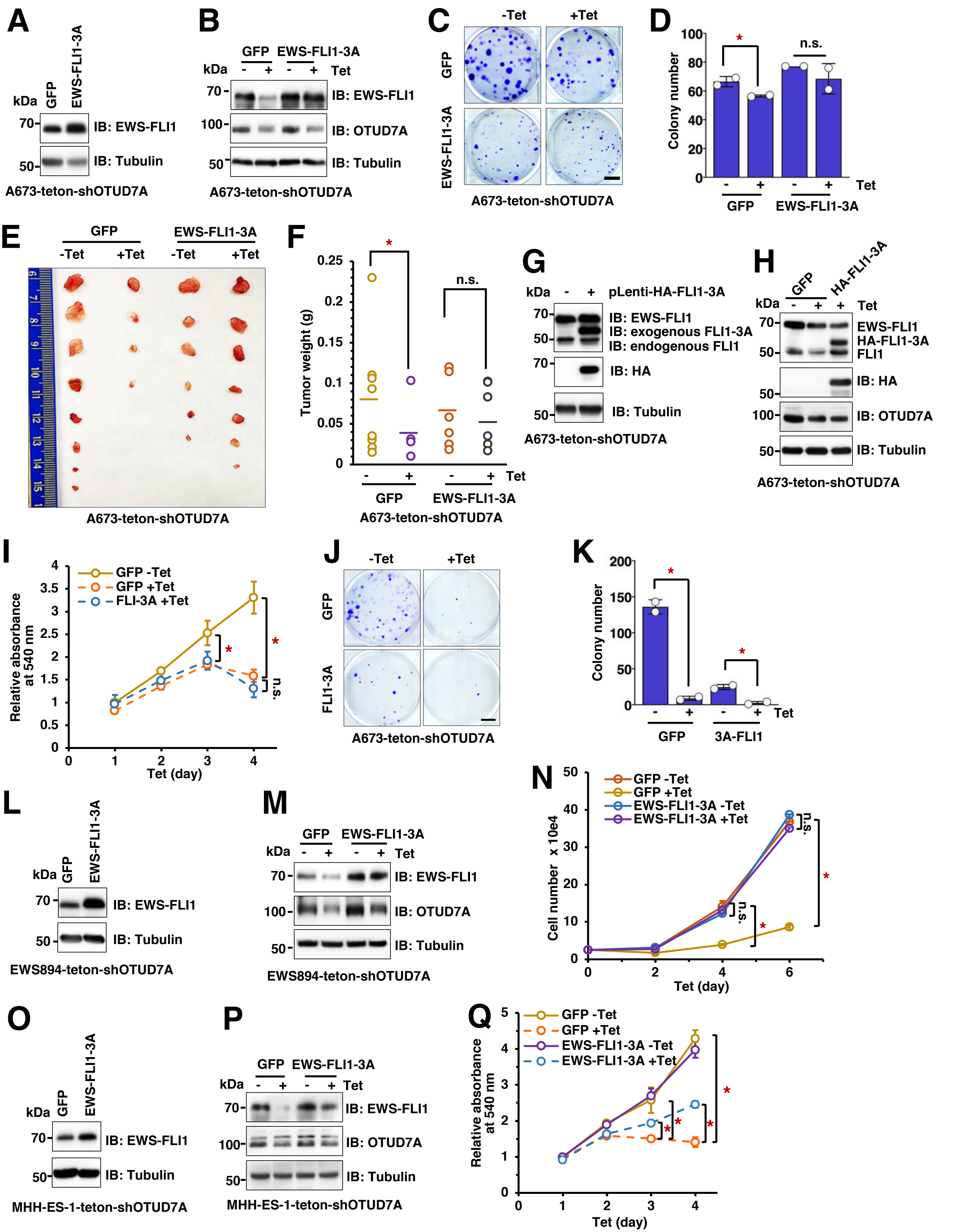
A
kDa
GFP
EWS-FLI1-3A
IB: EWS-FLI1
IB: Tubulin
A673-teton-shOTUD7A
B
GFP
EWS-FLI1-3A
Tet
IB: EWS-FLI1
IB: OTUD7A
IB: Tubulin
A673-teton-shOTUD7A
C
-Tet
+Tet
GFP
EWS-FLI1-3A
A673-teton-shOTUD7A
D
Colony number
n.s.
Tet
GFP
EWS-FLI1-3A
E
GFP
EWS-FLI1-3A
-Tet
+Tet
A673-teton-shOTUD7A
F
Tumor weight (g)
n.s.
Tet
GFP
EWS-FLI1-3A
A673-teton-shOTUD7A
G
pLenti-HA-FLI1-3A
IB: EWS-FLI1
IB: exogenous FLI1-3A
IB: endogenous FLI1
IB: HA
IB: Tubulin
A673-teton-shOTUD7A
H
GFP
HA-FLI1-3A
Tet
EWS-FLI1
HA-FLI1-3A
FLI1
IB: HA
IB: OTUD7A
IB: Tubulin
A673-teton-shOTUD7A
I
GFP -Tet
GFP +Tet
FLI-3A +Tet
Relative absorbance at 540 nm
n.s.
Tet (day)
J
-Tet
+Tet
GFP
FLI1-3A
A673-teton-shOTUD7A
K
Colony number
Tet
GFP
3A-FLI1
L
kDa
GFP
EWS-FLI1-3A
IB: EWS-FLI1
IB: Tubulin
EWS894-teton-shOTUD7A
M
GFP
EWS-FLI1-3A
Tet
IB: EWS-FLI1
IB: OTUD7A
IB: Tubulin
EWS894-teton-shOTUD7A
N
GFP -Tet
GFP +Tet
EWS-FLI1-3A -Tet
EWS-FLI1-3A +Tet
Cell number x 10e4
n.s.
Tet (day)
O
kDa
GFP
EWS-FLI1-3A
IB: EWS-FLI1
IB: Tubulin
MHH-ES-1-teton-shOTUD7A
P
GFP
EWS-FLI1-3A
Tet
IB: EWS-FLI1
IB: OTUD7A
IB: Tubulin
MHH-ES-1-teton-shOTUD7A
Q
GFP -Tet
GFP +Tet
EWS-FLI1-3A -Tet
EWS-FLI1-3A +Tet
Relative absorbance at 540 nm
Tet (day)

Fig. S11. EWS-FLI1-3A, but not FLI1-3A expression could rescue *OTUD7A* depletion induced growth retardation.

(A) IB analyses of WCL derived from A673-teton-shOTUD7A cells expressing GFP or EWS-FLI1-3A by lentiviral infection.

(B) IB analyses of WCL derived from A673-teton-shOTUD7A cells expressing a GFP or EWS-FLI1-3A. Where indicated, 1 μg/mL tetracycline was added into cell culture and cells were collected 3 days post-tet addition.

(C, D) Representative images of colony formation assays (C) using A673 cells obtained in (A) and quantified in (D). Where indicated, 1 μg/mL tetracycline was refreshed in cell culture every 3 days and colonies were stained 25 days post-inoculation. Error bars were calculated as mean+/-SD, n=2. $^*P < 0.05$ (one-way *ANOVA* test). The scale bar represents 5 mm.

(E, F) Mouse xenograft experiments were performed with A673-teton-shOTUD7A cells expressing GFP or EWS-FLI1-3A. 7-day post-injection when tumors were established in mice, tetracycline dissolved in water with 2% sucrose was fed to mice. 26 days post-injection, mice were sacrificed, and tumors were dissected (E) and weighed (F). Error bars were calculated as mean+/-SD, n as indicated. $^*P < 0.05$ (one-way *ANOVA* test).

(G) IB analyses of WCL derived from A673-teton-shOTUD7A cells expressing GFP or FLI1-3A by lentiviral infection.

(H) IB analyses of WCL derived from MHH-ES-1-teton-shOTUD7A cells expressing a GFP or EWS-FLI1-3A. Where indicated, 1 μg/mL tetracycline was added into cell culture and cells were collected 3 days post-tet addition.

(I) Representative cell viability assays using cells obtained in (H). 2,000 cells were plated into each well in 96-well plates and 1 μg/mL tetracycline was added into cell culture and cell viability was determined at indicated periods post-tet addition. Error bars were calculated as mean+/-SD, n=5. $^*P < 0.05$ (one-way *ANOVA* test).

(J, K) Representative images of colony formation assays (J) using A673 cells obtained in (G) and quantified in (K). Where indicated, 1 μg/mL tetracycline was refreshed in cell culture every 3 days and colonies were stained 25 days post-inoculation. Error bars were calculated as mean+/-SD, n=2. $^*P < 0.05$ (one-way *ANOVA* test). The scale bar represents 5 mm.

(L) IB analyses of WCL derived from EWS894-teton-shOTUD7A cells expressing GFP or FLI1-3A by lentiviral infection.

(M) IB analyses of WCL derived from EWS894-teton-shOTUD7A cells expressing a GFP or EWS-FLI1-3A. Where indicated, 1 μg/mL tetracycline was added into cell culture and cells were collected 3 days post-dox addition.

(N) Representative cell viability assays using cells obtained in (L). 25,000 cells were cultured in each well in 6-well plate, and cells were treated with or without 1 μg/mL tetracycline as indicated. Cell numbers were measured 2, 4 and 6 days post-tet addition. Error bars were calculated as mean+/-SD, n=2. $^*P < 0.05$ (one-way *ANOVA* test).

(O) IB analyses of WCL derived from MHH-ES-1-teton-shOTUD7A cells expressing GFP or EWS-FLI1-3A by lentiviral infection.

(P) IB analyses of WCL derived from MHH-ES-1-teton-shOTUD7A cells expressing a GFP or EWS-FLI1-3A. Where indicated, 1 μg/mL tetracycline was added into cell culture and cells were collected 3 days post-dox addition.

(Q) Representative cell viability assays using cells obtained in (O). 2,000 cells were plated into each well in 96-well plates and 1 μg/mL tetracycline was added into cell culture. Cell viability was determined at indicated periods post-tet addition. Error bars were calculated as mean+/-SD, n=5. $^*P < 0.05$ (one-way *ANOVA* test).

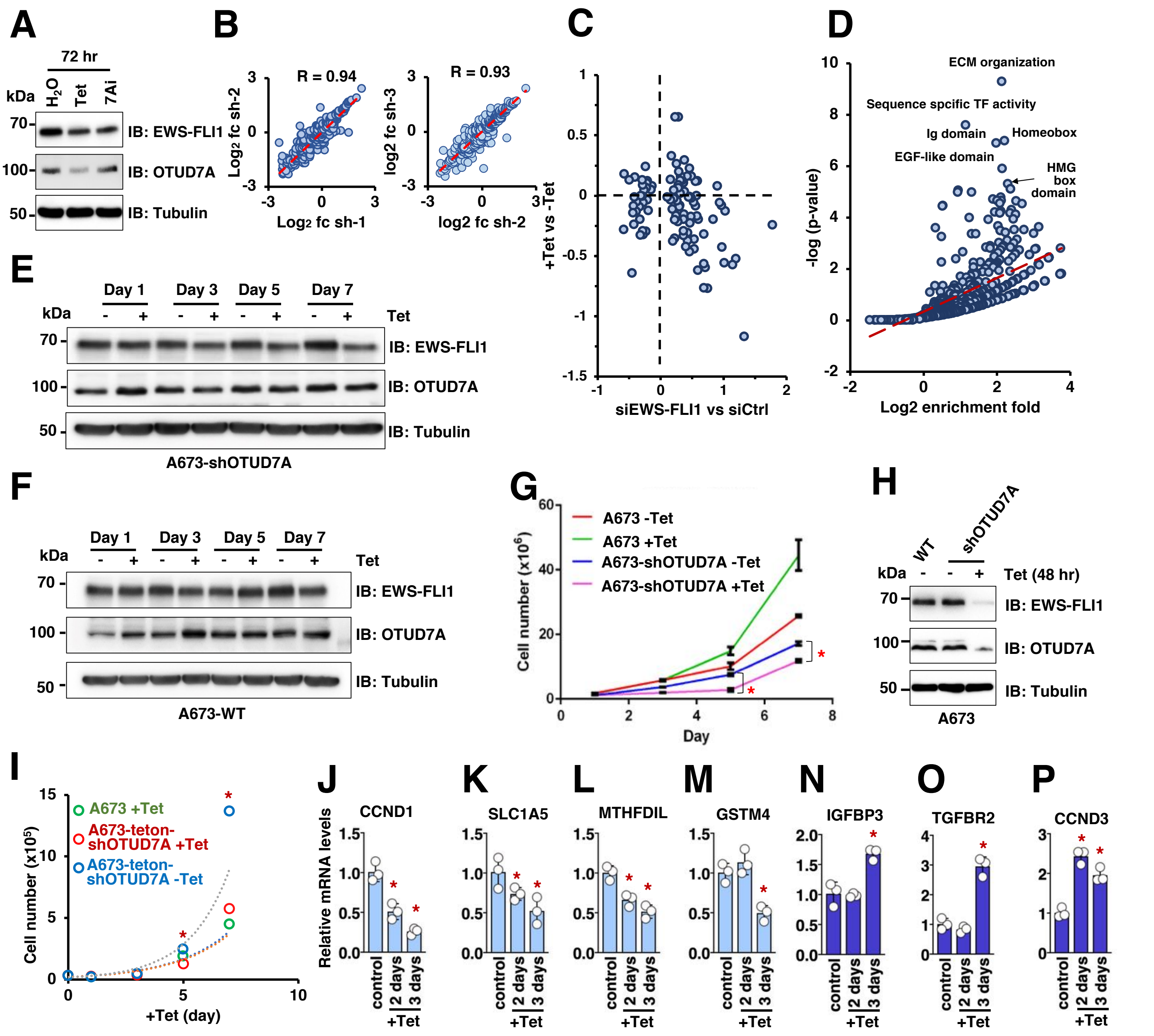

Fig. S12. Inactivation of OTUD7A attenuates EWS-FLI1 downstream signaling.

(A) IB analyses of WCL derived from A673 cells treated with water, 1 μg/mL tetracycline for 3 days or 10 μM 7Ai for 3 days.

(B) Scatter plots indicating the reproducibility of mass spectrometry analyses of cells in (A).

(C) Scatter plots indicating the reproducibility of our mass spectrometry analyses with a previously reported proteomic study.[1]

(D) DAVID analyses of biological function for down-regulated proteins upon Tet-induced *OTUD7A* depletion.

(E, F) IB analyses of WCL derived from indicated cells. Where indicated, 1 μg/mL tetracycline was added into cell culture and cells were collected at indicated time periods.

(G, I) Growth curve using either parental A673 cells or A673-teton-shOTUD7A cells treated with 1 μg/mL tetracycline for indicated days. Error bars were calculated as mean+/-SD, n=3. *P < 0.05 (one-way *ANOVA* test).

(H) IB analyses of WCL derived from indicated cells. Where indicated, 1 μg/mL tetracycline was added

into cell culture for 48 hrs.
(J, K, L, M, N, O, P) RT-PCR analyses of mRNAs derived from A673 cells expressing a Tet-inducible shOTUD7A construct treated with 1 μg/mL tetracycline for indicated periods. Error bars were calculated as mean+/-SD, n=3. *$p<0.05$ (one-way *ANOVA* test).

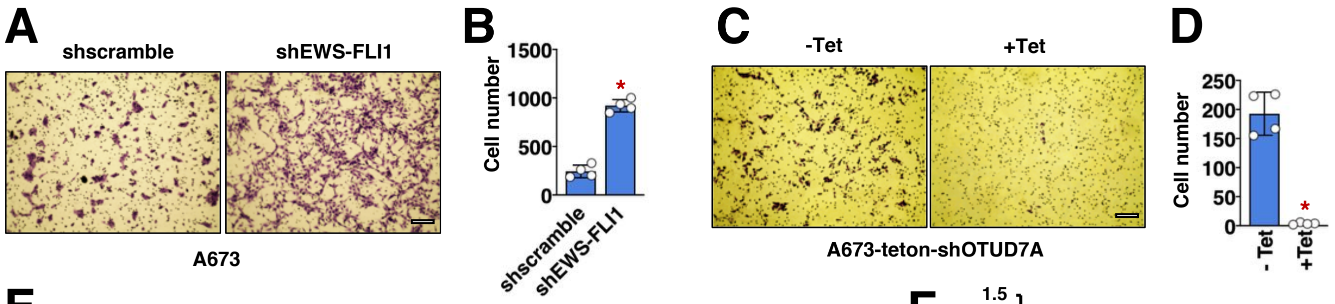

E

Integrin protein abundance changes in response to shOTUD7A and siEWS-FLI1				
ID	Protein name	Gene name	Log2 +Tet vs -Tet	Log2 siEWS-FLI1 vs siCtrl
P56199	Integrin alpha-1	ITGA1	-0.80	0.96
Q13683	Integrin alpha-7	ITGA7	-0.64	N/A
P38570	Integrin alpha-E	ITGAE	-1.61	N/A
P06756	Integrin alpha-V	ITGAV	-0.62	0.15
P05556	Integrin beta-1	ITGB1	-0.42	0.69
P18084	Integrin beta-5	ITGB5	-1.01	-0.42
P18564	Integrin beta-6	ITGB6	-0.74	N/A

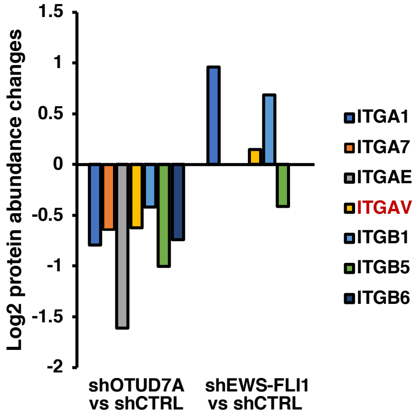

G

Collagen protein abundance changes in response to shOTUD7A				
ID	Protein name	Gene name	Log2 +Tet vs -Tet	-Log +Tet vs -Tet p-value
P02461	Collagen alpha-1(III) chain	COL3A1	-0.83	2.931775978
P02462	Collagen alpha-1(IV) chain	COL4A1	-0.80	3.989007302
P20908	Collagen alpha-1(V) chain	COL5A1	-0.71	3.569362292
P05997	Collagen alpha-2(V) chain	COL5A2	-0.62	2.213437336
P12107	Collagen alpha-1(XI) chain	COL11A1	-1.79	3.510074952
Q96P44	Collagen alpha-1(XXI) chain	COL21A1	-0.87	2.591040396

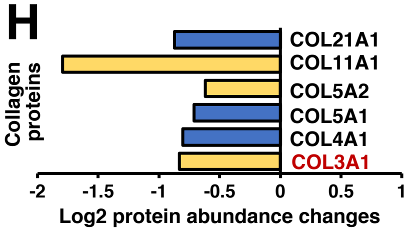

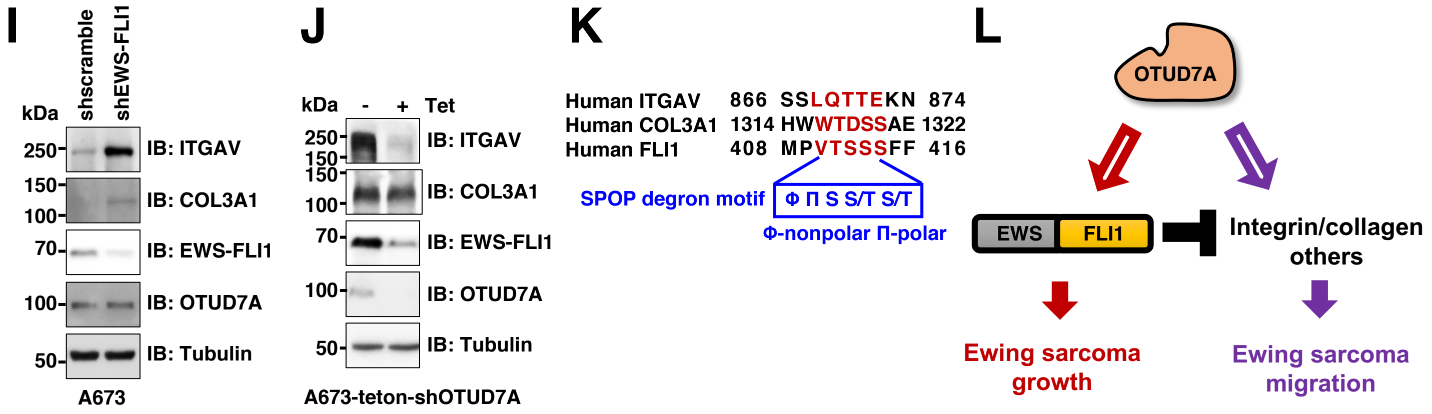

Fig. S13. Depletion of *OTUD7A* reduces Ewing sarcoma migration.
(A, B) Representative images for *in vitro* transwell assays (A) using either control or *EWS-FLI1 (and FLI1)* depleted A673 cells quantified in (B). Error bars were calculated as mean+/-SD, n=4. *p<0.05 (one-way *ANOVA* test). The scale bar represents 50 μm.
(C, D) Representative images for *in vitro* transwell assays (C) using either control or *OTUD7A* depleted A673 cells quantified in (B). Where indicated, 1 μg/mL tetracycline was added into cell culture for 72 hrs. Error bars were calculated as mean+/-SD, n=4. *p<0.05 (one-way *ANOVA* test). The scale bar represents 50 μm.
(E, F, G, H) Analyses of our quantitative proteomic data with a previously published siEWS-FLI1 quantitative proteomic dataset [1] lead to identification of indicated integrins and collagens as possible targets through which OTUD7A controls Ewing sarcoma metastasis.

(I) IB analyses of WCL derived from A673 cells depleted of endogenous *EWS-FLI1*.
(J) IB analyses of WCL derived from A673 cells treated with 1 μg/mL tetracycline for 3 days before cell collection.
(K) Protein sequence alignment showing possible SPOP degrons in ITGAV and COL3A1.
(L) A proposed model showing that OTUD7A controls Ewing sarcoma growth through regulating EWS-FLI1 protein stability, and also regulates Ewing sarcoma migration by regulating EWS-FLI1 independent substrates such as ITGAV and COL3A1.

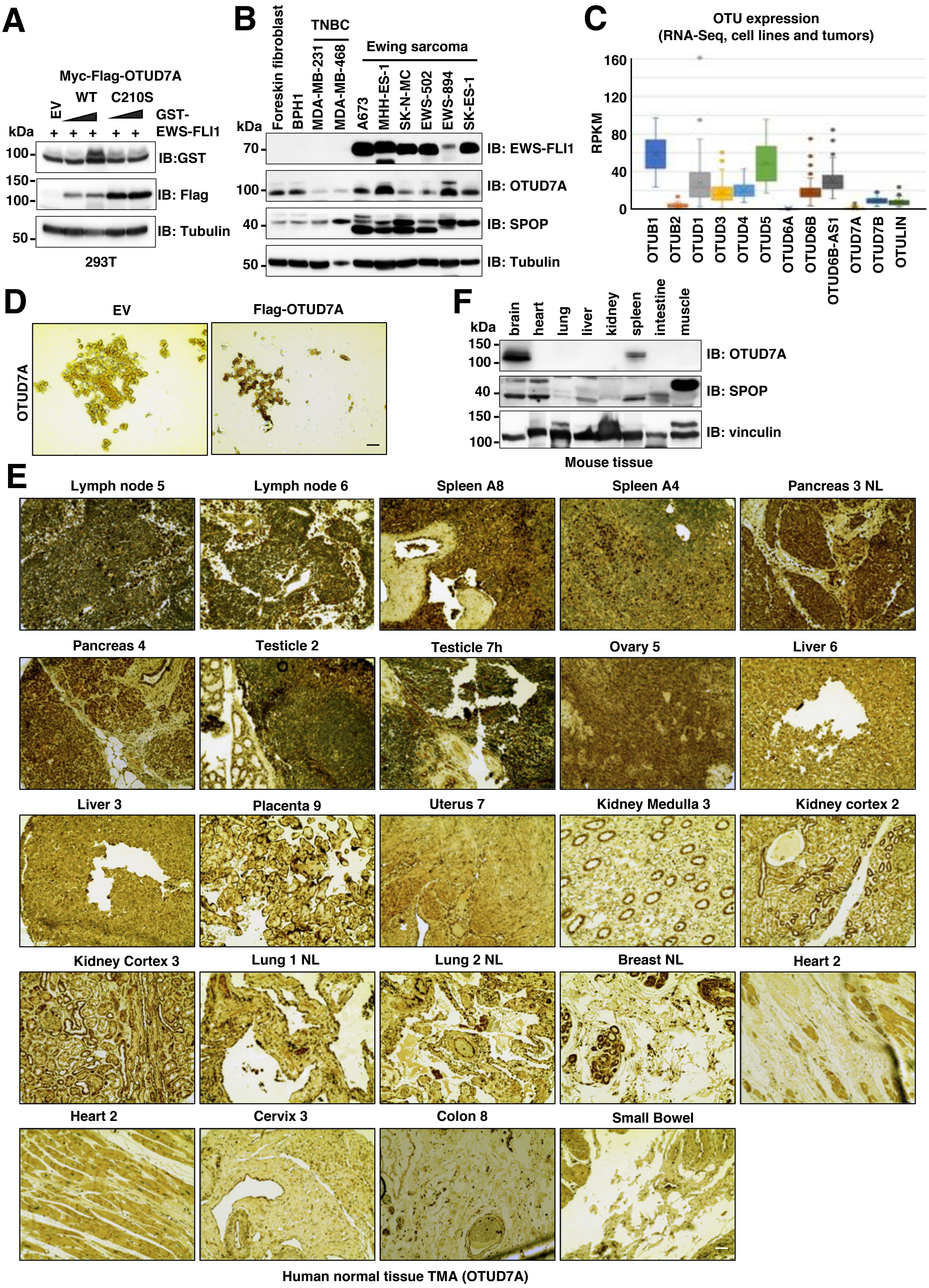
A
Myc-Flag-OTUD7A
EV
WT
C210S
GST-
EWS-FLI1
kDa
100
IB:GST
150
100
IB: Flag
50
IB: Tubulin
293T
B
Foreskin fibroblast
BPH1
TNBC
MDA-MB-231
MDA-MB-468
Ewing sarcoma
A673
MHH-ES-1
SK-N-MC
EWS-502
EWS-894
SK-ES-1
kDa
70
IB: EWS-FLI1
100
IB: OTUD7A
40
IB: SPOP
50
IB: Tubulin
C
OTU expression
(RNA-Seq, cell lines and tumors)
RPKM
160
120
80
40
0
OTUB1
OTUB2
OTUD1
OTUD3
OTUD4
OTUD5
OTUD6A
OTUD6B
OTUD6B-AS1
OTUD7A
OTUD7B
OTULIN
D
EV
Flag-OTUD7A
OTUD7A
F
kDa
brain
heart
lung
liver
kidney
spleen
intestine
muscle
150
100
IB: OTUD7A
40
IB: SPOP
150
100
IB: vinculin
Mouse tissue
E
Lymph node 5
Lymph node 6
Spleen A8
Spleen A4
Pancreas 3 NL
Pancreas 4
Testicle 2
Testicle 7h
Ovary 5
Liver 6
Liver 3
Placenta 9
Uterus 7
Kidney Medulla 3
Kidney cortex 2
Kidney Cortex 3
Lung 1 NL
Lung 2 NL
Breast NL
Heart 2
Heart 2
Cervix 3
Colon 8
Small Bowel
Human normal tissue TMA (OTUD7A)

Fig. S14. Examination of OTUD7A expression in Ewing sarcoma cell lines and tumors.
(A) IB analyses of WCL derived from HEK293T cells transfected with indicated DNA constructs.
(B) IB analyses of WCL derived from various cell lines as indicated.
(C) Analyses of OTUD7A mRNA levels in Ewing sarcoma cell lines and Ewing sarcoma patient tissues.
(D) Validation of OTUD7A antibody in detecting a specific OTUD7A signal using HEK293T cell transfected with or without an OTUD7A construct. 2 days post-transfection, indicated 293T cells were fixed and made into FFPE cell blocks, which were cut into slides for IHC staining using an OTUD7A antibody. The scale bar represents 100 μm.
(E) Representative IHC staining images by the validated OTUD7A antibody using a normal human tissue microarray. The scale bar represents 25 μm.
(F) IB analyses of WCL derived from indicated mouse tissues.

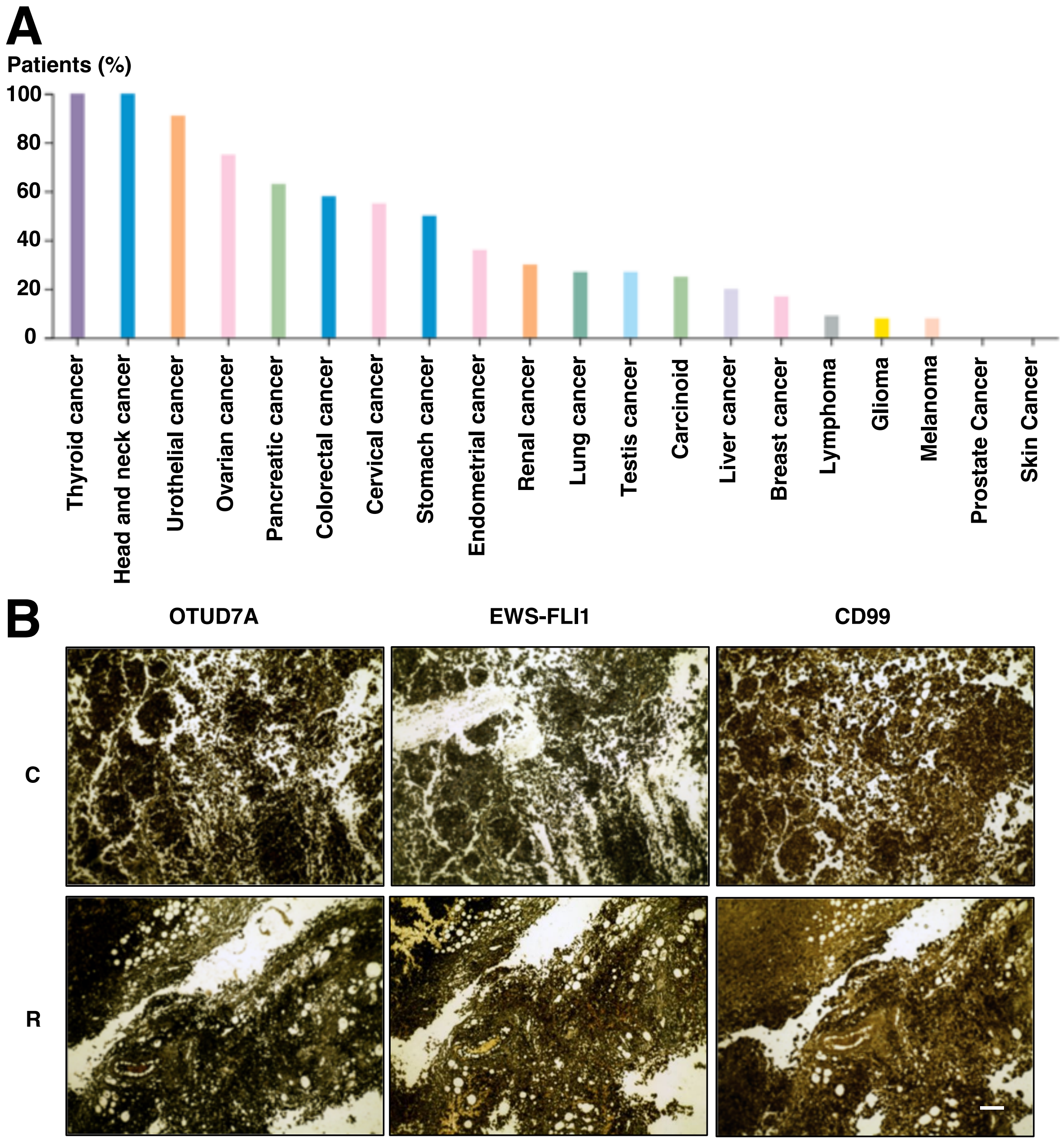

Fig. S15. OTUD7A expression is observed in Ewing sarcoma patient tumors.
(A) A summary of OTUD7A protein expression obtained from protein atlas (https://www.proteinatlas.org/ENSG00000169918-OTUD7A/pathology).
(B) Representative IHC staining images by the validated OTUD7A antibody, a FLI1 antibody and a CD99 antibody as indicated using two tumors obtained from an Ewing sarcoma patient. The scale bar represents 25 μm.

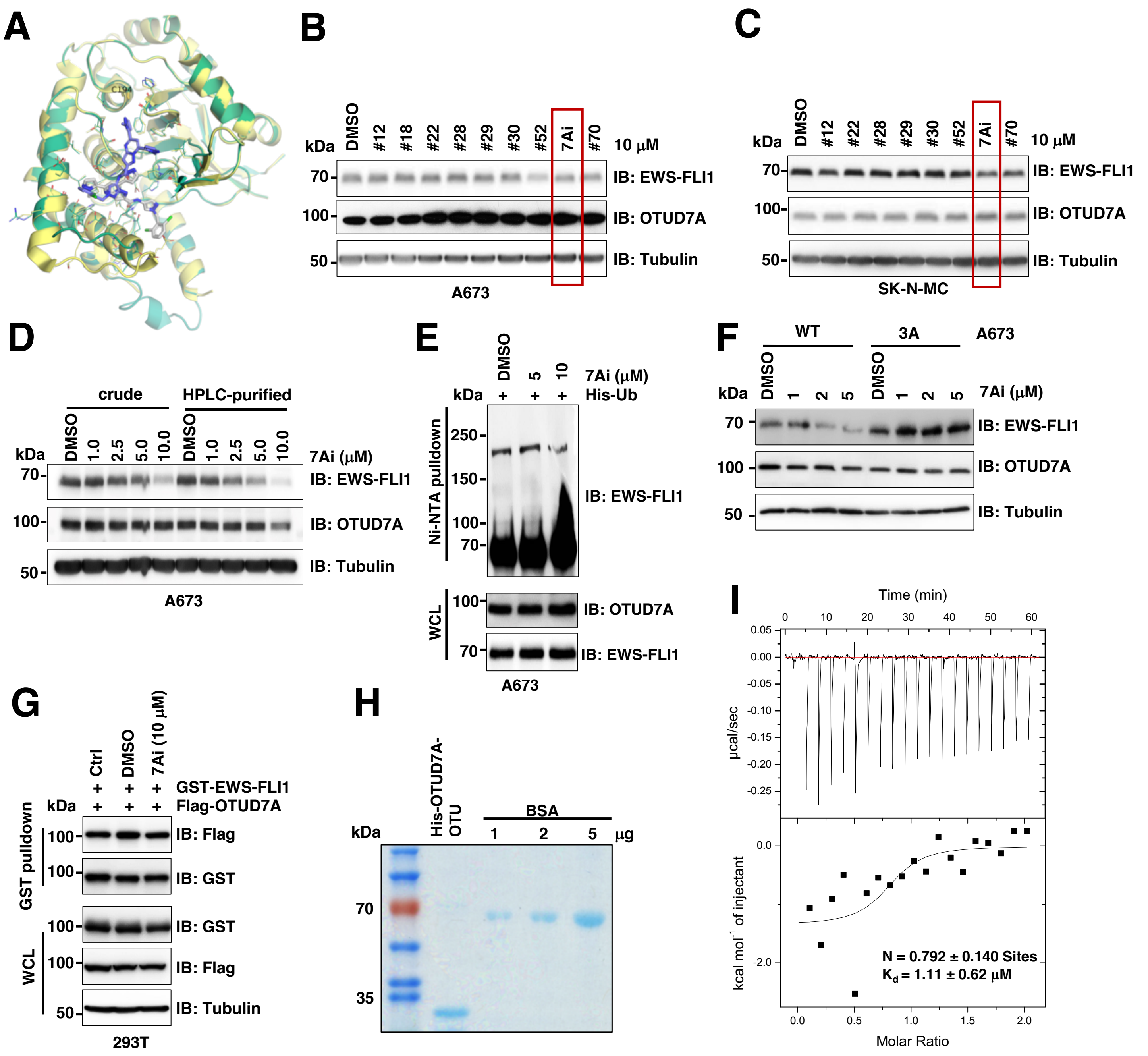

Fig. S16. Development and characterization of compound 7Ai as a lead compound as an OTUD7A catalytic inhibitor.

(A) The distal ubiquitin-binding site S1 on OTUD7A (green), modeled after the PDB structure 5LRW from OTUD7B (yellow), which was chosen for the virtual screen. Two potential ligand poses are shown for 7Ai in blue and white. The label shows the location of the catalytic residue (C194 in OTUD7B, C210 in OTUD7A).

(B, C) IB analyses of WCL derived from A673 (B) or SK-N-MC (C) cells treated with indicated compounds for 12 hrs at 10 □M concentrations.

(D) IB analyses of WCL derived from A673 cells treated with non-purified or HPLC-purified compound 7Ai at indicated doses for 12 hrs.

(E) IB analyses of Ni-NTA pulldowns and WCL derived from indicated A673 cells treated with compound 7Ai at indicated doses for 12 hrs.

(F) IB analyses of WCL derived from indicated A673 cells treated with compound 7Ai at indicated doses for 12 hrs.
(G) IB analyses of GST-pulldown and WCL derived from HEK293T cells transfected with indicated DNA constructs.
(H) A representative gel-cod stained SDS-PAGE gel showing expression of his-tagged OTUD7A-OTU domain proteins.
(I) ITC analysis of 7Ai binding to his-OTUD7A-OTU proteins *in vitro*.

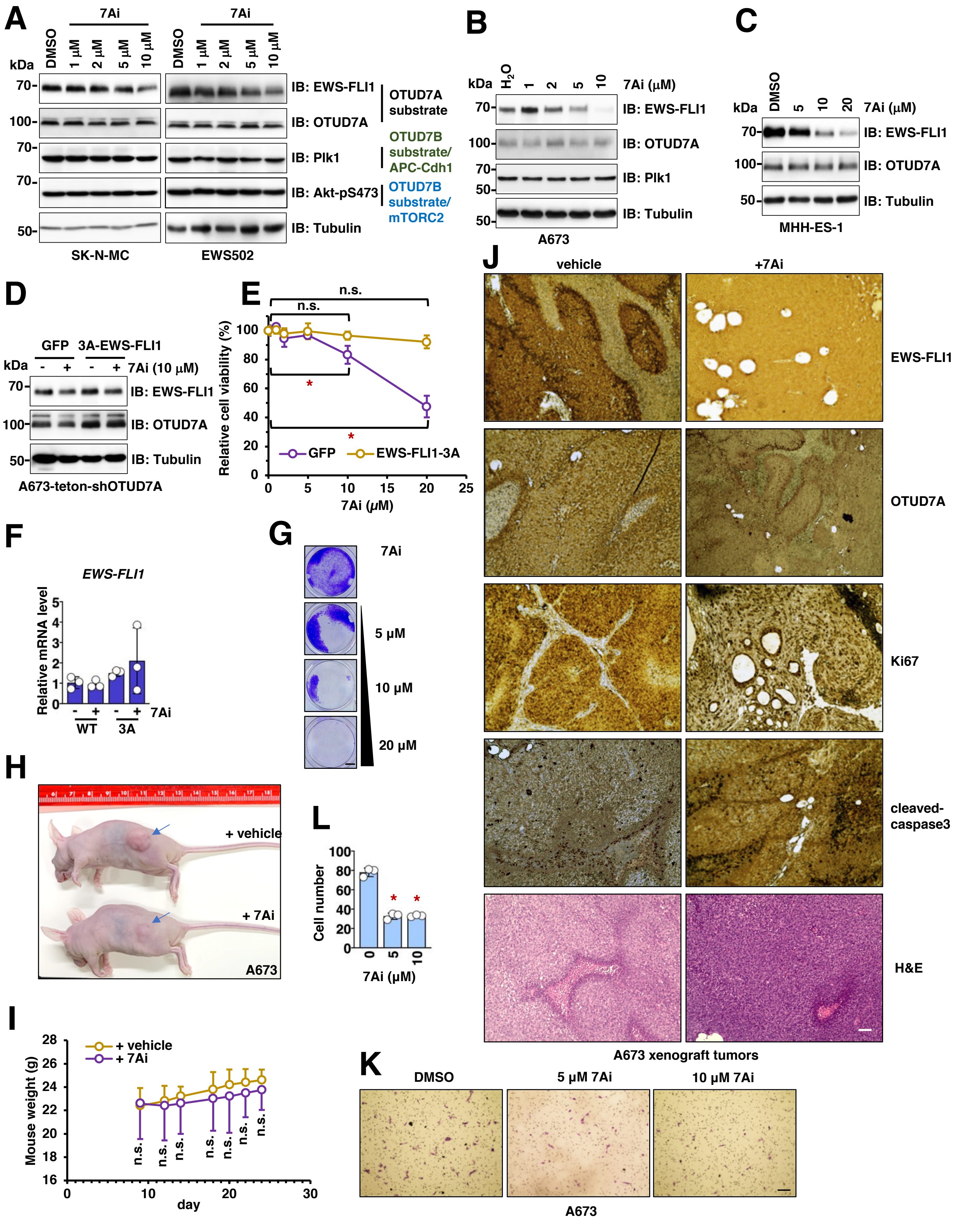

A
7Ai
DMSO
1 µM
2 µM
5 µM
10 µM
kDa
IB: EWS-FLI1
IB: OTUD7A
IB: Plk1
IB: Akt-pS473
IB: Tubulin
OTUD7A substrate
OTUD7B substrate/ APC-Cdh1
OTUD7B substrate/ mTORC2
SK-N-MC
EWS502
B
7Ai (µM)
A673
C
MHH-ES-1
D
GFP
3A-EWS-FLI1
7Ai (10 µM)
A673-teton-shOTUD7A
E
n.s.
Relative cell viability (%)
GFP
EWS-FLI1-3A
7Ai (µM)
F
EWS-FLI1
Relative mRNA level
WT
3A
G
5 µM
10 µM
20 µM
H
+ vehicle
+ 7Ai
A673
I
Mouse weight (g)
day
J
vehicle
+7Ai
EWS-FLI1
OTUD7A
Ki67
cleaved-caspase3
H&E
A673 xenograft tumors
K
DMSO
5 µM 7Ai
10 µM 7Ai
L
Cell number

Fig. S17. 7Ai treatment reduced EWS-FLI1 protein expression and Ewing sarcoma growth.
(A, B, C) IB analyses of WCL derived from SK-N-MC and EWS502 cells (A), A673 (B) or MHH-ES-1 (C) treated with indicated doses of compound 7Ai for 12 hrs before cell collection.
(D) IB analyses of WCL derived from A673-teton-shOTUD7A cells reconstituted with either GFP or EWS-FLI1-3A, treated with or without 10 μM of 7Ai overnight.
(E) Cell viability assays of A673-teton-shOTUD7A cells reconstituted with either GFP or EWS-FLI1-3A. 2,000 cells were plated into each well in 96-well plates and treated with indicated doses of 7Ai. Cell viability was determined 3 days post-7Ai addition. Error bars were calculated as mean+/-SD, n=5. *$p<0.05$ (one-way *ANOVA* test).
(F) RT-PCR analysis of *EWS-FLI1* mRNA changes upon 12 hours of 10 μM 7Ai treatment in indicated cells. Error bars were calculated as mean+/-SD, n=3. $p>0.05$ (one-way *ANOVA* test) and no significance was found.
(G) Representative images for colony formation assays using A673 cells treated with indicated compound 7Ai for 3 weeks. The scale bar represents 10 mm.
(H, I) Mouse xenograft experiments were performed with indicated A673 cells treated with vehicle or 7Ai. 7-day post-injection when tumors were established in mice, 7Ai (25 mg/kg) was injected into mice through IP route every 2-3 days. 25 days post-injection, mice were sacrificed, and tumors were dissected and subjected to IHC staining by indicated antibodies (I). The scale bar represents 25 *μ*m.
(J) 7Ai treatment in a 25-day period does not cause weight loss in nude mice. No significant body weight changes were observed in mice used in (F). Error bars were calculated as mean+/-SD, n=10. $p>0.05$ (one-way *ANOVA* test) and no significance was found.
(K, L) Representative images for *in vitro* transwell assays (K) using A673 cells treated with vehicle or 7Ai for 72 hrs and quantified in (L). Error bars were calculated as mean+/-SD, n=3. *$p<0.05$ (one-way *ANOVA* test). The scale bar represents 50 μm.

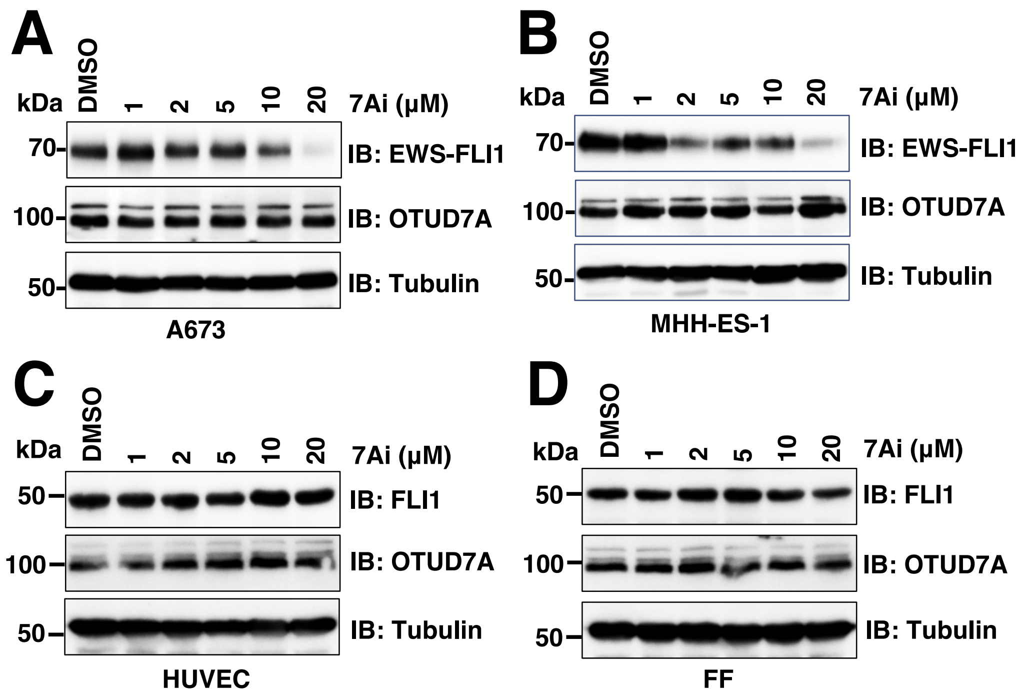

Fig. S18. 7Ai treatment reduced EWS-FLI1 protein expression in Ewing sarcoma but does not change FLI expression in normal control cells.
(A, B, C, D) IB analyses of WCL derived from A673 (A), MHH-ES-1 (B), HUVEC (C) or FF (D, foreskin fibroblast) cells treated with indicated doses of 7Ai for 3 days.

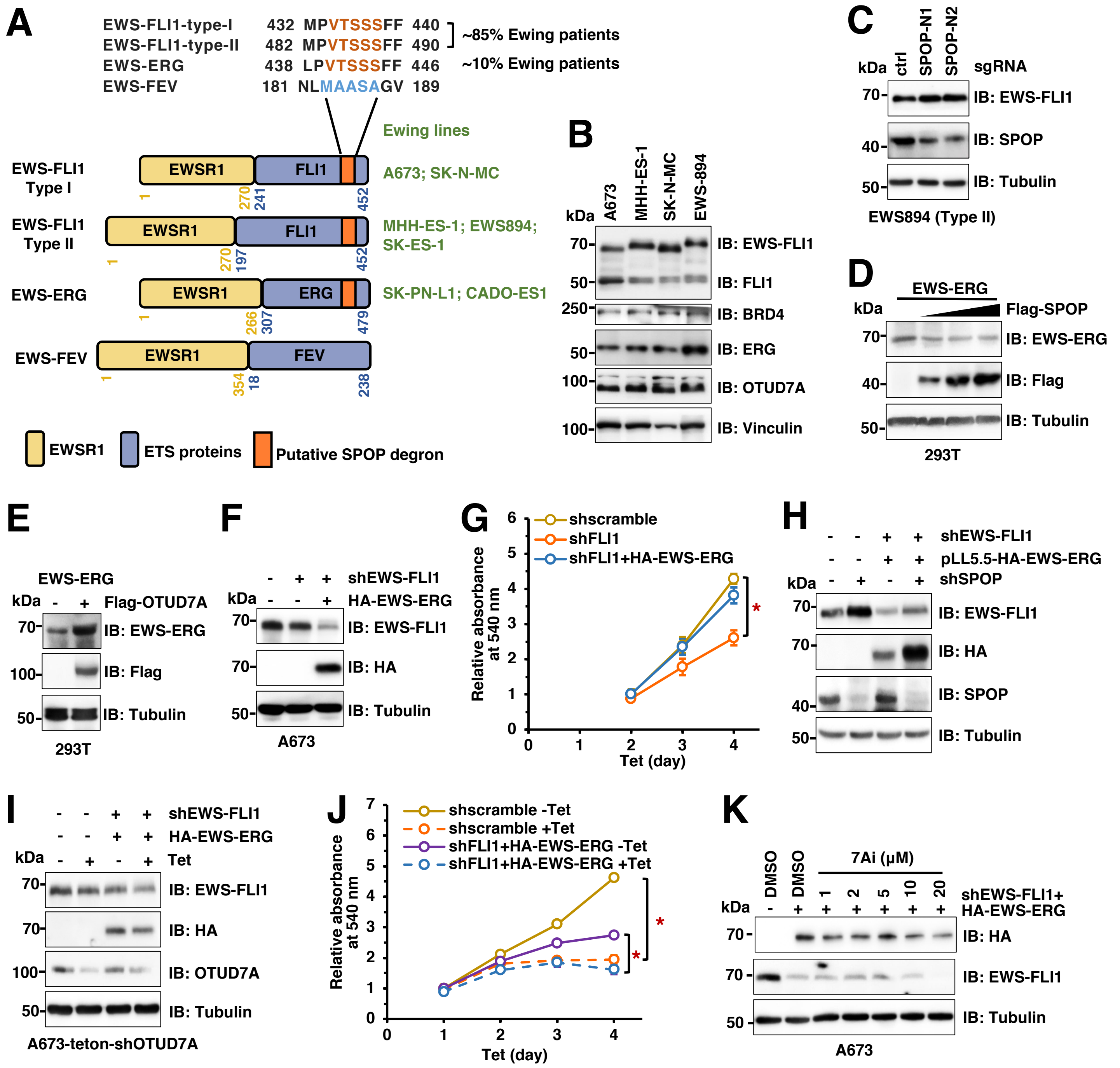

Fig. S19. OTUD7A may also regulate EWS-ERG protein stability.

(A) A cartoon illustration of both type I and type II EWS-FLI1 fusion types, and other fusion genes including EWS-ERG and EWS-FEV. Possible SPOP degron sequence is observed in EWS-ERG but not EWS-FEV fusion. Ewing sarcoma cell lines bearing different fusions are also marked in green.

(B) IB analysis of WCL derived from indicated Ewing sarcoma cells.

(C) IB analysis of WCL derived from EWS894 cells depleted of endogenous SPOP by two independent sgRNAs.

(D) IB analysis of WCL derived from HEK293T cells transfected with indicated DNA constructs.

(E) IB analysis of WCL derived from HEK293T cells transfected with indicated DNA constructs.

(F) IB analysis of WCL derived from A673 cells infected with indicated viruses. Cells were collected 3 days post-infection.

(G) Cell viability assays using cells obtained in (F) with indicated periods. Error bars were calculated

as mean+/-SD, n=5. *$p<0.05$ (one-way *ANOVA* test).
(H) IB analysis of WCL derived from A673 cells infected with indicated viruses. Cells were collected 3 days post-infection.
(I) IB analysis of WCL derived from A673-teton-shOTUD7A cells infected with indicated viruses. Cells were collected 3 days post-1 μg/mL tetracycline treatment.
(J) Cell viability assays using cells obtained in (I) with indicated periods. Error bars were calculated as mean+/-SD, n=5. *$p<0.05$ (one-way *ANOVA* test).
(K) IB analyses of WCL obtained from indicated A673 cells treated with indicated doses of 7Ai for 12 hrs before cell collection.

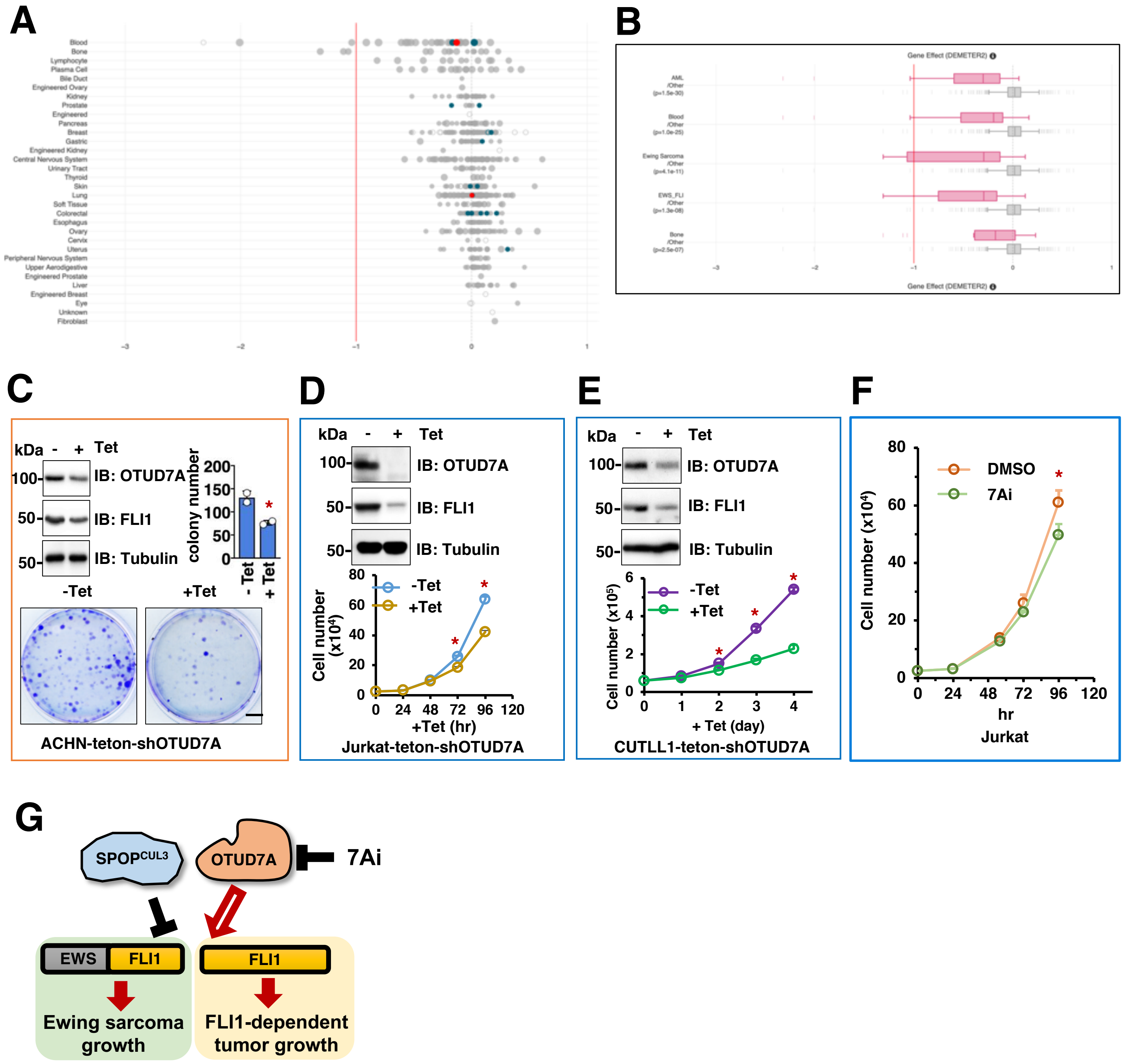

Fig. S20. Inactivation of OTUD7A reduces FLI1 expression and suppresses FLI-dependent cell growth.

(A-B) Evaluation of cell growth dependence on FLI1 by CRISPR in indicated cancer types from DEPMAP portal. Each dot represents a cell line.

(C-E) Tet-induced depletion of *OTUD7A* led to reduced endogenous FLI1 protein abundance in indicated cells and subsequently reduced cell growth. Error bars were calculated as mean+/-SD, n=2 for C and 3 for D, E. *p<0.05 (one-way *ANOVA* test). The scale bar represents 10 mm.

(F) Treatment of Jurkat cells with compound 7Ai led to reduced cell proliferation *in vitro*. Error bars were calculated as mean+/-SD, n=3. *p<0.05 (one-way *ANOVA* test).

(G) A proposed model that SPOP and OTUD7A controls EWS-FLI1 degradation and deubiquitination, respectively, to govern Ewing sarcoma growth. Pharmacological inhibition of OTUD7A may represent a novel therapeutic direction for Ewing sarcoma treatment by downregulating EWS-FLI1 protein abundance.

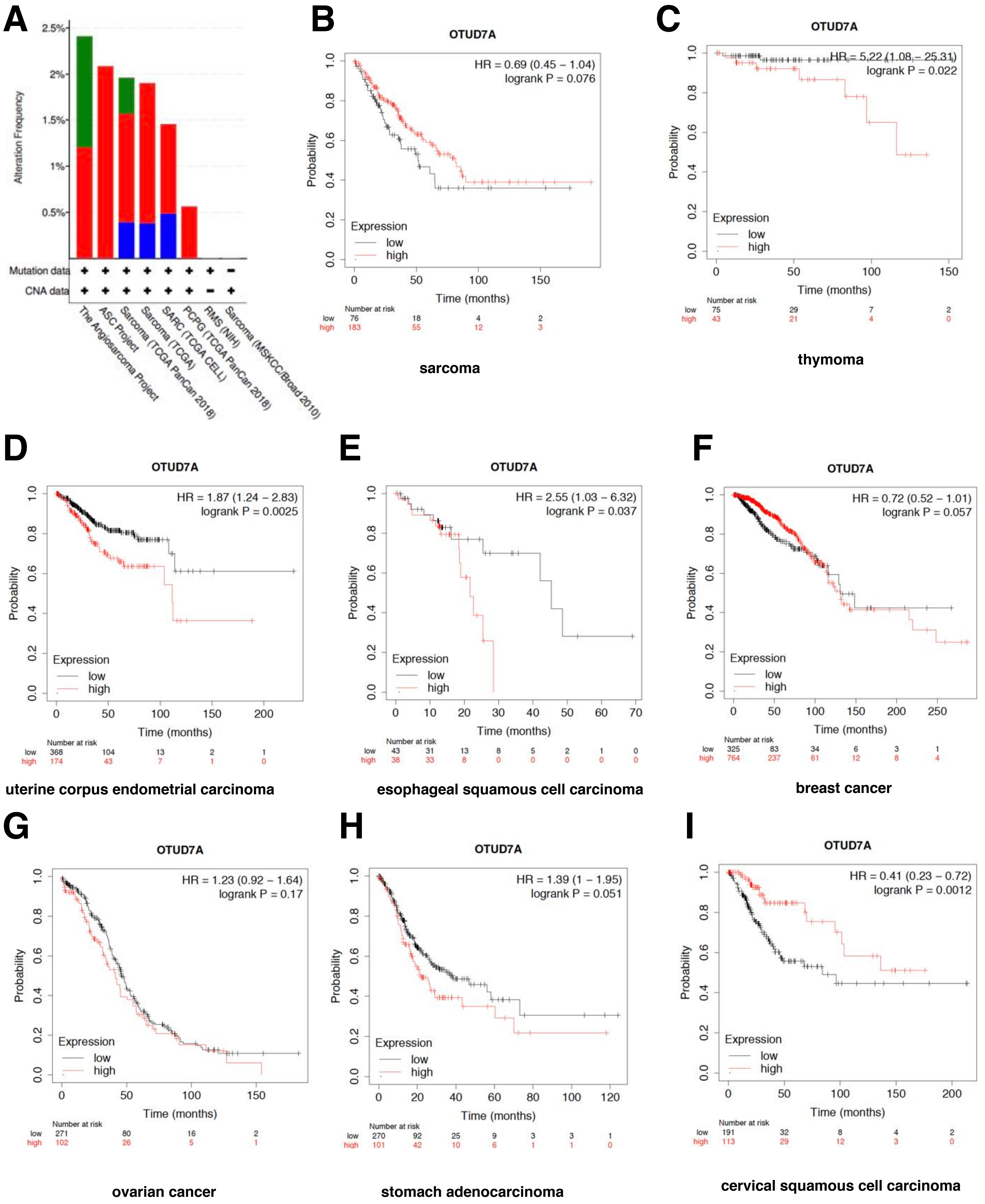
A
Alteration Frequency
2.5%
2%
1.5%
1%
0.5%
Mutation data
CNA data
The Angiosarcoma Project
ASC Project
Sarcoma (TCGA PanCan 2018)
Sarcoma (TCGA)
SARC (TCGA CELL)
PCPG (TCGA PanCan 2018)
RMS (NIH)
Sarcoma (MSKCC/Broad 2010)
B
OTUD7A
HR = 0.69 (0.45 − 1.04)
logrank P = 0.076
Probability
Time (months)
Expression
low
high
Number at risk
low 76 18 4 2
high 183 55 12 3
sarcoma
C
OTUD7A
HR = 5.22 (1.08 − 25.31)
logrank P = 0.022
Probability
Time (months)
Expression
low
high
Number at risk
low 75 29 7 2
high 43 21 4 0
thymoma
D
OTUD7A
HR = 1.87 (1.24 − 2.83)
logrank P = 0.0025
Probability
Time (months)
Expression
low
high
Number at risk
low 368 104 13 2 1
high 174 43 7 1 0
uterine corpus endometrial carcinoma
E
OTUD7A
HR = 2.55 (1.03 − 6.32)
logrank P = 0.037
Probability
Time (months)
Expression
low
high
Number at risk
low 43 31 13 8 5 2 1 0
high 38 33 8 0 0 0 0 0
esophageal squamous cell carcinoma
F
OTUD7A
HR = 0.72 (0.52 − 1.01)
logrank P = 0.057
Probability
Time (months)
Expression
low
high
Number at risk
low 325 83 34 6 3 1
high 764 237 61 12 8 4
breast cancer
G
OTUD7A
HR = 1.23 (0.92 − 1.64)
logrank P = 0.17
Probability
Time (months)
Expression
low
high
Number at risk
low 271 80 16 2
high 102 26 5 1
ovarian cancer
H
OTUD7A
HR = 1.39 (1 − 1.95)
logrank P = 0.051
Probability
Time (months)
Expression
low
high
Number at risk
low 270 92 25 9 3 3 1
high 101 42 10 6 1 1 0
stomach adenocarcinoma
I
OTUD7A
HR = 0.41 (0.23 − 0.72)
logrank P = 0.0012
Probability
Time (months)
Expression
low
high
Number at risk
low 191 32 8 4 2
high 113 29 12 3 0
cervical squamous cell carcinoma

Fig. S21. Analyses of expression of OTUD7A in pan-cancer.
(A) An analysis of *OTUD7A* DNA alternations in TCGA sarcoma patient dataset obtained from cbioportal. Red, gene amplification; blue, gene deletion; green, gene mutation.
(B-I) Analyses of *OTUD7A* mRNA expression association with overall patient survival in indicated cancer types. Data were obtained from https://kmplot.com/analysis/index.php?p=service.

Table S2. 99 characterized EWS-FLI1 targets down-regulated upon *OTUD7A* depletion with log2 fold changes > 0.5

Gene name	Log2 fc shOTUD7A vs control	Gene name	Log2 fc shOTUD7A vs control	Gene name	Log2 fc shOTUD7A vs control
FOXM1	-0.49	ARHGEF6	-0.29	SERBP1	-0.20
SMARCC1	-0.48	HMGB3	-0.29	AURKB	-0.19
RRM1	-0.48	SLBP	-0.29	CACNB2	-0.19
EXO1	-0.48	TNS1	-0.28	AKAP8L	-0.18
RAD51	-0.48	TRIM2	-0.28	NR0B1	-0.18
PTPN13	-0.47	R3HDM1	-0.28	CHAF1B	-0.17
GNPAT	-0.47	SRR	-0.27	HELLS	-0.17
NELL2	-0.46	UPF3B	-0.27	CEBPB	-0.16
PTTG1IP	-0.46	SOX11	-0.27	CHAF1A	-0.16
KIF14	-0.46	EXOSC2	-0.27	MYO10	-0.16
ID2	-0.44	GYG2	-0.27	FNBP1	-0.15
NEO1	-0.44	RAD51C	-0.27	USP20	-0.15
GGA2	-0.42	POLD3	-0.26	CAB39	-0.15
ARHGAP19	-0.41	PRIM1	-0.26	PAICS	-0.14
RCOR1	-0.41	KHDRBS1	-0.25	MCM4	-0.13
RAP1GAP	-0.41	PPP1R1A	-0.25	BLM	-0.13
CDT1	-0.41	PBX2	-0.24	DDX46	-0.12
JAK1	-0.40	NUP205	-0.24	CAD	-0.12
DCLRE1A	-0.40	TAF5	-0.23	TMPO	-0.11
WDHD1	-0.40	EPB41	-0.23	GNL3	-0.11
SSH1	-0.39	RFC1	-0.23	NFATC2IP	-0.11
CHEK1	-0.38	PRC1	-0.23	CSE1L	-0.11
FANCA	-0.38	MAD2L1BP	-0.23	PHGDH	-0.09
MYC	-0.36	PTBP1	-0.22	FBL	-0.09
CBX5	-0.34	KIF2C	-0.22	RBMX	-0.09
AURKA	-0.32	CEP55	-0.22	SKP2	-0.09
PASK	-0.32	NCAPH	-0.22	RAE1	-0.09
RFC5	-0.32	TOP2A	-0.22	TCERG1	-0.08
RAD54B	-0.31	PLK1	-0.22	SMC4	-0.08
POLE2	-0.31	NUPL1	-0.21	HOOK1	-0.07
KIF15	-0.30	TIMELESS	-0.20	GMPS	-0.06
RGL1	-0.30	POU3F1	-0.20	UAP1	-0.04
AKAP7	-0.30	RMI1	-0.20		

Supplemental References

1. Tanabe, Y., et al., *IRE1alpha-XBP1 inhibitors exerted anti-tumor activities in Ewing's sarcoma.* Oncotarget, 2018. **9**(18): p. 14428-14443.
